# Extrachromosomal DNA–Driven Oncogene Spatial Heterogeneity and Evolution in Glioblastoma

**DOI:** 10.1158/2159-8290.CD-24-1555

**Published:** 2025-09-08

**Authors:** Imran Noorani, Magnus Haughey, Jens Luebeck, Andrew Rowan, Eva Grönroos, Francesco Terenzi, Ivy Tsz-Lo Wong, Davide Pradella, Marta Lisi, Jeanette Kittel, Natasha Sharma, Chris Bailey, Clare E. Weeden, Donald M. Bell, Eric Joo, Vittorio Barbè, Matthew G. Jones, King L. Hung, Emma L. Nye, Mary Green, Lucy Meader, Emma J. Norton, Mark Fabian, Nnennaya Kanu, Mariam Jamal-Hanjani, Thomas Santarius, Andrea Ventura, James A.R. Nicoll, Delphine Boche, Howard Y. Chang, Vineet Bafna, Weini Huang, Paul S. Mischel, Charles Swanton, Benjamin Werner

**Affiliations:** 1 Cancer Evolution and Genome Instability Laboratory, The Francis Crick Institute, London, United Kingdom.; 2 Department of Neurosurgery, National Hospital for Neurology and Neurosurgery, London, United Kingdom.; 3 Institute of Neurology, University College London, London, United Kingdom.; 4 Evolutionary Dynamics Group, Centre for Cancer Evolution, Barts Cancer Institute, Queen Mary University of London, London, United Kingdom.; 5 Department of Computer Science and Engineering, University of California, San Diego, La Jolla, California.; 6 Sarafan ChEM-H, Stanford University, Stanford, California.; 7 Department of Pathology, Stanford University, Stanford, California.; 8 Cancer Biology and Genetics Program, Memorial Sloan Kettering Cancer Center, New York, New York.; 9 Center for Stem Cell Biology and Developmental Biology Program, Memorial Sloan Kettering Cancer Center, New York, New York.; 10 UCL Cancer Institute, London, United Kingdom.; 11 Crick Advanced Light Microscopy, The Francis Crick Institute, London, United Kingdom.; 12 Center for Personal Dynamic Regulomes, Stanford University, Stanford, California.; 13 Department of Neuroscience, Scripps Research, La Jolla, California.; 14 Experimental Histopathology, The Francis Crick Institute, London, United Kingdom.; 15 Department of Cellular Pathology, University Hospital Southampton NHS Foundation Trust, Southampton, United Kingdom.; 16 Clinical Neurosciences, Clinical and Experimental Sciences, Faculty of Medicine, University of Southampton, Southampton, United Kingdom.; 17 Cancer Research UK Lung Cancer Centre of Excellence, University College London Cancer Institute, London, United Kingdom.; 18 Cancer Metastasis Laboratory, University College London Cancer Institute, London, United Kingdom.; 19 Department of Medical Oncology, University College London Hospitals, London, United Kingdom.; 20 Department of Neurosurgery, Cambridge University Hospital, Cambridge, United Kingdom.; 21 Departments of Dermatology, Stanford University School of Medicine, Stanford, California.; 22 Departments of Genetics, Stanford University School of Medicine, Stanford, California.; 23 Amgen Research, South San Francisco, California.; 24 Halıcıoğlu Data Science Institute, University of California, San Diego, California.; 25 Department of Mathematics, Queen Mary University of London, London, United Kingdom.; 26 Group of Theoretical Biology, The State Key Laboratory of Biocontrol, School of Life Science, Sun Yat-sen University, Guangzhou, China.; 27 Department of Oncology, University College London Hospitals, London, United Kingdom.

## Abstract

Oncogenes amplified on extrachromosomal DNA (ecDNA) contribute to treatment resistance and poor survival across cancers. Currently, the spatiotemporal evolution of ecDNA remains poorly understood. In this study, we integrate computational modeling with samples from 94 treatment-naive human glioblastomas (GBM) to investigate the spatiotemporal evolution of ecDNA. We observe oncogene-specific patterns of ecDNA spatial heterogeneity, emerging from random ecDNA segregation and differing fitness advantages. Unlike *PDGFRA*-ecDNAs, *EGFR*-ecDNAs often accumulate prior to clonal expansions, conferring strong fitness advantages and reaching high abundances. In corroboration, we observe pretumor ecDNA accumulation *in vivo* in genetically engineered mouse neural stem cells. Variant and wild-type *EGFR*-ecDNAs often coexist in GBM. Those variant *EGFR*-ecDNAs, most commonly *EGFRvIII*-ecDNA, always derive from preexisting wild-type *EGFR*-ecDNAs, occur early, and reach high abundance. Our results suggest that the ecDNA oncogenic makeup determines unique evolutionary trajectories. New concepts such as ecDNA clonality and heteroplasmy require a refined evolutionary interpretation of genomic data in a large subset of GBMs.

**Significance:**

We study spatial patterns of ecDNA-amplified oncogenes and their evolutionary properties in human GBM, revealing an ecDNA landscape and ecDNA oncogene–specific evolutionary histories. ecDNA accumulation can precede clonal expansion, facilitating the emergence of *EGFR* oncogenic variants, reframing our interpretation of genomic data in a large subset of GBMs.

*See related commentary by Korsah et al., p. 1979*

## Teaser

Extrachromosomally amplified oncogenes are shown to drive evolution in a large subset of *IDH* wild-type glioblastomas, and the spatial patterns of extrachromosomal DNA contain information on a tumor’s evolutionary history.

## Introduction

Genetic intratumor heterogeneity pervades all human cancers (1, 2). It emerges through cell division, mutation accumulation, and clonal selection, enabling somatic evolutionary processes, including mutagenesis (3), that contribute to progression and treatment resistance. Oncogene amplifications on circular extrachromosomal DNAs (ecDNA) are a major driver of heterogeneity (4), with random ecDNA segregation enabling extreme cell-to-cell ecDNA copy-number variation upon which selection may act. ecDNA may arise early in the development of cancer, as has been shown for Barrett’s esophagus, in which the formation of ecDNA in high-grade dysplasia is tightly linked to the development of esophageal cancer (5).

Glioblastoma (GBM) is the most common primary intrinsic brain tumor in adults. Its prognosis has barely improved in the last few decades, with a median survival of only 14 months (6). Targeted therapies have so far failed to make meaningful improvements to survival for GBM in clinical practice (7–10). *IDH* wild-type (*IDH*wt) GBMs frequently harbor oncogenes on ecDNAs, such as *EGFR*, *PDGFRA*, and *CDK4* (11–14). It remains unclear what the evolutionary implications of these different ecDNA-amplified oncogenes are in individual tumors. This is, in part, because random ecDNA segregation complicates the reconstruction of the evolutionary histories of ecDNA-driven tumors (15, 16). To address these challenges, we developed a modeling approach called spatial ecDNA intratumor evolution simulation (SPECIES; spatial–temporal computational model of ecDNA-positive tumors) that uses structural variant (SV) analysis of focal amplifications from whole-genome sequencing (WGS), DNA FISH with unbiased quantitative image analysis, and RNAscope to measure nascent RNA transcription arising from oncogenes on ecDNA and spatial–temporal computer simulations to resolve the spatial organization of ecDNA and model its evolutionary trajectories. We applied this approach to 94 *IDH*wt GBM samples to quantify the incidence, structure, and spatial evolution of ecDNA in human GBM, revealing critical underlying biological differences dictated by oncogenes and certain variants carried on ecDNA.

## Results

### The Landscape of ecDNA-Amplified Oncogenes in Human GBM

**Figure 1.**
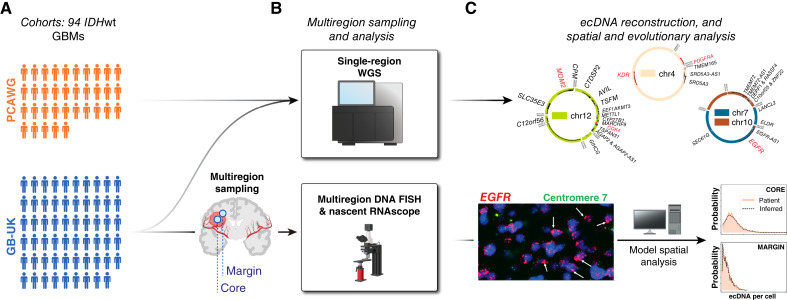
Workflow of the study analysis. **A,** A total of 94 *IDH*wt GBMs were analyzed (35 from the PCAWG cohort and 59 from the GB-UK cohort). **B,** The core region of all patient tumors was analyzed using WGS, and GB-UK patient tumors were further subjected to multiregion DNA FISH and nascent RNAscope analysis to characterize spatial patterns of oncogenic ecDNAs. **C,** The ecDNA genetic landscape and fine structure were analyzed using WGS information, and mathematical modeling of single-cell copy number was employed to reconstruct evolutionary histories in GB-UK patient tumors. (**B,** created using BioRender assets. https://BioRender.com/kgae4q5; https://BioRender.com/7f2pume)

**Figure 2.**
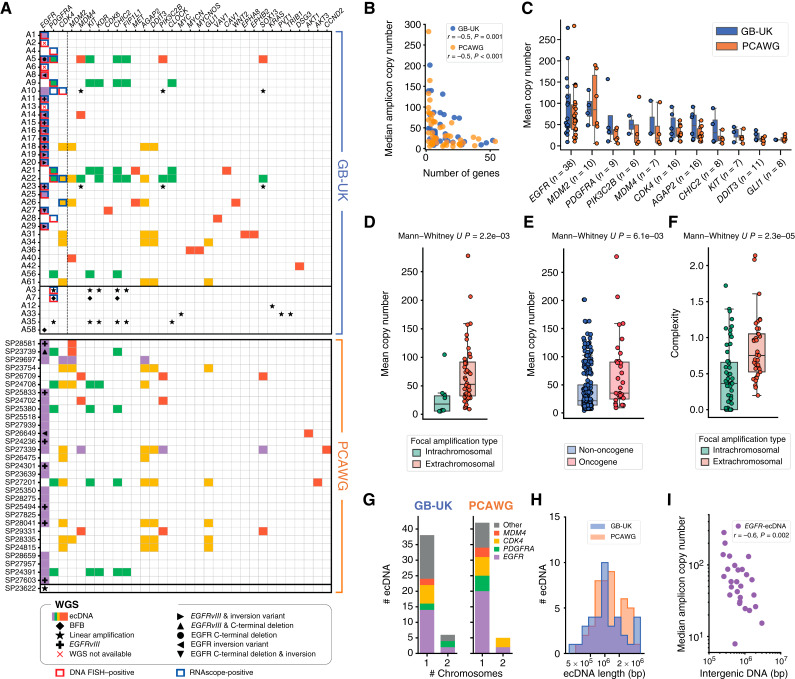
Diversity and high copy numbers of ecDNA in *IDH*wt GBMs. **A,** Focal copy-number amplifications across the GB-UK and PCAWG cohorts, categorized by amplicon type [ecDNA, breakage–fusion–bridge (BFB) cycle, linear]. Only tumors containing focal amplifications are shown. Oncogenes with the same colored box indicate they exist on the same ecDNA molecule. The horizontal line between patients A61 and A3 (GB-UK) and patients SP27603 and SP23622 (PCAWG) separates patient samples with (above) or without (below) ecDNA, detected by WGS, DNA FISH, or nascent RNAscope. Oncogenes tested with DNA FISH and/or nascent RNAscope, *EGFR*, *PDGFRA*, and *CDK4*, are separated to the left of the vertical dashed line. **B,** Median ecDNA copy number vs. number of ecDNA-amplified genes for GB-UK and PCAWG cohorts (Spearman correlation). **C,** Oncogenes contained within ecDNA in both GB-UK and PCAWG cohorts ranked by maximum copy number. The number of samples with each oncogene as ecDNA in the GB-UK cohort is described in brackets. Only copy numbers detected by WGS are shown here. **D,** Mean copy number of linear focal amplifications vs. ecDNA in GB-UK. **E,** Mean copy number of non-oncogenes compared with oncogenes amplified on ecDNA in GB-UK. **F,** Complexity score of linear focal amplifications vs. ecDNA in GB-UK. **G,** Number of uni- and multichromosomal ecDNAs in GB-UK and PCAWG cohorts. **H,** Distribution of ecDNA lengths in GB-UK and PCAWG cohorts. **I,** Median ecDNA copy number vs. length of intergenic DNA for *EGFR*-ecDNA in GB-UK and PCAWG cohorts (Spearman correlation).

To determine the ecDNA landscape in human GBM at clinical presentation, we first performed WGS and/or DNA FISH analysis of 59 samples from adult patients with newly diagnosed *IDH*wt GBM in the Glioblastoma-UK (GB-UK) cohort (Fig. 1A and B; Supplementary Fig. S1; see “Methods” for clinical details; ref. 17). We deciphered structural variation and ecDNA status in 49 WGS samples using AmpliconArchitect and AmpliconSuite (median sequencing coverage of 15×; refs. 18, 19); samples with insufficient library coverage were excluded. Formalin-fixed, paraffin-embedded (FFPE) analysis was conducted as published in ref. (20); see the “Methods” section (Fig. 1C). In line with previous reports (21–23), we detected at least one ecDNA amplification in 57% (*n* = 28/49) of samples (Fig. 2A). Those ecDNAs contained between 2 and 52 genes with a median of 9.5 genes per ecDNA (Fig. 2B). In contrast, intrachromosomal focally amplified oncogenes were less common (*n* = 8/49 patient tumors, 16%; Fig. 2A; Supplementary Fig. S1). To further validate these observations, we analyzed a secondary cohort of 35 *IDH*wt GBMs from a pan-cancer analysis of whole genomes (PCAWG; refs. 21, 24). In this study, we detected ecDNA in 86% (*n* = 30/35) of samples (Fig. 2A; Supplementary Fig. S2) and found only one case with a focal intrachromosomal amplification. All but two observed ecDNAs contained at least one known oncogene. In GB-UK, focal *EGFR* amplifications were always on ecDNA (*n* = 16/49, 33%), except for one case of a breakage–fusion–bridge cycle (copy number = 6). Nonfocal chromosomal *EGFR* amplifications were less frequent (*n* = 5/49, 10%). Focal amplifications of other oncogenes, including *MDM2*, *CDK4*, *CDK6*, *MET*, *NMYC*, *AGAP2*, *DDIT3*, and *CLOCK*, occurred exclusively on ecDNA (Fig. 2A). Of 28 samples, 26 contained at least one distal enhancer sequence on their ecDNA (with 492 distal enhancers in total across all ecDNAs; see Supplementary Fig. S3). We observed immunomodulatory genes *C1QA*, *C1QB*, and *C1QC* on ecDNA in the tumor of GB-UK patient A31, as well as one case with *IFNG* in the PCAWG dataset, another case with *IL22*, and ecDNAs with the cytokines *CCL24* and *CCL26*, although these events were not found recurrently among different samples. Taken together, these results demonstrate the diversity of ecDNA-amplified oncogenes in GBM. Overwhelmingly, ecDNA is the primary mode of oncogene amplification in this tumor type.

The GBM ecDNA landscape reveals a distinctive heuristic pattern (Fig. 2A). *EGFR*-containing ecDNAs do not coamplify any other known oncogenes on the same ecDNA species. In contrast, ecDNAs containing *PDGFRA* or *MDM2* frequently coamplify other oncogenes. In the case of *PDGFRA*, we also find *KIT*, *KDR*, *CHIC2*, *FIP1L1*, *PIK3C2B*, and/or *CLOCK* on the same circular ecDNA. The native loci of these oncogenes all derive from chromosome 4. Similarly, ecDNAs containing *MDM2* also harbor oncogenes *CDK4* and *AGAP2* on the same ecDNA in all cases, as well as *DDIT3* in one case, which are all genes located on chromosome 12. Consistent with previous observations (25), ecDNA-amplified oncogenes can reach extremely high median copy numbers (>100) in individual tumors (Fig. 2C) that is not seen for intrachromosomal amplifications (median ecDNA oncogene copy number = 53, median intrachromosomal oncogene copy number = 18; Mann–Whitney *U P =* 0.002; Fig. 2D). The mean copy number of ecDNA-amplified oncogenes is higher than that of non-oncogenes (Mann–Whitney *U P =* 0.006; Fig. 2E), and ecDNAs are structurally more complex compared with intrachromosomal focal amplifications (mean complexity scores, derived using AmpliconArchitect, of 0.84 and 0.43 for ecDNA and linear amplifications, respectively; Mann–Whitney *U P* < 0.0001; Fig. 2F). Most ecDNAs contain DNA from a single chromosome; however, we find ecDNAs derived from two chromosomes (translocation–ecDNA) in 12% (*n* = 6/49) of GB-UK samples and 14% (*n* = 5/35) of PCAWG samples (Fig. 2G). We did not detect combinations of three or more chromosomes on the same ecDNA.

Interestingly, ecDNA sizes are conserved within one order of magnitude in both GB-UK and PCAWG (minimum = 180 kbp; maximum = 5.52 Mbp; Fig. 2H). We furthermore observe a negative correlation between the number of ecDNA-amplified genes and ecDNA copy number (Spearman r=-0.5, P=0.001 and r=-0.5, $P=7.5\times 10^{-5}$ for GB-UK and PCAWG, respectively; Fig. 2B). We see the same strong negative correlation of median *EGFR* copy number and the size of intergenic regions on ecDNA, suggesting interpatient variation in ecDNA composition and possible selection against nonessential DNA amplified on ecDNAs (GB-UK and PCAWG combined, Spearman r=-0.6; P=0.002; Fig. 2I). This is further supported by ecDNA structure. For example, all ecDNAs in GB-UK (*n* = 3/3) and PCAWG (*n* = 4/4) containing *CDK4* and *MDM2* lost the intervening chromosomal segment between both genes. Based on our data, we cannot distinguish if this particular structure emerged from the loss of the intergenic region or the ligation of two separate fragments. Nevertheless, this pattern of convergent structural evolution suggests an evolutionary benefit of a smaller structure, the precise nature of which currently remains unknown.

### Spatial ecDNA Copy-number Heterogeneity

**Figure 3.**
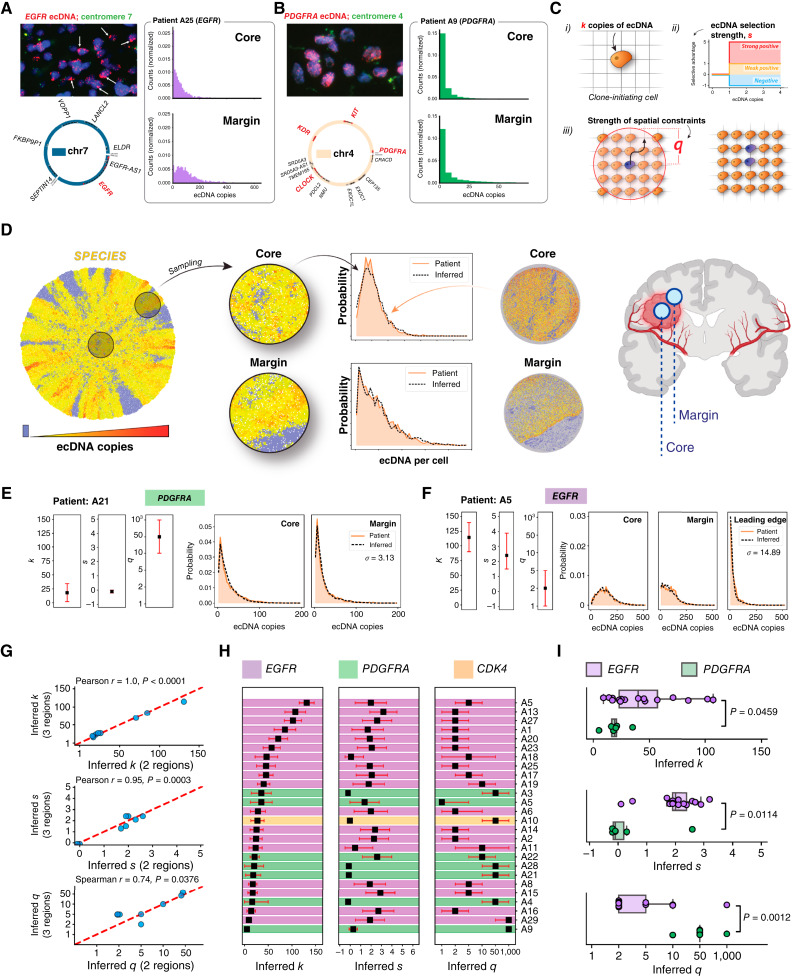
SPECIES spatial modeling of ecDNA evolution. **A** and **B,** DNA FISH staining of two representative GBM samples from the GB-UK cohort, revealing (**A**) *EGFR*- and (**B**) *PDGFRA*-ecDNA. *EGFR*-ecDNA seem to congregate into “hubs” (white arrows). Single-cell ecDNA copy-number distributions are derived from DNA FISH images using unbiased image analysis. **C,** (i) SPECIES is initiated with a single tumor cell containing *k* copies of ecDNA. (ii) Model of ecDNA selection. Cells carrying one or more copies of ecDNA divide at a rate *s* faster than cells lacking ecDNA. (iii) Cells push neighbors within a radius *q* on the lattice to make space required for cell division. **D,** Spatial computational model of ecDNA-driven tumors. ABC was used to infer optimal model parameters for the initial number of ecDNA, *k*; ecDNA-conferred selection, *s*; and cell pushing strength, *q*, for a given set of patient tumor measurements. **E** and **F,** Example output of the ABC model fitting algorithm using (**E**) 2 and (**F**) 3 spatial regions. (Left to right) Inferred *k*, *s*, and *q* and model best-fit distributions for each tumor region. The sum of the Wasserstein distance between patient and simulated distributions for each tumor region, representing closeness of fit, is denoted by σ. **G,** Comparison of inferred *k*, *s*, and *q* when analyzing 2 (core and margin) vs. 3 (core, margin, and leading edge) spatial regions. **H,** Summary of inferred model parameters for all sampled human GBM tumors. The highlighted color represents ecDNA-amplified oncogenes (*EGFR*, *PDGFRA*, or *CDK4*) in each tumor. **I,** Inferred model parameters for all patient tumors (excluding the tumor of patient A5, which contained separate *EGFR-* and *PDGFRA*-ecDNA species), stratified by ecDNA-amplified oncogene (*EGFR* or *PDGFRA*). (**D,** created using BioRender assets. https://BioRender.com/7f2pume)

Although *SEC61G*, a gene promoting immune evasion (26), coamplifies on the same ecDNA with *EGFR*, no other known oncogenes are in the proximity of *EGFR* that could be coamplified on ecDNA and drive their high observed copy numbers. The frequent convergent evolution of extremely high copy number ecDNA-amplifying *EGFR* in GBM thus suggests an important role in tumor evolution and that *EGFR-*ecDNA and its variants are under strong selective pressure. If this were true, we would anticipate different patterns of spatial temporal evolution of *EGFR*-containing ecDNAs relative to ecDNAs that contain other oncogenes. To test this hypothesis, we measured the spatial variation of the two most common ecDNA-amplified oncogenes, *EGFR* and *PDGFRA*, with DNA FISH in the core and infiltrating margin in each GBM in the GB-UK cohort (Fig. 3A and B). The DNA FISH- and WGS-derived mean ecDNA copy numbers strongly correlate (Pearson r=0.6, P=0.01; Supplementary Fig. S4). Unbiased image analysis (“Methods”) then allowed us to quantify the per-cell ecDNA count for each core and margin region separately. Both *EGFR-* and *PDGFRA*-ecDNAs, when observed in tumor cores, are frequently also seen in margins and leading edges (72%, *n* = 23/32; Supplementary Fig. S5). ecDNA copy numbers show the typical wide cell-to-cell variation that emerges from random ecDNA segregation during cell division (Supplementary Fig. 6; refs. 15, 16). *EGFR*-ecDNA single-cell copy-number distributions, however, are highly variable across different regions of a patient’s tumor, whereas *PDGFRA*-ecDNA copy-number distributions are more similar between core and margin samples of the same tumor (measured with Wasserstein distance, Mann–Whitney *U P* = 0.0059, Supplementary Fig. S7A and S7B). This difference is also reflected in the variation of mean ecDNA copy numbers in core and margin samples across patient tumors, consistent with a spatially heterogeneous ecDNA pattern, particularly marked among tumors harboring *EGFR*-ecDNA (Supplementary Fig. S7C and S7D).

We then tested how such differences might emerge in the spatial-temporal evolution of ecDNA-positive tumors. We developed stochastic computer simulations of expanding ecDNA-driven tumor populations (SPECIES), adapting lattice-based cellular automaton models for the acquisition of chromosomal genetic heterogeneity (“Methods”; Fig. 3C; refs. 27, 28). SPECIES requires three input parameters: the ecDNA copy number, *k*, in the first tumor-initiating cell; the strength of the ecDNA-conferred selective advantage, *s*; and the range of spatial constraints, *q* (Fig. 3C). For any set of these parameters, SPECIES generates explicit spatial and temporal patterns of single-cell ecDNA copy numbers, enabling us to mimic *in vivo* multiregion sampling (Fig. 3D). In the limit of weak spatial constraints (*q* > 10), SPECIES results converge on previously published simulations of well-mixed populations by Lange and colleagues (15), validating the SPECIES computational approach (Supplementary Fig. S8A–S8C).

Different regimes of the parameters *k*, *s*, and *q* result in dramatically different spatial patterns of simulated ecDNA cell-to-cell variation (Supplementary Fig. S9A–S9D). The effect of the spatial constraint parameter, *q*, on the measured ecDNA copy-number distributions is highly nonlinear; large *q* values (e.g., 10, 50, and 1,000) lead to weak spatial constraints and produce more spatially homogeneous tumors, whereas small *q* values give rise to local spatial sectors and greater spatial variation in ecDNA copy number. This is reflected within each subsampled spatial region, with an increase in *q* leading to greater similarity between core and margin ecDNA copy-number distributions, on average (Supplementary Fig. S7D). Despite *s* having a weaker impact on the ecDNA copy-number distribution when paired with large *k* (due to our chosen constant selection model; see “Methods”), larger *s* nevertheless drives the tumor population to higher ecDNA copy numbers, through ecDNA-positive cells outcompeting ecDNA-free cells. Simulated spatial ecDNA patterns are most sensitive to the value of selection strength, *s*, when combined with low *k* as tumors initiated with few ecDNA copies (i.e., low *k*) rapidly generate less fit ecDNA-free cell lineages as a result of random ecDNA segregation. Due to the inherent stochasticity of cell divisions and random ecDNA segregation, however, individual realizations of simulations vary in the exact spatial patterns for any fixed set of these evolutionary parameters. Spatial dynamics coupled with sampling can further cause nonintuitive effects. For example, spatial simulations suggest the mean ecDNA copy number can both increase and decrease from core to margin samples despite strong positive selection (s≥1). We see similar variation in patient data with more pronounced effects for ecDNA-amplified *EGFR* compared with *PDGFRA* in line with the overall higher *EGFR-*ecDNA copy number (Supplementary Fig. S7A and S7B).

### Computational Inferences of ecDNA Evolutionary Properties

To quantitatively estimate which parameter combinations best describe growth dynamics in individual patient tumors, we implemented approximate Bayesian computation (ABC; refs. 29, 30) to fit SPECIES simulations to patient-derived ecDNA copy-number distributions (“Methods”; Fig. 3E; Supplementary Figs. S10–S16). SPECIES accurately recapitulates ecDNA copy-number distributions in core and margin samples for each patient tumor (Wasserstein distance metric σ<10, see “Methods”). The best inferred model parameters differ between patients. We find a wide range of ecDNA copy numbers, *k*, in the first tumor-initiating cell, ranging from $5_{-4}^{+5}$ (patient A9, *PDGFRA*) to $131_{-16}^{+16}$ (patient A5, *EGFR*). In 15 out of 26 tumors, ecDNA copy-number distributions suggest a model *k* value of 20 or more. In these tumors, ecDNA likely accumulated some time prior to the most recent tumor clonal expansions. Evidence for the accumulation of ecDNA in premalignant human populations recently emerged in longitudinal studies of cancerous transformations in Barrett’s esophagus (5). Due to a plateau in the effect of differential fitness at high ecDNA clonality (i.e., as a result of the constant selection model, if all or most cells contain ecDNA, then their fitness will, in effect, be neutral), inferring the exact value of *s* in strong fitness regimes can be difficult, as exemplified by wide posterior parameter distributions for some patients’ tumors. Nevertheless, in 18 out of 26 tumors, we estimate strong selective advantages for ecDNA-positive cells (s≥1; 16/18 *EGFR*; 2/7 *PDGFRA*; 0/1 *CDK4*). Eight samples are consistent with the neutral dynamics of ecDNA-positive cells (error regions on inferred *s* values for these tumors include *s =* 0) though the high abundance of ecDNA observed in these eight GBMs further suggests a positive ecDNA-conferred fitness advantage (15). In most cases, SPECIES inferences suggest strong spatial constraints, corresponding to small *q* (q=2 and q=5)*,* implying that tumor cells are constrained for space during tumor expansion but are not strictly confined to boundary growth (q=1). Some patients’ tumors were predicted to have moderate-to-weak spatial constraints (q=10 or 50), possibly driven in part by the similarity of the ecDNA copy-number distribution across the tumor core and margin; however, further clinical and experimental validation is warranted to confirm these computational inferences.

To ensure our inferences account for extensive intratumoral spatial heterogeneity, we then measured ecDNA copy-number distributions for three regions per tumor (core, margin, and leading edge) for a subset of the GB-UK cohort (*n* = 3 *EGFR* and *n* = 3 *PDGFRA*). ABC inferences for *k*, *s*, and *q* using two or three tumor regions are highly correlated (Fig. 3F and G). Another concern may be the scaling of simulated tumor sizes and ABC parameter inferences. ABC inferences are computationally costly (here, we used $3.2\times 10^{6}$ simulated tumors per patient tumor), limiting the achievable sizes of simulated tumors. We tested that limitation and show inferences of simulated tumors of 10^6^ or 10^7^ cells strongly correlate (Supplementary Fig. S17). Although precise parameter estimates may weakly depend on population size, overall trends are robust to such variations.

Alongside the single-cell copy-number distributions, the fraction of ecDNA-positive cells in the tumor contains important information on selective forces. Our parameter fitting approach also utilizes this information to fit the simulation to patient data (Supplementary Methods), which might drive the consistent inference of weak selection for *PDGFRA*-ecDNA tumors. Furthermore, it is indeed possible that neutral or negatively selected ecDNAs emerge in the tumor; however, a large fraction of these will be lost over the course of expansion (Supplementary Fig. S18), leading to a depletion of such cases in our dataset. Thus, the presence of ecDNA in the patients’ tumors itself suggests that these ecDNA species are under positive selection—a notion that is further supported by our Bayesian inferences using the measured ecDNA copy-number distributions.

It is worth noting that SPECIES is a minimal implementation of spatial ecDNA evolution. Possible biological complexities not accounted for in SPECIES—such as ecDNA copy number–dependent selection, interactions with the tumor microenvironment, additional chromosomal genomic alterations, or the three-dimensional tissue architecture—may each further affect spatial patterns of ecDNA in a tumor. Our modeling approach assumes that all parameters remain constant over time, which is not likely, as the selective advantage of ecDNAs may change over time. Future work will be needed to better understand the dynamic evolutionary pressures as tumors progress over time, which may yield a deeper understanding of glioma biology and potentially lead to more effective treatment strategies. Nevertheless, a minimal model with only three free parameters quantitatively recovers much of the diversity of ecDNA copy-number patterns observed *in vivo*, both spanning spatial regions within individual tumors and across patient tumors. In summary, temporal spatial simulations of ecDNAs in growing tumors, compared with core and margin samples, suggest that the majority of oncogene-containing ecDNAs are important cancer drivers that were likely present at the onset of the most recent clonal expansion in ecDNA-positive human GBMs.

### ecDNA Oncogene-Specific GBM Evolution

We then asked whether GBM evolutionary dynamics depend on ecDNA cargo. In GB-UK, DNA FISH–derived copy numbers of *EGFR-* versus *PDGFRA*-ecDNA had significantly different distributions for both maximum and mean copy number, cell-to-cell variance, and skew (Supplementary Fig. S19, Mann–Whitney *U*$P=6.4\times 10^{-4\;}$, $P=2\times 10^{-5}$, $P=2.7\times 10^{-4}$, and $P=4.8\times 10^{-4}$, respectively). *PDGFRA*-ecDNAs were larger than those amplifying *EGFR* (Mann–Whitney *U*P=0.0074) or *CDK4* (Mann–Whitney *U*P=0.019); however, all ecDNAs had similar structural complexity (AmpliconArchitect complexity score) regardless of the amplified oncogene. In keeping with these differences, SPECIES predicts a wider range of inferred *k* and *s* parameters among tumors with *EGFR*-ecDNA compared with *PDGFRA-*ecDNA (IQR of *k* was 34 and 4.25 for *EGFR* and *PDGFRA*, respectively; IQR of *s* was 0.5 and 0.375 for *EGFR* and *PDGFRA*, respectively, Fig. 3H). Although stochasticity is an inherent feature of the ABC parameter inference algorithm, this heterogeneity likely reflects differences in the underlying biology between patients’ tumors. Indeed, we observe different evolutionary dynamics between *EGFR*- and *PDGFRA-*ecDNA amplifications. Tumors containing ecDNA-amplified *EGFR* show significantly larger predicted initial ecDNA copy number, *k*; selective strength, *s*; and stronger spatial constraints, that is, smaller *q* (Mann–Whitney *U*P=0.0459, P=0.0114, and P=0.0012, respectively) compared with tumors with ecDNA-amplified *PDGFRA* (Fig. 3I). These predicted oncogene-level differences are independent of variations in the spatial location of the core sample (to reflect similar variances from the patient tissue acquisition), different cell birth/death ratios, and simulated tumor size (Supplementary Methods; Supplementary Figs. S17 and S20). Furthermore, model fit accuracy declines noticeably when restricting initial simulated ecDNA copy numbers to *k* = 1 (Supplementary Methods). This decline is most pronounced in patient samples with the highest predicted *k* values, further suggesting that in some patient tumors, the assumption of a high initial ecDNA copy number *k* is required to capture observed shifts in ecDNA distributions between core and margin samples (Supplementary Fig. S21A–S21C).

### ecDNA Accumulation and Selection in Genetically Engineered Mice

**Figure 4.**
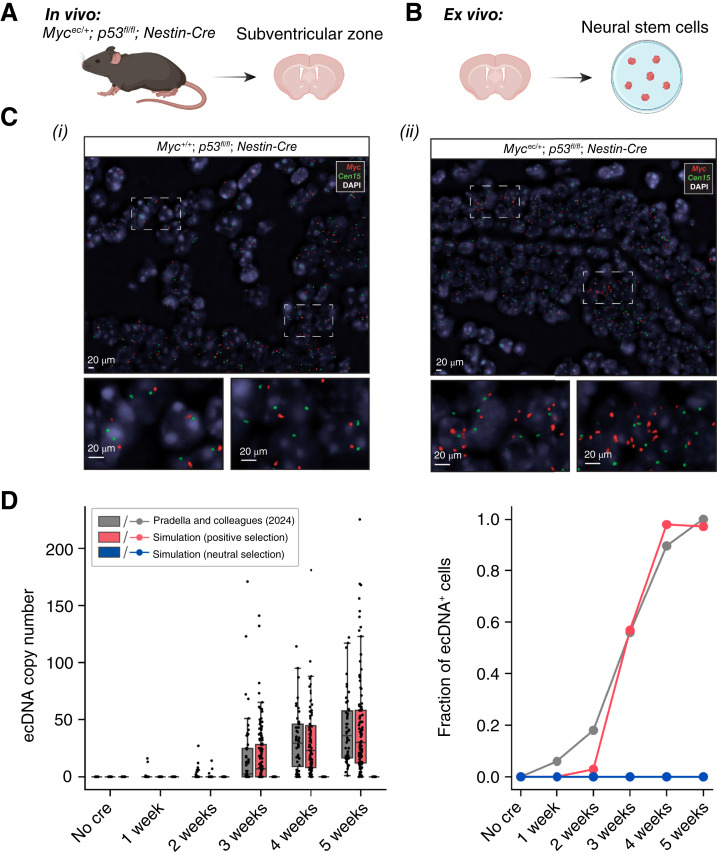
ecDNA accumulation in genetically engineered *in vivo* and *ex vivo* murine models. **A,** Neural stem cells were propagated within the SVZ of genetically engineered mice for 4 months. **B,***Myc***-**ecDNAs were induced in adult murine neural stem cells, which were then cultured *ex vivo* for 5 weeks. **C,** DNA FISH analysis of neural stem cells within the SVZ of genetically engineered mice (i) without *Myc*-ecDNA (*Myc*^*+/+*^*; P53*^*fl/fl*^*; Nestin-Cre*, *n* = 2) and (ii) with *Myc*-ecDNA (*Myc*^*ec/+*^*; p53*^*fl/fl*^*; Nestin-Cre*, *n* = 2), both on a background of homozygous *Trp53* loss. **D,** ecDNA copy-number dynamics in murine adult neural stem cells (data from ref. 33) with corresponding simulated dynamics, assuming either neutral or positively selected ecDNA. (**A** and **B,** created using BioRender assets. https://BioRender.com/a1hpt9n.)

A recent study in mice provided support for a precancerous state in GBM derived from neural stem cells in the subventricular zone (SVZ) although the authors did not investigate the possible implications of ecDNA (31). To experimentally confirm our predictions from SPECIES of both precancerous ecDNA accumulation and strong ecDNA selection *in vivo*, we analyzed neural stem cells in the SVZ of genetically engineered mice with ecDNA amplifying the *Myc* oncogene on a background of homozygous *Trp53* loss (*Myc*^*ec/+*^*; p53*^*fl/fl*^*; Nestin-Cre*, *n* = 2; Fig. 4A and B). *Myc* signals downstream of *EGFR* and has previously been shown to be oncogenic in GBM (32). DNA FISH analysis of the SVZ of these mice at 4 months old showed the presence of multiple copies of *Myc*-ecDNA in a subset of SVZ cells, without a histologically detectable brain tumor at this stage. In contrast, *Myc*-ecDNA was absent in the SVZ of mice with only *Trp53* loss (*Myc*^*+/+*^*; p53*^*fl/fl*^*; Nestin-Cre*, *n* = 2) at the same time point (Fig. 4C). Moreover, model simulations of data showing *Myc*-ecDNA accumulation in primary adult neural stem cells from *Myc*^*ec/+*^*; p53*^*fl/fl*^ mice [infected with Ad-Cre *in vitro* (33)] were consistent with positive selection of *Myc*-ecDNA *in vitro* with *s* = 2, implying an approximate 200% increase in the fitness level of neural stem cells with *Myc*-ecDNA compared with those without (Fig. 4D; Supplementary Methods). Model simulations in which *Myc*-ecDNA is under neutral selection show that there is invariably early loss of these ecDNAs. These results further support the predictions from SPECIES that in a subset of human GBMs, oncogenic ecDNA may accumulate in neural precancerous cells prior to clonal expansion.

### *EGFR-*ecDNA–Variant Heteroplasmy in GBM

**Figure 5.**
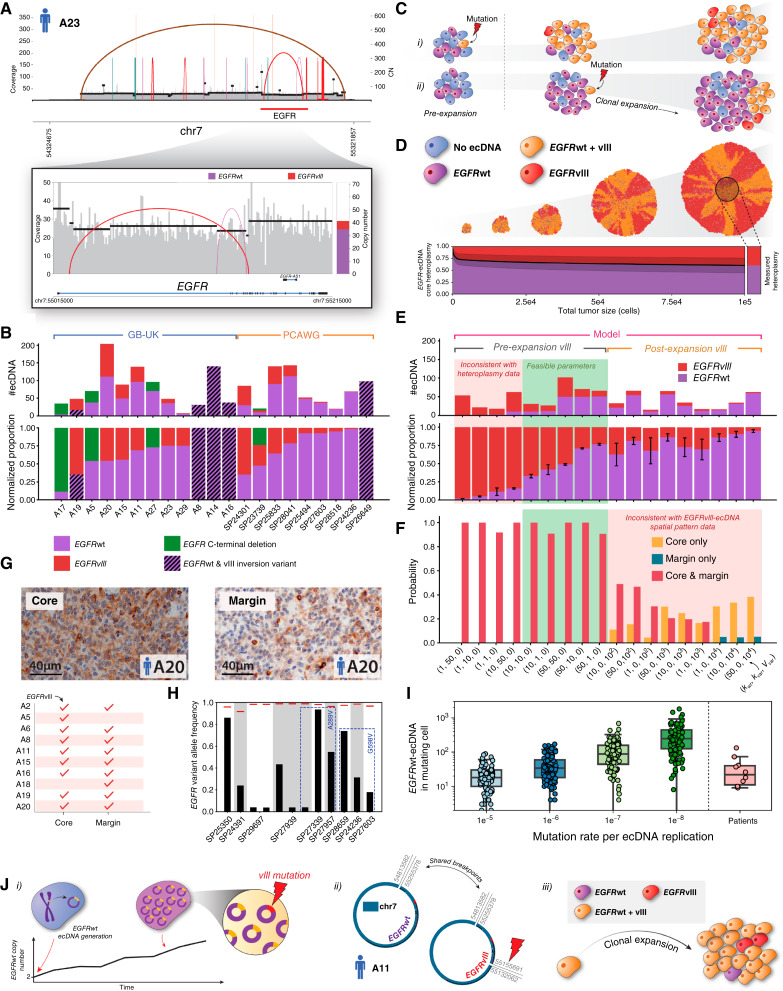
Selection dynamics of *EGFR* SVs on ecDNA. **A,** A subclonal *EGFRvIII* variant on ecDNA in the tumor of patient A23. **B,** Variant heteroplasmy in the core region of all samples across the GB-UK and PCAWG cohorts, which contained *EGFR* variant–bearing ecDNA. **C,** Effects of (i) pre- and (ii) postclonal expansion mutation of *EGFR*-ecDNA on its resulting spatial pattern in the expanded tumor. **D,** SPECIES was applied to simulate the emergence of an advantageous *EGFRvIII* mutation on ecDNA, either prior to or after the onset of clonal expansion of the tumor. The example shown is for a pre-expansion mutation, with parameters ($k_{wt}=20$, $k_{var}=1$, $V_{var}=0$). The SD of ecDNA heteroplasmy is shown by the shaded region in the lower plot. **E,** Model predictions for resulting variant heteroplasmy in a tumor for a range of wild type- and variant-bearing ecDNA copy numbers in the clone-initiating tumor cell (for pre-expansion *EGFR* mutation) and mutation times (for post-expansion *EGFR* mutation). The red-shaded region denotes parameter combinations inconsistent with observed patient core heteroplasmy values. Mean ± variance is derived from 1,000 simulated tumors. **F,** SPECIES model predictions for the probability of observing *EGFRvIII* ecDNA across tumor core and margin, for a pair of core and random margin regions. Mean values are derived from 1,000 simulated tumors. **G,** (Top) IHC slide for the tumor of patient A20, showing the presence of *EGFRvIII* in the tumor core and infiltrating margin (blue = hematoxylin; brown = *EGFRvIII*). (Bottom) Summary of *EGFRvIII* in core and margins of GB-UK cohort samples, determined by IHC. **H,** Variant allele frequencies of point mutations within *EGFR* in PCAWG samples harboring *EGFR*-ecDNA. Red lines represent the frequency expected for a mutation carried by all ecDNAs within a sample. **I,** Distributions of *EGFR*wt-ecDNA abundance in the cell that acquires the first mutation, for a range of ecDNA mutation rates. Patient tumor values are derived from their respective inferred *k* values, assuming that the arrival of a mutation on ecDNA initiates clonal expansion, implying that the number of *EGFR*wt-ecDNA in the mutating cell is thus equal to *k* − 1 (with the remaining one ecDNA carrying the mutated allele). **J,** Timeline of *EGFR*-ecDNA tumorigenesis. (*i*) Initial *EGFR*wt-ecDNA is generated and accumulates in precancerous cells, conferring a moderately positive selective advantage. In time, the *EGFRvIII* mutation occurs on one of the accumulated *EGFR*wt-ecDNAs, conferring a strong positive advantage. (*ii*) Reconstructed ecDNA structures from the tumor of GB-UK patient A5 confirm that *EGFR*wt and *EGFRvIII* ecDNAs share common breakpoints, indicating that *EGFRvIII* arose from the mutation of an existing *EGFR*wt-ecDNA. (iii) *EGFRvIII*-ecDNA further promotes clonal expansion, driving tumor growth.

What then determines the transition of precancerous *EGFR*-ecDNA accumulation into clonal expansions? A two-hit model of ecDNA formation followed by a secondary event (e.g., an ecDNA mutation), increasing its fitness and initiating clonal expansion, could potentially explain high initial *EGFR-*ecDNA copy numbers. To investigate evidence for a second hit, we analyzed the SVs on *EGFR*-ecDNA and its variation within the core samples of single tumors (corresponding WGS data for tumor margins were unavailable; Supplementary Fig. S22). In GB-UK, 67% (*n* = 12/18) of patient tumors with ecDNA-amplified *EGFR* carried mutated *EGFR* variants on ecDNA, with *n* = 6/18 harboring the GBM-specific oncogenic *EGFRvIII* variant [constitutively active *EGFR* (34), Fig. 5A]. *EGFRvIII* was exclusively amplified on ecDNA. Surprisingly, in all *EGFR*-ecDNA positive tumors, *EGFRvIII* coexisted with *EGFR* wild-type (*EGFR*wt) ecDNA, usually at lower copy numbers compared with *EGFR*wt amplifications (Fig. 5B). Within each sample, the *EGFR*wt and *EGFRvIII* ecDNA shared the same genomic breakpoints, suggesting that the *EGFRvIII* ecDNA variants arise from existing *EGFR*wt ecDNAs. A similar dynamic has been previously suggested to explain *EGFRvIII* acquisition in the GBM39 cell line using CRISPR-CATCH (35). By contrast, a model in which the two ecDNA species formed independently from separate alleles is highly unlikely, as this would require that both ecDNA formation breakpoints occur at precisely the same genomic location. Furthermore, a model of biased ecDNA formation breakpoints, possibly due to the existence of fragile regions of the genome, would predict conservation of ecDNA formation breakpoints when comparing ecDNA across tumors. Such an effect is not observed (23), however, suggesting that ecDNA formation is unbiased at the base pair level, further supporting the notion that the *EGFR*wt and *EGFRvIII* ecDNA species observed in some GB-UK patient tumors can be traced back to the same singular ecDNA formation event.

In addition, three GBMs carried ecDNAs with an *EGFR* C-terminal deletion (exons 25–27) and an activating *EGFR* truncation (36, 37) not previously reported to occur on ecDNA (Supplementary Fig. S23A and S23B). Four GBMs had an inversion SV between exons 1 and 7, which codes for the *EGFR* extracellular binding domain and is likely to lead to a nontranscribed segment with functional equivalence to *EGFRvIII* (Supplementary Fig. S22). This heteroplasmy-like variation of *EGFR* SVs (i.e., their co-occurrence with *EGFR*wt) is likely enabled by the wide cell-to-cell ecDNA copy-number heterogeneity and forms through the interplay of random segregation, selection, and mutation of ecDNAs. The observation of ecDNA heteroplasmy, however, does not directly indicate whether the mutating event occurs before or after the initial expansion of the tumor.

### Early Emergence and Selection of ecDNA-Amplified *EGFR* Variants

To better understand the dynamics of activating *EGFR* mutations on ecDNA, we adapted SPECIES to simulate expansions of advantageous ecDNA variants in the background of preexisting *EGFR*wt-ecDNA (Fig. 5C and D). This allows us to study how the initial copy numbers of wild-type and mutated *EGFR*-ecDNA, $k_{wt}$ and $k_{var}$ respectively, and the time of mutant ecDNA emergence, $V_{var}$, affect ecDNA heteroplasmy (defined here as the percentage of ecDNA copies that are amplifying *EGFR*wt; see “Methods”). A direct Bayesian inference of these parameters using SPECIES simulations requires resolved copy-number distributions of wild-type and variant ecDNAs in core and margin samples, which currently are not available. We, therefore, employed a different strategy to exclude certain evolutionary scenarios based on available data. The copy numbers of wild-type and variant ecDNAs and the spatial spread of ecDNA heteroplasmy in tumors contain orthogonal information on the timing and selection of *EGFR* mutations on ecDNA (Fig. 5E and F). We show with simulations that this information excludes a wide range of possible scenarios.

First, simulated and patient *EGFR*wt- and *EGFRvIII*-ecDNA copy numbers are consistent with the emergence of an activating variant (*vIII* or C-terminal deletion) at, or close to, the onset of clonal expansion (Fig. 5B and E). Simulations with either a large initial abundance of *EGFR*wt-ecDNA ($k_{wt}/k_{var}$ ratio of 10:1, 50:1, or 50:10) or both ecDNA types ($k_{wt}/k_{var}$ ratio of 10:10 or 50:50) are compatible with patient data. Four patients’ tumors from the PCAWG cohort harbored a particularly high ecDNA heteroplasmy, which may indicate that *EGFRvIII*-ecDNA was not present at the onset of clonal expansion but occurred in its early stages (${0<V}_{var}\leq 10^{4}$ cells). Simulations of tumors derived from a cell with low *EGFR*wt-ecDNA copy number [$k_{wt}/k_{var}$ ratio of 1:1, 1:10, 1:50, or 10:50; Fig. 3E (red-shaded region)] did not match qualitatively with observed copy numbers in patient samples. Despite recent evidence for cosegregation of multiple ecDNA species in tumors (38), SPECIES simulations suggest that if present, such dynamics between *EGFR*wt- and *EGFRvIII-*ecDNA have only a minor effect on the measured ecDNA heteroplasmy (Supplementary Fig. S24).

To further narrow the timing of *EGFR*-ecDNA variants, we measured the spatial extent of the *EGFRvIII*-ecDNAs in the GB-UK cohort using IHC (“Methods”; Fig. 5G). *EGFRvIII*-ecDNAs are typically maintained across the core and infiltrating margin of tumors (*n* = 8/10), which, according to spatial simulations, strongly suggests that *EGFRvIII* ecDNA variants emerge prior to tumor clonal expansion (Fig. 5G). SPECIES simulations with later arising *EGFRvIII* ecDNAs predict spatially variegated patterns of *EGFR*wt and *vIII* ecDNA, with large regions of the tumor margin only harboring one but not both types [Fig. 5F (red-shaded region)]. Such variegated patterns are not observed in patient data.

We next asked if there is evidence for activating mutations in *EGFR*-ecDNAs other than vIII and the inversion SV between exons 1 and 7. We, therefore, further analyzed the PCAWG GBMs (Fig. 5H) and found point mutations in *EGFR*-ecDNA in nine GBM samples. All point mutations were ecDNA subclonal, implying they likely occurred after ecDNA formation, yet some were observed at high variant allele frequencies, suggesting that they may have occurred on an ecDNA prior to clonal expansion (Fig. 5H; Supplementary Fig. S25). Two distinct mutations (*p*.A289V and *p*.G598V) on *EGFR*-ecDNA each recur in three patient GBMs, again suggesting selection of these variant ecDNAs. Interestingly, both mutations affect the *EGFR* extracellular domain, and *EGFR p*.A289V has been previously shown to correlate with poorer prognosis as well as a more invasive GBM phenotype in humans and mice (39).

To further test if *EGFR*-ecDNA variants show signs of positive selection, we applied dN/dS (using genesetdnds from the package dNdScv) to truncating mutations in ecDNA-amplified *EGFR* and *PDGFRA* detected previously in GBMs from the Genomics England cohort, following the method of Bailey and colleagues (22). dN/dS scores suggest positive selection for *EGFR*-ecDNA variants, whereas no such signal was observed for *PDGFRA*-ecDNA variants (7.00 vs. 0.00). Simulations of *EGFR*wt-ecDNA dynamics in precancerous tissue suggest that an *EGFR*-activating mutation on ecDNA would typically arise in a cell with a large abundance of *EGFR*wt-ecDNA (>10 copies), giving rise to a highly variable initial heteroplasmy often at 90% or above (Fig. 5I).

Taken together, this suggests the following typical scenario of ecDNA-amplified *EGFR* in human GBM: *EGFR*wt-ecDNA forms in precancerous tissue, conferring a moderate selective advantage [Fig. 5J (i)]. In time, *EGFR*wt-ecDNA copy numbers increase, further increasing the likelihood of acquiring a strongly advantageous activating mutation on *EGFR*wt-ecDNA [*vIII*, a C-terminal deletion, or a driver point mutation; Fig. 5J (ii)]. During clonal expansion, *EGFR*wt-ecDNAs hitchhike on strongly selected *EGFRvIII-*ecDNA, maintaining intratumor *EGFR* heteroplasmy despite a strong selection advantage of *vIII* variants [Fig. 5J (iii)]. This would be consistent with previous reports of coexpression of *EGFR*wt and *EGFRvIII* in primary human GBM (40). This heteroplasmic ecDNA mixture, coupled with the wide ecDNA copy-number distribution, has been shown previously in cell lines to allow both rapid resistance evolution (erlotinib treatment) and high resilience to hostile growth conditions (glucose withdrawal; ref. 15), and may underlie the negative results in previous clinical trials of *EGFR* inhibitors. Moreover, multiple SVs consistent with *vIII* are observed in sample A20 from GB-UK and SP24301, SP23739, and SP28041 from PCAWG, suggesting ongoing convergent evolution of activating *EGFR* mutations within these ecDNAs (Supplementary Fig. S22).

Overall, early emergence and strong selection of *EGFR-*ecDNA variants is consistent with all our patient samples (in which variants are present) except for two. The tumor of patient A5 harbored *EGFRvIII*-ecDNA only in the tumor core. This may be either a sampling artifact or an *EGFR*wt-ecDNA that may have mutated not before but shortly after the onset of clonal expansion (Fig. 5G). Similarly, the tumor of patient A18 harbored *EGFRvIII*-ecDNA only in the infiltrating margin, perhaps suggesting the *vIII* mutation developed on ecDNA late into clonal expansion.

### Coselection and Cosegregation Dynamics of Multiple ecDNA Species

**Figure 6.**
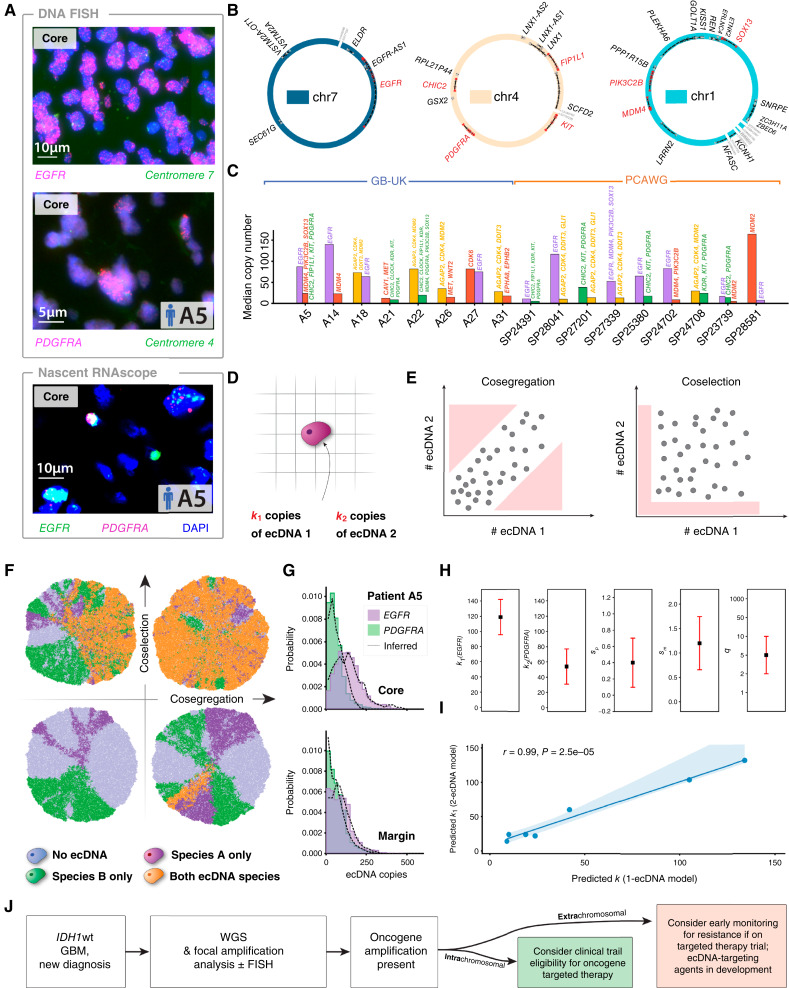
Simulating multi-ecDNA dynamics *in vivo.***A,** DNA FISH and nascent RNAscope images from the tumor of patient A5, showing the coexistence of both *EGFR*-ecDNA and *PDGFRA*-ecDNA. **B,** Circular structures of three distinct oncogenic ecDNAs detected in the tumor of patient A5. **C,** GBMs in the GB-UK and PCAWG cohorts containing more than one oncogenic species and their respective copy numbers. **D,** In a multiple-ecDNA application of SPECIES, the clone-initiating cell begins with $k_{1}$ and $k_{2}$ copies of ecDNA 1 and ecDNA 2, respectively. **E,** Representation of the impacts of ecDNA cosegregation and coselection on the number of inherited ecDNA copies during cell division. Cosegregation drives correlated inheritance of both ecDNA types, whereas coselection favors tumor cells carrying at least one copy of each type. **F,** Application of SPECIES to simulate two separate ecDNA species. Simulated tumors were initialized with a single cell, carrying $k_{1}$ and $k_{2}$ copies of each ecDNA species. Representative model images show examples of low and high cosegregation and coselection. **G** and **H,** Parameter inference summary for the tumor of patient A5, for which we measured both the *EGFR*-ecDNA and *PDGFRA*-ecDNA copy-number distributions using DNA FISH, using multiple-ecDNA SPECIES. Parameters $s_{p}$ and $s_{m}$ represent selection coefficients for tumor cells with 1 (pure) or 2 (mixed) ecDNA species, respectively. **I,** Comparison of inferred *k* and $k_{1}$ values for all patient tumors confirmed by WGS to harbor two or more ecDNA species. **J,** The presence of ecDNA amplifications may be used to aid stratification, given that oncogenes on ecDNA have an inherent resistance mechanism through the ability of ecDNA to dynamically adjust copy number in response to targeted agents. Earlier monitoring and intervention are recommended for those patient tumors with ecDNA that will receive targeted therapies.

Recently, the distinct nature of coamplification and coselection of multiple ecDNA species in cancers has been discovered (38). In this study, 16% (*n =* 8/49) of GB-UK GBMs harbored at least two independent oncogenes amplified on different ecDNA. In one case (patient A5), the tumor contained three distinct ecDNA species, which together amplified seven oncogenes, including *EGFR*, *PDGFRA*, and *MDM4* (Fig. 6A and B). The tumor of patient A21 harbored one ecDNA containing *PDGFRA/KIT/KDR* and a second ecDNA with *MET* (copy number = 11 and 13, respectively). Among the GB-UK GBMs with multiple ecDNA species, *n* = 5/8 contained *MDM2* or *MDM4* amplified on one of the ecDNAs, implying that loss of the *TP53* pathway may facilitate ecDNA formation with multiple ecDNA species (Fig. 6C). Similarly, in PCAWG, we observed multiple ecDNAs per tumor in 29% (*n* = 10/35) of cases, with combinations such as *EGFR-*ecDNA with a *CDK4*-/*MDM2*-ecDNA. The data suggesting that disruption of *TP53* may be an important event in facilitating ecDNA formation are consistent with the recent observation in patients with Barrett’s esophagus who progressed to esophageal cancer, for which *TP53* loss always preceded ecDNA development (5). In patient tumors with multiple ecDNA oncogenic species, their nascent expression was frequently seen in different cells in a single tumor (Methods), suggesting that the presence of more than one ecDNA species can further increase intercellular oncogene expression heterogeneity.

SPECIES provides an opportunity to probe the dynamics of multiple ecDNA species in tumors. In the tumor of patient A5, DNA FISH revealed two separate ecDNA amplicons, amplifying *EGFR* and *PDGFRA* (Fig. 6A and B). When inferring tumor dynamics in this tumor, we did so both for the *EGFR* and *PDGFRA* separately, thus providing two sets of *k*, *s*, and *q* values. The best-fit values for *q* using data for either ecDNA amplicon in this tumor are similar, as expected, given that this parameter models the growth in the tumor and does not describe ecDNA-specific dynamics. Inferences on the *EGFR-*ecDNA copy-number distribution resulted in the largest predicted value of *k* (k=131±16) across the entire cohort. Furthermore, for this tumor, SPECIES predicted the second largest value of *k* and the lowest value of *q* across all *PDGFRA*-amplifying tumors (k=37±23; q=2), suggesting that the overall dynamics may be dominated by the *EGFR*-amplifying ecDNA.

The basic framework of SPECIES discussed above does not account, however, for emergent dynamics such as cosegregation (correlated inheritance of both ecDNA species) and coselection (selective advantage for maintaining a mixture of both ecDNA species within a cell) of multiple ecDNA species. These mechanisms may provide an alternative explanation for the inferred high abundance of preexisting ecDNAs in some tumors. To model the dynamics of multiple ecDNA species more faithfully, we adapted SPECIES to include two distinct ecDNA types and modeled cosegregation and coselection using the approach of Hung and colleagues (38) (Supplementary Methods; Fig. 6D and E). Depending on the strength of cosegregation and coselection of two ecDNAs, different scenarios for ecDNA maintenance across tumor space emerged (Fig. 6F). DNA FISH and nascent RNAscope analysis (the latter used to demonstrate single cells that express one or more ecDNA oncogenes) revealed that GB-UK GBMs harboring multiple ecDNA species, A5, A10, and A22, each maintained these populations in tandem across the core, infiltrating margin, and leading edge of the tumor, except for the infiltrating margin of A10 (Supplementary Fig. S5). These observations are consistent with coordinated inheritance and coselection of both ecDNA types (Fig. 6F). To further explore this scenario, we fit the multiple-ecDNA SPECIES model to the multiple-ecDNA GBMs in GB-UK for which we had single-cell ecDNA copy-number distributions (*n* = 7/8 patients). In each case, we compared the inferred initial copy number for one of the amplicons with that inferred with the single-ecDNA SPECIES model (Supplementary Methods). The inferred initial ecDNA numbers of *EGFR*- and *PDGFRA*-ecDNA in the tumor of patient A5 were highly consistent across the single- versus multiple-ecDNA SPECIES models (Fig. 6G and H; Supplementary Fig. S26). Due to the fitness plateau resulting from our constant ecDNA selection model, the value of *k* dominates the copy-number distributions in this tumor. Across all coamplified patient tumors in GB-UK, the value of *k* inferred with both SPECIES models was strongly correlated (Fig. 6I, Pearson r=0.99; $P=2.5\times 10^{-5}$). Thus, although coegregation and coselection dynamics likely play a role in these tumors to maintain high fractions of mixed cells, a high starting ecDNA copy number for one of the ecDNA species is still required to explain the observed spatial patterns of the ecDNA copy-number distribution.

## Discussion

In summary, our study reveals four critical insights about the role of ecDNA in *IDH*wt GBM. First, focal oncogene amplification almost always occurs on ecDNA. Copy-number distributions of ecDNA across spatially distinct regions vary between patients’ tumors and ecDNA cargo, suggesting that different oncogenic ecDNAs are under varying degrees of positive selection with spatial growth constraints and oncogene-specific ecDNA dynamics.

Second, it shows that *EGFR* and its variants are particularly potent, likely arising early in tumor development and likely providing a sufficient oncogenic stimulus to drive tumor formation and progression. Previous molecular analyses and mouse models have implicated *EGFR* amplification as an early or transformational event in some GBMs (41–44) though without consideration of ecDNA. In this study, we advance this work to quantitatively account for ecDNA spatial dynamics, revealing two groups of *EGFR* tumors—those with premalignant accumulation of ecDNA, in which ecDNA is likely to contribute to initiating clonal expansion, and those tumors with few ecDNAs in early tumorigenesis. The emergence of high-level *EGFR* amplification facilitates subsequent oncogenic mutations to arise on ecDNA, such as *EGFRvIII*, as well as point mutations such as *EGFR p*.A289V, which were not previously described on ecDNA although noted to be oncogenic (39). Further research with secondary and larger clinical cohorts will be essential to extend these findings.

Third, it reveals the ability of ecDNA to coamplify multiple weaker oncogenes to drive tumorigenesis, especially if they are located relatively close to each other in the chromosomal site of origin. Tumors with ecDNA-amplified *PDGFRA* coamplify other oncogenes on the same ecDNA and show different evolutionary trajectories compared with tumors with ecDNA-amplified *EGFR* and no other coamplified oncogenes.

Lastly, the development of our SPECIES model, based on both DNA, RNA, and spatial features, reveals that spatial–temporal evolutionary modeling can lead to unique insights into ecDNA-mediated oncogenesis. Intratumor heterogeneity of *EGFRvIII* contributes to the failure of *EGFR*-targeted therapies (45–47) although chimeric antigen receptor T cells targeting both *EGFRvIII* and *EGFRwt* have recently shown promise (48). The observation that amplification of wild-type *EGFR* on ecDNA, followed by mutational processes that give rise to more potent *EGFR* variants such as *EGFRvIII*, suggests that there may be a period between initial ecDNA formation and the development of more potent gain-of-function mutagenesis that could be amenable to therapeutic intervention and early cancer detection, for example, through liquid biopsy, which is under experimental investigation in brain tumors (49, 50). Such screenings are highly theoretical currently, however, as we neither know the duration of such a precancerous state, nor would we know whom to screen and when, highlighting the need for further investigation. Several of the oncogenes amplified on ecDNA have been the subject of targeted therapy trials, either alone or in combination, including *MET*, *CDK4*, *EGFR*, and *PDGFRA*, and other oncogenes are under experimental investigation, such as *CLOCK*, *SOX13*, and *AKT1* (51, 52). It will be important to determine whether tumors with and without precancerous ecDNA accumulation respond differently to targeted therapies in such trials, and ecDNA characterization may allow for such molecular stratification (Fig. 6J).

## Methods

### Patient Cohort

All 59 consecutive *IDH*wt adult GBM cases surgically treated at a single UK center (University Hospital Southampton, UK) between March 2017 and June 2020 were included in this cohort. Full ethical approval was granted through BRAIN UK for this study, with written informed consent from the patients as per BRAIN UK protocols; the study was conducted in accordance with the Declaration of Helsinki ethical guidelines; the study was approved by an Institutional Review Board at the Francis Crick Institute. We obtained surgical tissue specimens (FFPE) from the clinical archives of the Department of Cellular Pathology, University Hospital Southampton. Samples were obtained from routine surgical resection prior to starting chemotherapy or radiotherapy. *IDH* status was determined in routine clinical diagnostics, with IHC using the *IDH1* R132H mutation–specific antibody, supplemented with *IDH1*/*IDH2* sequencing as appropriate. All tumors retained *ATRX* expression.

The mean age of these patients was 61.6 years (range 38–80 years), with 36 males and 23 females; 32 tumors displayed *MGMT* methylation [known to increase sensitivity to chemotherapy (53)].

The FFPE specimens were used to generate tissue microarray blocks. For each tumor sample, three regions were selected for tissue microarray construction by a consultant neuropathologist, including (i) tumor core, representing a solid tumor and avoiding, as far as possible, tumor necrosis and microvascular proliferation; (ii) infiltrating zone, in which tumor cells are invading into brain parenchyma, which retains its essential structure; and (iii) leading edge, representing the most “normal” brain tissue in the specimen, mostly comprising cortical gray matter and lacking significant numbers of morphologically detectable tumor cells (*n* = 177 samples). Two independent neuropathologists confirmed the differentiation between the infiltrating zone and leading edge based on histology (17).

### WGS

Sequencing was performed using FFPE GBM samples in which tissue was available; a single 20 μm tissue section was used for DNA extraction using the QIAGEN QIAamp DNA FFPE Advanced Kit. Genomic DNA was randomly sheared into shorter DNA fragments, which were then end-repaired, A-tailed, and further ligated with Illumina adapters. These fragments with adapters were size-selected, PCR-amplified, and purified. Libraries were quantified through Qubit and qPCR; quantified libraries were pooled and sequenced by Illumina NovaSeq 6000 (150 bp paired-end reads) with 15-fold coverage per tumor.

### ecDNA Detection and Characterization

After aligning BAM files to GRCh38, CNVkit was used to detect DNA copy-number alterations. Candidate seed regions with a copy number greater than 3.5 and a size of more than 30 kbp were identified using the AmpliconSuite-pipeline (18), which were leveraged for ecDNA characterization with AmpliconArchitect and AmpliconClassifier (RRID:SCR_023150). CycleViz (https://github.com/AmpliconSuite/CycleViz) enabled the visualization of candidate circular ecDNA structures. Oncogenes were classified based on the ONGene database (54), as well as GBM driver genes previously reported [The Cancer Genome Atlas (55)].

### Nascent RNAscope and *EGFRvIII* IHC

We developed a method to detect nascent transcripts with RNAscope (which we term “nascent RNAscope”), based on probes for intronic sequences on *EGFR*/*PDGFRA*/*CDK4*. Although intronic probes have been used for ecDNA transcript localization with RNA FISH previously (56), the use of probes for RNAscope facilitates multiplexed imaging. Multiplex RNAscope was run on the BOND RX using reagents and the standardized protocol from ACD Bio-Techne; RNAscope probes for *EGFR*, *CDK4*, and *PDGFRA* were designed against intronic gene sequences. The slides were imaged on the Akoya Vectra Polaris multispectral slide scanner. This revealed high variability in the expression of these oncogenes from cell to cell, reflecting the variability in the copy number of these oncogenes (Supplementary Fig. S27A–S27D).

The FFPE slides were dewaxed and rehydrated to water through 100% and 70% ethanol. They were blocked with 0.3% hydrogen peroxide and then 5% BSA. The primary antibody [*EGFRvIII* Ab (D6T2Q) XP Rabbit mAb, Cell Signaling Technology, RRID:SCR_002071] was incubated at room temperature for 1 hour; then an anti-rabbit polymer was added for 1 hour. A DAB substrate was then added for 10 minutes. The slides were counterstained with hematoxylin and dehydrated through alcohols, and a coverslip was applied.

### DNA FISH and Microscopy

FISH was performed on 4 μmol/L FFPE tissue sections according to a combination of the Agilent Technologies Protocol (Histology FISH Accessory Kit, K5799; RRID:SCR_013575) and the Abbott Molecular Diagnostic FISH probe protocol (RRID:SCR_008392). Briefly, FFPE sections were dewaxed in xylene for 5 minutes, followed by rehydration in 100%, 80%, and 70% ethanol, and then washed twice with Agilent Technologies wash buffer. The FFPE tissue was then incubated at 98°C for 10 minutes in Agilent Technologies pretreatment solution. The Coplin jar was removed from the 98°C water bath and allowed to slowly cool for an additional 15 minutes. The FFPE slides were washed twice with Agilent wash buffer. Stock pepsin (Agilent stock pepsin) was applied to the slide for 10 minutes at 37°C. FFPE slides were washed twice with Agilent wash buffer and then dehydrated using 70%, 80%, and 100% ethanol prior to probe hybridization. Gene-specific probes containing chromosome-specific centromere enumeration probes against *PDGFRA* (Empire Genomics), *EGFR* (Vysis/Abbott), and *Myc* (Vysis/Abbott) were applied directly to the tissue sections with the coverslip being sealed with rubber solution glue.

Denaturation of the probes on the tissue was performed at 75°C for 7 minutes. The slide was then incubated overnight in a moist box at 37°C for 16 hours.

Slides were washed for 10 minutes at 73°C with 0.4× saline sodium citrate containing 0.3% IGEPAL (Sigma), followed by a 10-minute wash at room temperature with 2× saline sodium citrate containing 0.1% IGEPAL. Slides were allowed to air-dry and then counterstained with VECTASHIELD mounting medium containing DAPI (Thermo Fisher Scientific).

Images were captured using the VS200 Olympus Slide Scanner at 40× magnification. Quantification of FISH foci per nucleus was performed using QuPath software in a supervised fashion using the cell detection and subcellular cell detection features, with the “includeClusters” function to account for ecDNA clusters.

### Animals

All animal experiments were approved by the Institutional Animal Care and Use Committee of Memorial Sloan Kettering Cancer Center*. Myc*^*ec/+*^*; p53*^*fl/fl*^*; Nestin*-*Cre* and *Myc*^*+/+*^*; p53*^*fl/fl*^*; Nestin-Cre* were generated by crossing *Myc*^*ec*^ (JAX strain: 039221), *p53*^*fl*^ (B6.129P2-Trp53tm1Brn/J; strain: 008462), and *Nestin*-*Cre* [B6.Cg-Tg(Nes-cre)1Kln/J] mouse strains (RRID:SCR_016408). Brains from four-month-old animals were collected, sectioned, fixed in 10% formalin, and embedded in paraffin blocks. Induction of *Myc*-containing engineered ecDNA was verified by genomic PCRs to detect Cre-mediated circularization (forward: 5′-TGGAGTTGTCTCTGGTCTGTC-3′; reverse: 5′-AGCTTAGCTGAGAAATGAAGAGC-3′) and excision (forward: 5′-CATGTTGAACCAGAGTACAC-3′; reverse: 5′-GGATAACCGTGAGCTCCCAGC-3′) of the mouse *Myc*^*ec*^ allele.

### FISH

DNA FISH was performed on FFPE sections using a two-color probe. *Myc* (RP23-130M7, RP23-342F3, RP23-454G15) probes were labeled with red dUTP, and RP23-333G9 (15qA1) was labeled with green dUTP and served as the control. Probe labeling, hybridization, posthybridization washing, and fluorescence detection were performed according to procedures established at the Memorial Sloan Kettering Cancer Center Molecular Cytogenetics Core Facility. Slides were scanned using a Zeiss Axioplan 2i epifluorescence microscope (Zeiss Microscopy) equipped with Isis imaging software (MetaSystems Group Inc.).

### Computational Modeling of ecDNA-Driven Tumors

We developed the SPECIES framework to model expanding ecDNA-driven tumor populations in a two-dimensional space using an on-lattice cellular automaton model. Employing a kinetic Monte Carlo approach based on the method proposed by Bortz, Kalos, and Lebowitz (57) and similar previous spatial agent–based models of tumor growth (58, 59), we simulate the stochastic birth and death of tumor cells, as well as local cell displacement and random inheritance of ecDNA during cell division. Previous theoretical research into ecDNA dynamics in tumors supports the hypothesis of random segregation of ecDNA elements (16) described by a binomial partitioning process. We adopt the same approach to modeling ecDNA inheritance in our model. During cell replication, mother cells are able to displace neighboring cells in order to create the necessary space to divide.

See Supplementary Methods for full details on SPECIES. Additional model analysis can be found in Supplementary Figs. S28 to S34. Code for simulating and plotting spatial tumors can be found at https://github.com/MagnusHaughey/spatial_ecDNA_patterns and https://codeocean.com/capsule/9429295/tree/v1.



### Bayesian Inference of Patient Tumor Dynamics

We employ ABC (29, 30) with rejection sampling to fit our computational model to individual patient data. Specifically, we estimate the values of model parameters *k*, *s*, and *q*, which best recapitulate the fraction of ecDNA-free tumor cells and the single-cell ecDNA copy-number distribution from the tumor core and infiltrating margin of each patient’s tumor, derived using DNA FISH. We fixed the final simulated tumor size to $N_{\max}=10^{6}$ unless stated otherwise. To obtain spatial samples from each simulated tumor, we identify the core sample as the circular region of cells centered on the coordinates of the first tumor cell. The sampled ecDNA copy-number distributions can vary considerably from one location to another at the tumor-infiltrating margin. To capture this variability inherent to the system, we take 10 infiltrating margin samples from each simulated tumor, in which each margin sample is taken as a circular region centered around a cell at a radial distance of 75% of the tumor radius at a random angle. We pair the core sample with each of the 10 infiltrating margin samples and treat these as 10 independent core–margin sample pairs, comparing each with the patient data. To avoid contamination with nontumor cells, we filter out any single cells with an estimated ecDNA copy number of fewer than 3, both in the simulated and patient-derived samples. The size of the patient samples, after filtering, ranged between 583 and 6,742 cells (median = 1,396 cells), and we matched the size of each sampled simulation core and margin region to the corresponding regions in each tumor. We employed the Wasserstein metric (60,61) to quantifiably compare the ecDNA copy-number distributions in the simulated core and infiltrating margin samples with their patient-derived counterparts. To accept any given simulated core and infiltrating margin sample pair, we require that they are both, individually, sufficiently near to their patient-derived counterparts. For purposes of summarizing the resulting model fit, we then sum the Wasserstein distances for core and infiltrating margin samples to obtain the total distance between the simulation and patient data, denoted σ.

There are likely two main sources that contribute to the observed heterogeneity in inferred model parameters across patient tumors. First, there is some stochasticity and noise associated with the Bayesian inference process itself. The second, and more fundamental, source relates to differences in the underlying biology between tumors. For example, we see clear differences in the selection coefficients differentiating *EGFR-* and *PDGFRA*-ecDNAs. We also see some differences in selection coefficients within *EGFR-*ecDNAs across tumors. This observation is reasonable as some tumors carry *vIII*-mutated ecDNAs or other activating mutations or deletions, whereas other tumors present with only *EGFR* wild-type ecDNAs. We would expect that these differences tune the precise selective advantage of ecDNAs. The exact underlying mechanisms remain unknown, however, and merit further investigation in future studies. In some tumors, growth seems to start with ecDNA formation, suggesting a low *k*, whereas in others, ecDNA accumulates prior to expansion, suggesting a large *k*. As a result, we should expect to observe some variation in inferred *k* simply by chance as this depends on which cell acquired the additional activating event prior to clonal expansion.

See Supplementary Methods for further details on model inference.

### Data Availability

Sequence data have been deposited at the European Genome-phenome Archive, which is hosted by the EGI and the CRG, under accession number EGAS00001008126. ecDNA copy-number distributions can be accessed at https://github.com/MagnusHaughey/spatial_ecDNA_patterns.

## Supplementary Material

Supplementary Methods Supplementary Methods, providing details about methodology used throughout the study.

Supplementary Figures Supplementary Figures 1 to 34, detailing additional analyses.
