# Supplementary material for: Extrachromosomal DNA–Driven Oncogene Spatial Heterogeneity and Evolution in Glioblastoma
Source: Cancer Discov. 2025 Sep 8;15(10):2078–95. doi: 10.1158/2159-8290.CD-24-1555 (PMC12498097; doi:10.1158/2159-8290.CD-24-1555)
Supplement: Supplementary Methods — , providing details about methodology used throughout the study. [file cd-24-1555_supplementary_methods_suppsm.pdf]

Supplementary Methods for “Extrachromosomal DNA driven oncogene spatial heterogeneity and evolution in glioblastoma”

Imran Noorani*, Magnus Haughey*, Jens Luebeck, Andrew Rowan, Eva Grönroos, Francesco Terenzi, Ivy Tsz-Lo Wong, Davide Pradella, Marta Lisi, Jeanette Kittel, Natasha Sharma, Chris Bailey, Clare E Weeden, Donald M Bell, Eric Joo, Vittorio Barbè, Matthew G Jones, King L Hung, Emma L Nye, Mary Green, Lucy Meader, Emma J Norton, Mark Fabian, Nnennaya Kanu, Mariam Jamal-Hanjani, Thomas Santarius, Andrea Ventura, James AR Nicoll, Delphine Boche, Howard Y Chang, Vineet Bafna, Weini Huang, Paul S Mischel+, Charles Swanton+, Benjamin Werner+.

Contents

1 Spatial computational model of ecDNA-driven tumors **2**

1.1 Model initialization . . . 2

1.2 Random inheritance of ecDNA . . . 2

1.3 Cell displacement . . . 3

1.4 Differential selection . . . 3

1.5 Cell death . . . 4

1.6 Stochastic algorithm . . . 4

2 General model results **5**

3 Parameter inference in human glioblastoma **7**

4 Accuracy of ABC inference algorithm **9**

5 Varying spatial location of core samples **10**

6 Restricting parameter space of *k* **10**

7 Varying cell birth/death ratio **11**

8 Computational modeling of ecDNA+ neural stem cell *in vitro* clonal dynamics **11**

9 Modeling ecDNA variant dynamics **11**

10 Constant population size model of pre-expansion ecDNA dynamics **13**

11 Point mutation analysis of PCAWG *EGFR*-ecDNA tumors **14**

12 Modeling ecDNA co-amplification **14**

13 Estimation of DNA-RNA mapping on ecDNA **15**

1 Spatial computational model of ecDNA-driven tumors

1.1 Model initialization

Each individual agent (*i.e.* tumor cell) in our model is defined by its (x,y)-coordinate in the lattice, and the number of ecDNA copies it is carrying. Each lattice position may either be unoccupied or occupied by strictly one tumor cell. We initiate an individual simulation by seeding a cell in the center of a square lattice, carrying n copies of ecDNA. We specify the final size of the tumor in terms of number of cells, N_{max}, and estimate the approximate dimensions of the final system, assuming the final tumor to be perfectly circular, as $r = \sqrt{N_{max}/\pi}$ in units of lattice points. Independently of the value of ecDNA conferred replicative advantage, we assume the initial cell has already acquired the genetic alterations necessary for malignant growth and therefore has a birth/death ratio greater than 1.

After initialization, we begin simulating stochastic cell birth and death until the tumor has reached a total size of N_{max} cells. In its current form, the spatial model can only simulate tumors up to approximately $N_{max} = 10^6$ cells in a reasonable time-frame. Geometrically, if this were a cross-section of a 3D spherical tumor, lying on a plane containing the tumor center, this would correspond to a 3D tumor of approximately 10^9 cells (tumor volume of approximately 1 cm^3). If the patient tumors were significantly larger than 10^9 cells at the point of resection, we may under-estimate the selection strength of ecDNA-carrying cells, or the number of ecDNA copies in the tumor initiating cell, by comparing them to 2D simulated tumors of size approximately equal to 10^6 cells. For the current ABC inference, we simulated tumors of 10^6 cells, and acknowledge the limitations this might impose on the inferences made in our patient tumors.

1.2 Random inheritance of ecDNA

Inheritance patterns of ecDNAs were recently characterized in various tumor cell lines, and were well described by a binomial partitioning process [1]. In line with this evidence, we partition the ecDNA in a dividing mother cell into the two daughter cells in the following way: we assume each ecDNA element is replicated strictly once, and thus a mother cell containing X copies of ecDNA results in daughter cell copy numbers, x_1 and x_2, of

$$x_1 \sim Binomial(2X, \frac{1}{2}), \tag{1}$$

$$x_2 = 2X - x_1. \tag{2}$$

1.3 Cell displacement

Several approaches have been used in previous studies for implementing cell displacement in agent-based models, including non-linear cell pushing [2] mechanics and local cell motility [2–8]. Here, we model cell displacement using a linear cell pushing algorithm [8–12], which involves the creation of empty space in the neighborhood of dividing cells by pushing other cells in a straight line, filling a distal empty lattice point.

We implement cell pushing using the following method. After a cell has been selected for division, we identify its nearest empty point on the lattice. If there are multiple nearest lattice points, then one is chosen at random with uniform probability. Next, by drawing a straight line between the dividing cell and this empty lattice point, we construct a path of cells connecting these two locations. Last, each cell within this path is moved one position along the path, towards the empty lattice point (the dividing cell is not moved during this process). This process results in the filling of the empty lattice point, and the creation of a new empty space in the immediate neighborhood of the dividing cell, enabling it to divide into two daughter cells (one daughter cell occupies the old position of the parent cell, and the other is placed in the neighboring empty lattice point).

Spatial structure of the tumor influences the pattern of evolution [5, 8], and so we explore different tumor growth regimes in our simulations, achieved by varying a model parameter q which sets the range within which a dividing cell may search for a nearby empty lattice point. The value of q is always a global parameter i.e. all cells in the system are subject to the same q value. If there are no empty lattice points within a radius q of the dividing cell, the cell will not divide but will remain in its current position. Model parameter q can take any integer value greater than zero. Representing the strongest possible spatial constraints: when $q = 1$, dividing cells may only search for empty lattice points within a radius of one lattice unit (von Neumann neighborhood). By setting q to be very large, we are able to remove all spatial constraints (in this study, we typically set $q = 1000$ to represent this regime).

1.4 Differential selection

We assume that presence of ecDNA can confer a positive selective advantage to a tumor cell, and implement this by means of an increase in replication rate. In general, it is possible to implement any mathematical function to describe ecDNA-conferred cell fitness, however we adopt a simple ecDNA copy

number independent model, leading to a cell replication rate, r_b, of

$$r_b(x,s) = \begin{cases} 1 & x = 0 \\ 1+s & x > 0 \end{cases} \tag{3}$$

where x denotes the ecDNA copy number in the cell and s is a model parameter which specifies the strength of positive selection. Neutral selection, where ecDNA presence confers no replicative advantage to the cell, is achieved by setting $s = 0$, whereas $s > 0$ describes positive selection. Whilst it may be reasonable to expect the replication rate of a cell to depend more strongly on the number of ecDNA-amplified oncogenes, copy number independent selection models have been employed in other mathematical models [1, 13], and have been shown to adequately describe the dynamics of ecDNA driven tumors.

1.5 Cell death

We model cell death as an independent event in the simulations, uncoupled from cell division. The rate of cell death, r_d, is fixed at some fraction of the neutral cell division rate, $r_b(x, 0)$,

$$r_d = r_b(x,0) \cdot \alpha, \tag{4}$$

where s denotes the strength of ecDNA conferred replicative advantage, x represents the ecDNA copy number in the cell and $\alpha \in [0, 1]$, which was set at $\alpha = 0.5$ throughout this study unless stated otherwise. Following a cell death event, the dead cell is immediately removed from the lattice, leaving behind an empty lattice point.

1.6 Stochastic algorithm

We adopt a kinetic Monte Carlo approach based on the method proposed by Bortz, Kalos and Lebowitz [14]. For each iteration of the algorithm, we first select the event to occur by considering the total sum of all event rates in the system, before choosing the cell in which the event will take place.

In our model we consider stochastic cell birth and death which, in a cell with x copies of ecDNA, occur with rates $r_b(x, s)$ and r_d respectively. As the rate of cell death does not depend on the ecDNA copy number, the total rate of cell death in the system, R_d , is

$$R_d = N \cdot r_d, \tag{5}$$

where N denotes the total number of cells present in the system. In its most general form, we partition cell birth into separate events for each value of ecDNA copy number, thus allowing for ecDNA copy number dependent selection functions. Denoting N_x as the number of cells with x copies of ecDNA, the

sum of birth rates across all cells with copy number x, R_b^x, is given as

$$R_b^x = N_x \cdot r_b(x, s). \tag{6}$$

For each iteration of the algorithm, we therefore compute the sum of birth and death rates across all cells in the system, R_{tot}, as

$$R_{tot} = R_d + \sum_{x=0}^{x_{max}} R_b^x, \tag{7}$$

where x_{max} denotes the largest single-cell ecDNA copy number in the system. To determine which event type will occur, we generate a uniform random variable $\alpha \in \text{Uniform}(0, 1)$ and select cell death if

$$\alpha \cdot R_{tot} < R_d. \tag{8}$$

Otherwise, division occurs for a single cell with x ecDNA copies, for x satisfying

$$\sum_{i=0}^{x-1} R_b^i < (\alpha \cdot R_{tot} - R_d) \leq \sum_{i=0}^{x} R_b^i. \tag{9}$$

Once the type of event has been determined, a cell to undergo the event must be chosen. We thus choose, with uniform probability, a single cell from the pool of all eligible cells (*e.g.* if the event is division of a cell with x copies of ecDNA, then all cells in the system containing x ecDNA are eligible to be chosen).

For the majority of the modeling performed in this study, we employed a simple ecDNA copy number independent model of selection. This greatly simplifies the process of selecting events since all ecDNA copy number states of $x > 0$ can be combined into one single group with combined rate, denoted $R_b^{x>0}$, given as

$$R_b^{x>0} = N_{x>0} \cdot r_b(x > 0, s), \tag{10}$$

where $N_{x>0}$ denotes the number of cells containing one or more copies of ecDNA.

2 General model results

We implemented the spatial computational model set out above to study the effects of initial ecDNA copy number, k, ecDNA conferred selection strength, s, and the strength of spatial constraints, q, on the resulting spatial patterns of ecDNA copy number in the expanded tumor (Supplementary Figure 9). When model parameter q is sufficiently large ($q = 1000$ in our implementation) all spatial constraints are removed and our model recapitulates previously published results, derived using non-spatial models, describing the time evolution of the single-cell ecDNA copy number distribution and the fraction of tumor

cells carrying zero copies of ecDNA [1] (Supplementary Figure 8).

Our model builds upon previously studied models not only by explicitly including spatial constraints, but also by allowing for different numbers of ecDNA copies in the initial tumor cell. For tumors with no spatial constraints, both small and large values of initial cell ecDNA copy number, k, lead to a mean single-cell ecDNA copy number which is spatially homogeneous across the entire tumor (Supplementary Figure 28). When spatial constraints are included, mean copy number is either constant with increasing radial distance from the tumor center when ecDNA confers no selective advantage ($s = 0$) or increasing approximately linearly when ecDNA-positive cells are under positive selection ($s > 0$). The mode of growth of the tumor and the initial ecDNA copy number also affect the variation in ecDNA copy number at the single cell level. For non-spatially constrained tumors, the standard deviation of single cell ecDNA copy number is roughly constant, or weakly increasing, with radial distance from the tumor center (Supplementary Figure 29). Conversely, for tumors with strong spatial constraints, variation in single-cell copy number increases more markedly as cells are sampled further away from the tumor center. For $k = 1$, the standard deviation is lower for neutral dynamics, compared to cases of positive selection, both for weak and strong spatial constraints. Interestingly, this is reversed for larger initial ecDNA copy numbers, with neutral dynamics leading to larger standard deviation. This is likely a result of the ecDNA-negative state being an absorbing state: once a cell has lost all its copies of ecDNA, it may never regain them (since we do not model *de novo* ecDNA production). For tumors which begin a cell with a single copy of ecDNA, many resulting descendant cell lineages will be stuck in the ecDNA-negative state, driving down the overall variation in ecDNA copy number at the single-cell level. When tumors begin with many copies of ecDNA, neutral dynamics lead to larger variation as fewer cells are ecDNA-negative. When $s > 0$, however, those ecDNA-negative cells which arise in the tumor are selected against, and thus their relative fraction within the tumor population is reduced, driving down the variation at the single-cell level.

The initial number of ecDNA copies has a large impact on the probability of the tumor retaining or losing ecDNA during expansion. Simulations of tumor growth from a single cell revealed that a starting ecDNA copy number of 1 led to complete loss of ecDNA in more than 70% of fully formed tumors in the absence of ecDNA conferred fitness advantages, with many tumors also failing to maintain ecDNA populations even when ecDNA give rise to a positive selective advantage. In contrast, presence of 20 ecDNA copies in the initiating cell resulted in near ubiquitous maintenance of ecDNA during tumor formation (Supplementary Figure 18).

The impact of including spatial constraints are most pertinent when one compares the overall single-cell ecDNA copy number distribution for tumors with and without spatial constraints (Supplementary Figure 30). Our model shows that including spatial constraints leads to a wider overall distribution, with more cells at both low and high extremes of ecDNA copy number, with the exception of when $s = 0$ and $k = 1$. This increased variation, under-represented by previous non-spatial models, could increase the

ability of the tumor to adapt quickly to changing selective pressures, such as introduction of chemotherapy, targeted therapies or radiotherapy.

The underlying source of the increased variation for tumors with and without spatial constraints can be understood by comparing the distribution of cell lineage lengths in each case (Supplementary Figures 30c, d). When there are no spatial constraints, all cells are equally capable of dividing (under neutral dynamics) and thus the distribution of cell lineages in the final tumor is tightly peaked around a mean value (theoretical mean for a tumor of size $N_{max} = 10^5$ cells is $\log_2(10^5) \approx 16.6$, however for our simulations this mean was marginally greater as a result of our assumption that cell cycle times are exponentially distributed). Spatial constraints during tumor growth lead to surface driven dynamics, whereby only cells situated at the expanding edge of the tumor are capable of division. This leads to a spatial bottleneck, resulting in some cell lineages (those surfing at the leading edge) to be significantly greater than others (*e.g.* those for cells trapped within the interior of the tumor) and a larger overall mean cell lineage length. Since ecDNA is binomially distributed at each cell division, increasing the variation in single-cell ecDNA copy number in each instance, tumors with stronger spatial constraints, and therefore a higher mean cell lineage length, will have a wider and more varied distribution of ecDNA copy numbers.

3 Parameter inference in human glioblastoma

We employ approximate Bayesian computation (ABC) [15,16] with rejection sampling to fit our computational model to individual patient data. Our method of parameter inference can be summarized in the following steps:

1. Define prior distributions for model parameters k, s and q.
2. Sample candidate parameter values from prior distributions, k^*, s^* and q^*, and execute a spatial simulation with these model values as input parameters.
3. Sample regions of simulated tumor from the tumor core and infiltrating margin, and derive single-cell copy number distributions for both sampled regions.
4. Quantitatively compare both patient single-cell copy number distributions and fraction of ecDNA-free tumor cells from tumor core and infiltrating margin to their simulated counterparts.
5. If simulated data is sufficiently similar to patient data, add parameter values k^*, s^* and q^* to posterior sample set.
6. Return to step 2 and repeat until the posterior sample set is of size N.

For the parameter estimation performed in this study, we specify prior distributions $P(k)$, $P(s)$ and $P(q)$ of

$$P(k) = \text{Uniform}(1, 150), \tag{11}$$

$$P(s) = \text{Uniform}(-0.5, 5), \tag{12}$$

$$P(q) = 1, 2, 5, 10, 50, 1000. \tag{13}$$

To obtain spatial samples from simulated tumors, we identify the core region as the circular region of cells centered on the coordinates of the first tumor cell. We sample 10 infiltrating margin samples from each simulated tumor, each taken as circular regions centered around a cell a distance of 75% of the tumor radius, each at a random angle. We pair the core sample with each of the 10 infiltrating margin samples and treat these as independent samples when comparing simulated to patient data. Additionally, we match the size of each sampled core and infiltrating margin region to the corresponding regions in each patient.

Prior to comparing simulated and patient data, we filter all patient core and infiltrating margin samples to remove any cells with an estimated ecDNA copy number fewer than three (referred to as low-ecDNA cell fraction herein). This is because our estimates of ecDNA copy number in this range, derived using image analysis software applied to DNA FISH images (cross reference to methods section about DNA FISH), are particularly noisy. Furthermore, from DNA FISH images alone, we are unable to distinguish between non-tumor cells and tumor cells which are carrying zero copies of ecDNA. Knowledge of the low-ecDNA cell fraction is, however, an important indicator of the underlying tumor dynamics. To exploit this information, we record the fraction of tumor cells removed in the patient data during filtering, and use these measurements as additional data with which to fit patient to simulated data.

To quantify the similarity of simulated and patient derived ecDNA copy number distributions, we thus combine the use of two metrics. First, we employ the Wasserstein distance [17, 18], a commonly used metric in statistical inference and machine learning. During the data comparison stage of our ABC algorithm (step 5 above), we compute the Wasserstein distance between simulated and patient ecDNA copy number distributions, both for the tumor core and infiltrating margin, and denote these distances as δ_1^c and δ_1^m respectively. Second, we compare the low-ecDNA cell fraction for the patient and simulated data, both for the core and infiltrating margin regions, using the expression

$$\delta_2 = \frac{|n_{sim} - n_{pat}|}{N_{pat}}, \tag{14}$$

where n_{sim} and n_{pat} denote, respectively, the sample size in the simulated and patient data after filtering low-ecDNA cells, and N_{pat} denotes the sample size in the patient prior to filtering. We compute this comparison metric both for the core and margin regions, and denote these as δ_2^c and δ_2^m respectively. In order to accept a simulation into the posterior sample set, we require that each simulation/patient comparison metric must be smaller than some threshold value, *i.e.*

$$\delta_1^c < \epsilon_1^c, \tag{15}$$

$$\delta_1^m < \epsilon_1^m, \tag{16}$$

$$\delta_2^c < \epsilon_2^c, \tag{17}$$

$$\delta_2^m < \epsilon_2^m, \tag{18}$$

where threshold values on low-ecDNA cell fraction similarity, ϵ_2^c and ϵ_2^m, were chosen so as to optimize the accuracy of the inference algorithm. Threshold values on the Wasserstein distances, ϵ_1^c and ϵ_1^m, were determined on a patient-specific basis such that the ABC inference algorithm achieved an acceptance rate of $\leq 5\%$.

After a sufficiently large number of repetitions, we obtain a good approximation for the posterior distribution of $(k,\ s,\ q)$ which represents the regions of the parameter space for which there is most model support. From these distributions, we estimate the optimal parameter values by measuring the median of the 1-dimensional marginal distributions for each of k, s and q. We estimate errors as the variance of these marginal distributions. For a small number of patients, one of the parameter posterior distributions is bimodal, which may be a result of projecting the 3-dimensional posterior distribution onto 1-dimensional axes.

4 Accuracy of ABC inference algorithm

We explored the sensitivity and specificity of our model inference approach by applying it to a large set of simulated tumor core and infiltrating margin pairs, generated using the computational model itself. We generated an artificial patient dataset consisting of 100 simulated tumors for each combination of $k \in \{1, 25, 50, 100\}$, $s \in \{0, 0.5, 1, 2\}$ and $q \in \{1, 5, 50, 1000\}$ (Supplementary Figure 31). From each simulated tumor, we extracted a sample of tissue from the tumor core and from the outer edge at a randomly chosen angle. All tumors were simulated up to a final size of 10^5 cells and sampled regions were fixed at a size of 5000 cells.

To determine the optimal values for thresholds $(\epsilon_1^c,\ \epsilon_1^m,\ \epsilon_2^c,\ \epsilon_2^m)$, we computed the sensitivity and specificity (true positive and true negative rate respectively) across the range of parameter values represented in our artificial patient dataset, repeating this for a range of threshold values. We selected the combination of threshold values which maximized the sensitivity and specificity of the algorithm (Supplementary Figure 32a).

Results from this test demonstrate that the inferences made by the algorithm are reasonably accurate

across all parameter values represented in the artificial patient dataset (Supplementary Figure 32b). We chose to present the model inference accuracy in terms of sensitivity and specificity for two main reasons. First, we decided to parse the values of the selection coefficient, s, into two categories: "neutral" or "positive" selection (corresponding to $s = 0$ and $s > 0$ respectively) since this general classification is biologically the most important. Second, the effects of the model parameters, particularly q, do not scale linearly with the parameter value (*e.g.* the difference between $q = 50$ and $q = 1000$ on the resulting ecDNA patterns and statistics is very small, the difference between $q = 1$ and $q = 10$ is much larger).

Sensitivity and specificity of the algorithm is particularly high across the full range of k and q values we tested, with the algorithm particularly able to recover the true underlying k value in the patient data. Due to our use of a constant selection model in our simulations, we were less successful at determining the true underlying selection coefficient, s. This loss of sensitivity is likely due to the fact that in tumors with a high fraction of ecDNA-positive cells, neutral competition will mostly ensue regardless of the specific value of s. Despite this, the inference algorithm still displayed moderate sensitivity when we pool all positive selection coefficients and simply predict neutral ($s = 0$) or positive ($s > 0$) selection. A prediction of neutral selection is made if the error on the point estimator for the selection coefficient, s, encompasses $s = 0$, otherwise the sample is classed as being consistent with positive selection.

5 Varying spatial location of core samples

When sub-sampling tissue from our simulated tumors, the core sample is always centered around lattice coordinates of the tumor initiating cell, however the same regularity cannot be obtained when sampling tissue from real tumors. To understand how this aspect of the model affects our measured ecDNA copy number distributions, and thus our inferred model parameters, we employed an alternative tissue sampling scheme, in which we sampled tissue from a region a short radial distance from the coordinates of the tumor initiating cell, at a random angle. We repeated the parameter inference using this alternative approach, but found the same oncogene-level patterns of inferred parameters, suggesting that variations in core sample location is not responsible for this observed pattern.

6 Restricting parameter space of k

To further investigate the model prediction in some patients of high initial ecDNA copy number, k, we repeated the parameter inference whilst constricting the value of k to $k = 1$ only (Supplementary Figure 21). For each patient, we determined the optimal model fit to the ecDNA copy number distribution and computed the difference in affinity between model and data, denoted $\Delta\sigma$, for the normal parameter inference and the n-restricted case. We found that the model best-fit in the k-restricted inference was

notably worse (indicated by a large $\Delta\sigma$) in patients predicted to have a high k in our initial parameter inference. Whilst this does not rule out alternative explanations such as copy number dependent selection or co-selection/co-segregation dynamics, this supports the notion that, within the context of our model, a high value of k is the only sufficient explanation for the observed ecDNA copy number distributions in some of our patients.

7 Varying cell birth/death ratio

Throughout all model simulations in this study, we maintained a constant cell death/birth ratio of $\psi = 0.5$. To test the sensitivity of our patient inferences on this parameter, we repeated the patient model fitting with death/birth ratios of $\psi = 0.1$, leading to few cell deaths during expansion, and $\psi = 0.9$, giving rise to near-balanced cell birth and death (Supplementary Figure 20). Inferred model parameters for each patient were very similar under these two alternative regimes to the original inferred values using $\psi = 0.5$, suggesting weak sensitivity to this model parameter. Inferences for model parameter k were particularly robust to these changes. Reducing the death/birth ratio to $\psi = 0.1$ slightly reduced the inferred ecDNA selection strength, s, and cell pushing strength, q, whilst an increased value of ψ led to slightly greater inferred values of s and q.

8 Computational modeling of ecDNA+ neural stem cell *in vitro* clonal dynamics

We adapted the SPECIES computational framework to model the *in vitro* clonal dynamics of primary adult neural stem cells from *Myc*$^{\mathrm{ec}/+}$ *Trp53*$^{\mathrm{fl/fl}}$ mice (infected with Ad-cre) [19]. To model these data, we ran the SPECIES model in the limit of no spatial constraints ($q = 1000$), and included a carrying capacity (to model the *in vitro* population approaching confluency) and re-sampling at a prescribed rate (to model re-plating of the *in vitro* population). At the beginning of each simulation, we seeded an initial population of cells, a fraction of which carrying a single ecDNA copy. Cells were assumed to have a basal doubling time of 24 hours, with an elevated death/birth ratio for the first 10 days of the experiment to reflect stresses experienced by cells following the initial plating. In line with the experimental data of Pradella *et al.* we simulated the system for 5 weeks, measuring the ecDNA copy number distribution every 1 week. All model parameters are summarized in Table 1.

9 Modeling ecDNA variant dynamics

Both structural variant and immunohistochemistry analysis confirmed the presence of *EGFR* mutations amplified on ecDNA *in vivo*. We modeled the initial dynamics of wild-type ecDNA, followed by the emer-

Parameter	Value
Cell doubling time	24h
Death/birth ratio (≤ 10 days)	0.9
Death/birth ratio (> 10 days)	0.5
Initial population size	10,000
Carrying capacity	300,000
Initial ecDNA+ cell fraction	0.01
Initial ecDNA copy number	1
Re-plate rate	2/week
Re-plate population size	10,000

Table 1: Model parameters used to simulate *in vitro* ecDNA+ clonal dynamics.

gence of an advantageous variant of the gene on ecDNA within the framework of SPECIES. To explore the dynamics of the wild-type and variant ecDNA, we varied the following model parameters: $k_{wt} > 0$ and $k_{var} \geq 0$, which respectively set the number of wild-type and variant ecDNA copies in the clone-initiating cell; and $V_{var} \geq 0$, which specifies the size to which the tumor must expand (in terms of total number cells) before the mutant ecDNA emerges from within the existing wild-type ecDNA population. Setting $V_{var} = 0$ gives rise to a tumor with "pre-expansion" mutant ecDNA, the number of which in the clone-initiating cell is specified by k_{var}, whereas $V_{var} > 0$ leads to a tumor with "post-expansion" mutant ecDNA.

We model evolutionary dynamics by assuming that wild-type and variant ecDNA confer a replicative advantage to tumor cells, with associated selection coefficients denoted s_{wt} and s_{var} respectively, with $s_{wt} \leq s_{var}$. Specifically, a tumor cell with x_{wt} copies of the wild-type ecDNA and x_{var} copies of variant ecDNA will have a replicative rate, r_b, given by

$$r_b(x_{wt}, x_{var}, s_{wt}, s_{var}) = \begin{cases} 1 & (x_{wt} = 0 \quad \text{and} \quad x_{var} = 0), \\ 1 + s_{wt} & (x_{wt} > 0 \quad \text{and} \quad x_{var} = 0), \\ 1 + s_{var} & (x_{var} > 0), \end{cases} \tag{19}$$

where we assumed that the wild-type ecDNA conferred a weakly positive advantage, $s_{wt} = 0.2$, and the mutated ecDNA conferred a stronger advantage, $s_{var} = 2$. We further assume that each ecDNA is replicated once during cell division, and that all ecDNAs are partitioned binomially across the two resulting daughter cells. We set the final simulated tumor size to be $N_{max} = 10^5$, and fixed $q = 2$, since this was the modal value of inferred q for *EGFR*-ecDNA tumors in the GB-UK cohort.

Using this model, we computed the mean measured ecDNA heteroplasmy (percentage of ecDNA copies in sample carrying wild-type version of the gene) in the tumor core for each combination of $k_{wt} \in \{1, 10, 50\}$ and $k_{var} \in \{1, 10, 50\}$ for pre-expansion ecDNA mutation ($V_{var} = 0$), and $k_{wt} \in \{1, 10, 50\}$ and $V_{var} \in \{10^2, 10^3, 10^4\}$ for post-expansion ecDNA mutation ($k_{var} = 0$) (Supplementary Figures 33 & 34). We simulated 1,000 tumors for each parameter combination, and computed the mean core hetero-

plasmy for each combination of parameters. To measure core heteroplasmy, we extracted 2,000 cells from the core of each simulated tumor and computed the ecDNA heteroplasmy as $X_{wt}/(X_{wt} + X_{var})$, where X_{wt} and X_{var} denote the total number of wild-type and variant ecDNA copies, pooled across the entire sampled set of cells. These circular core samples were taken a radial short distance from the coordinates of the tumor initiating cell, at a random angle, in order to emulate the variation in sample location in the patients' samples.

Alongside measuring ecDNA heteroplasmy in the tumor core, we computed the probability of observing any variant ecDNA in randomly sampled regions of the tumor core and infiltrating margin (Main Figure 3g & Supplementary Figure 33). For the same combinations of parameters set out above, we simulated 1,000 tumors, sampling 5 core & margin pairs from each tumor, with each regional sample consisting of 2,000 cells.

In addition to our chosen selection coefficients of $(s_{wt}, s_{var}) = (0.2, 2.0)$, we explored ecDNA variant heteroplasmy dynamics for two additional parameter regimes, which were $(s_{wt}, s_{var}) = (0.2, 0.2)$ and (2.0, 2.5) (Supplementary Figure 33). When *EGFRvIII* does not confer any additional fitness advantage, *i.e.* $(s_{wt}, s_{var}) = (0.2, 0.2)$, there is less model support in particular for maintaining *EGFRvIII*-ecDNA across the tumor core and margin (Supplementary Figure 33(i)). The *EGFR* heteroplasmy dynamics are similar under $(s_{wt}, s_{var}) = (2.0, 2.5)$ as they were under our original choice of $(s_{wt}, s_{var}) = (0.2, 2.0)$ however, notably, parameter combinations $(k_{wt}, k_{var}, V_{var}) = (10, 1, 0)$ and $(k_{wt}, k_{var}, V_{var}) = (50, 1, 0)$ again have less model support for maintaining vIII across the tumor core and margin (Supplementary Figure 33(ii)) than they did for $(s_{wt}, s_{var}) = (0.2, 2.0)$. These additional simulations highlight that the absolute selection coefficients are perhaps not as important as their relative differences in determining the ecDNA heteroplasmy dynamics, and that factors such as the fitness plateau (a feature of models which employ constant ecDNA fitness effects) can make it difficult to know the exact fitness differences between *EGFRwt* and *EGFRvIII.* Inferring these parameters together with other parameters would vastly expand the free parameter space and, with the available patient data, estimating these with precision is not possible.

10 Constant population size model of pre-expansion ecDNA dynamics

To further understand the dynamics of wild-type and variant ecDNA in the pre-malignant tissue, we exploited the simulation framework of SPECIES to model a non-spatial, constant population size cell population. These simulations follow the dynamics between the generation of the first wild-type ecDNA up to the first mutation of an ecDNA. After the system of cells is initialized (fixed at 1,000 cells), one cell in the system is selected randomly to acquire a single copy of the wild-type ecDNA. The system

is then evolved following a Moran process [20], assuming the same dynamics of ecDNA as the original SPECIES simulations (ecDNA replication and random inheritance). The model of ecDNA selection is also the same as the original SPECIES model, with the wild-type ecDNA conferring a weakly positive advantage to the host cell ($s = 0.2$). During cell division, each copy of wild-type ecDNA in the mother cell is replicated once, and may stochastically mutate to the variant ecDNA with rate μ per ecDNA replication. We simulate the system up until the first ecDNA mutation, at which point we measure the ecDNA heteroplasmy of the cell in which the mutation occurred.

11 Point mutation analysis of PCAWG *EGFR*-ecDNA tumors

We analyzed point mutations on *EGFR* within *EGFR*-ecDNA amplified tumors in the PCAWG cohort using the available point mutation VAF (variant allele frequency) data generated by the TCGA Research Network: (https://www.cancer.gov/tcga). We corrected the variant allele frequencies for tumor purity by considering the following equivalence:

(#supporting variant reads in tumor & normal tissue) = (#supporting variant reads in tumor tissue)

which is equivalent to

(measured VAF) × (#loci from tumor & normal DNA) = (true VAF) × (#loci from tumor DNA).

This can be written mathematically as

$$f \times (pn_t + (1-p)n_n) = \hat{f} \times pn_t \tag{20}$$

where $\hat{f}$ = corrected VAF; f = measured VAF; p = tumor purity; n_t = copy number of loci in tumor (approximated as the median ecDNA copy number in sample); and n_n = copy number of loci in normal tissue (assumed to be = 2). This gives the following expression for the tumor purity corrected VAF, $\hat{f}$, as

$$\hat{f} = \frac{f}{p} \times \frac{p(n_t - n_n) + n_n}{n_t}. \tag{21}$$

Computational analysis of point mutation expansion was performed using the same methodology as for *EGFR* structural variants, outlined in Supplementary Methods section 9.

12 Modeling ecDNA co-amplification

Whole-genome sequencing of the core tumor samples shed further light on the extent of ecDNA co-amplification in *in vivo* GBM. Of the 57 tumor cores we sequenced, we found 8 which contained multiple oncogene-amplifying ecDNA amplicons. Recently co-segregation (correlated inheritance) and co-selection

(selective advantage for maintaining a mixture of both ecDNA species within a cell) has been shown to play a role in maintaining ecDNA populations in tumor cell lines [13]. Our computational model only accounts for a single ecDNA type, however, and thus does not capture these important dynamics in our patient-derived samples. In particular, it may be that co-segregation and co-selection dynamics could provide an alternative explanation for a subset of patient samples which were previously consistent with a large number of pre-existing ecDNA copies (*i.e.* high inferred k).

To explore this, we adapted SPECIES to include two ecDNA types. We implemented co-segregation dynamics using the same approach as Hung *et al.* [13] and extended our constant selection model to account for both types of ecDNA. In this adapted model, a tumor cell with x_1 copies of ecDNA type 1 and x_2 copies of ecDNA type 2 will have a replicative rate, r_b, given by

$$r_b(x_1, x_2, s_p, s_m) = \begin{cases} 1 & (x_1 = 0 \quad \text{and} \quad x_2 = 0), \\ 1 + s_p & (x_1 > 0 \quad \text{and} \quad x_2 = 0), \\ 1 + s_p & (x_1 = 0 \quad \text{and} \quad x_2 > 0), \\ 1 + s_p + s_m & (x_1 > 0 \quad \text{and} \quad x_2 > 0), \end{cases} \tag{22}$$

where $s_p \geq 0$ denotes the strength of positive selection for the "pure" state *i.e.* when only 1 type of ecDNA is present, and $s_m \geq 0$ for the "mixed" state *i.e.* containing both types of ecDNA. We fixed the value of the co-segregation parameter to 0.6, consistent with the measured value in GBM39 cells by Hung *et al.*

In principle one could obtain ecDNA copy number distributions for both ecDNA types using this alternative model, however we had only single-cell resolution copy number data for one of the two ecDNA amplicons in each patient (with the exception of patient A5, for whom we had data both for the EGFR- and PDGFRA-amplifying ecDNA amplicons). We thus sampled data for one ecDNA type when fitting the co-amplified ecDNA model to the patient data, however this meant we were unable to reliably infer specific values co-segregation and co-selection.

13 Estimation of DNA-RNA mapping on ecDNA

To understand the relationship between ecDNA copies and transcription, we developed a computational tool that finds an optimized scaling parameter to infer the correlation between the ecDNA copies and the expression levels. We implemented a Monte Carlo inference which adds a decreasing exponential noise, parameterized by λ, such that the distance between the nascent RNAscope and the DNA-FISH count distributions for a given patient sample is minimized. The framework starts by removing cells with 0, 1 and 2 ecDNA copies, to take into account the uncertainty of quantifying ecDNAs from FISH in cells with few copies. We then sample a random value λ_i from a uniform distribution $\mathrm{Uniform}(0.1, 4)$

and reconstruct a scaled DNA-FISH counts k_i' by sampling from an exponential decreasing probability function for every ecDNA copy k found in the original DNA-FISH distribution, *i.e.*

$$k_i' \sim \text{Exp}(-\lambda i k). \tag{23}$$

We compute the distance between the scaled DNA distribution, x_i, and the RNAscope distribution, y, using three distances, $E(x_i, y)$, $M(x_i, y)$ and $K(x_i, y)$, where E and M are relative Euclidean distance for the entropy and the mean of the distributions respectively, and K is the Kolmogorov-Smirnov statistic. These distances are grouped into a vector of summary statistics $S(x_i, y) = [E(x_i, y), M(x_i, y), K(x_i, y)]$. We tested a total of $100,000$ random λ_i values for each patient, and constructed the posterior distribution of λ using the 500 values which give rise to the smallest $S(x_i, y)$. We then measure the modal value of the posterior distribution to find the optimal scale parameter λ^*. In Supplementary Figure 27, we plotted for all patients the Wasserstein distance between the matched DNA-FISH counts scaled by λ^* and the RNAscope distributions.

References

[1] Lange, J. T. *et al.* The evolutionary dynamics of extrachromosomal dna in human cancers. *Nature Genetics* **54**, 1527–1533 (2022). URL https://doi.org/10.1038/s41588-022-01177-x.

[2] Waclaw, B. *et al.* A spatial model predicts that dispersal and cell turnover limit intratumour heterogeneity. *Nature* **525**, 261 EP – (2015). URL https://doi.org/10.1038/nature14971.

[3] Thalhauser, C. J., Lowengrub, J. S., Stupack, D. & Komarova, N. L. Selection in spatial stochastic models of cancer: Migration as a key modulator of fitness. *Biology Direct* **5**, 21 (2010). URL https://doi.org/10.1186/1745-6150-5-21.

[4] M A, M., Kim, J.-Y., Pan, C.-H. & Kim, E. The impact of the spatial heterogeneity of resistant cells and fibroblasts on treatment response. *PLOS Computational Biology* **18**, e1009919 (2022). URL https://doi.org/10.1371/journal.pcbi.1009919.

[5] West, J., Schenck, R. O., Gatenbee, C., Robertson-Tessi, M. & Anderson, A. R. A. Normal tissue architecture determines the evolutionary course of cancer. *Nature Communications* **12**, 2060 (2021). URL https://doi.org/10.1038/s41467-021-22123-1.

[6] Anderson, A. R. A., Weaver, A. M., Cummings, P. T. & Quaranta, V. Tumor Morphology and Phenotypic Evolution Driven by Selective Pressure from the Microenvironment. *Cell* **127**, 905–915 (2006). URL https://doi.org/10.1016/j.cell.2006.09.042.

[7] Ghaffarizadeh, A., Heiland, R., Friedman, S. H., Mumenthaler, S. M. & Macklin, P. PhysiCell: An open source physics-based cell simulator for 3-D multicellular systems. *PLOS Computational Biology* **14**, e1005991 (2018). URL https://doi.org/10.1371/journal.pcbi.1005991.

[8] Noble, R. *et al.* Spatial structure governs the mode of tumour evolution. *Nature Ecology & Evolution* **6**, 207–217 (2022). URL https://doi.org/10.1038/s41559-021-01615-9.

[9] Chkhaidze, K. *et al.* Spatially constrained tumour growth affects the patterns of clonal selection and neutral drift in cancer genomic data. *PLoS computational biology* **15**, e1007243–e1007243 (2019). URL https://pubmed.ncbi.nlm.nih.gov/31356595https://www.ncbi.nlm.nih.gov/pmc/articles/PMC6687187/.

[10] Ryser, M. D., Min, B.-H., Siegmund, K. D. & Shibata, D. Spatial mutation patterns as markers of early colorectal tumor cell mobility. *Proceedings of the National Academy of Sciences* **115**, 5774–5779 (2018). URL https://www.pnas.org/content/115/22/5774. https://www.pnas.org/content/115/22/5774.full.pdf.

[11] Fu, X. *et al.* Spatial patterns of tumour growth impact clonal diversification in a computational model and the TRACERx Renal study. *Nature Ecology & Evolution* **6**, 88–102 (2022). URL https://doi.org/10.1038/s41559-021-01586-x.

[12] Househam, J. *et al.* Phenotypic plasticity and genetic control in colorectal cancer evolution. *Nature* **611**, 744–753 (2022). URL https://doi.org/10.1038/s41586-022-05311-x.

[13] Hung, K. L. *et al.* Coordinated inheritance of extrachromosomal dna species in human cancer cells. *bioRxiv* (2023). URL https://www.biorxiv.org/content/early/2023/07/19/2023.07.18.549597.

[14] Bortz, A., Kalos, M. & Lebowitz, J. A new algorithm for monte carlo simulation of ising spin systems. *Journal of Computational Physics* **17**, 10–18 (1975). URL https://www.sciencedirect.com/science/article/pii/0021999175900601.

[15] Beaumont, M. A., Zhang, W. & Balding, D. J. Approximate Bayesian Computation in Population Genetics. *Genetics* **162**, 2025–2035 (2002). URL https://doi.org/10.1093/genetics/162.4.2025.

[16] Csilléry, K., Blum, M. G., Gaggiotti, O. E. & François, O. Approximate bayesian computation (abc) in practice. *Trends Ecol. Evol.* **25**, 410 – 418 (2010). URL http://www.sciencedirect.com/science/article/pii/S0169534710000662.

[17] Bassetti, F., Bodini, A. & Regazzini, E. On minimum kantorovich distance estimators. *Statistics & Probability Letters* **76**, 1298–1302 (2006). URL https://www.sciencedirect.com/science/article/pii/S0167715206000381.

[18] Bernton, E., Jacob, P. E., Gerber, M. & Robert, C. P. Approximate Bayesian Computation with the Wasserstein Distance. *Journal of the Royal Statistical Society Series B: Statistical Methodology* **81**, 235–269 (2019). URL https://doi.org/10.1111/rssb.12312.

[19] Pradella, D. *et al.* Engineered extrachromosomal oncogene amplifications promote tumorigenesis. *Nature* 1–10 (2024). URL https://www.nature.com/articles/s41586-024-08318-8. Publisher: Nature Publishing Group.

[20] Moran, P. A. P. *The statistical processes of evolutionary theory* (Clarendon Press, Oxford, 1962).
