# Supplementary material for: Extrachromosomal DNA–Driven Oncogene Spatial Heterogeneity and Evolution in Glioblastoma
Source: Cancer Discov. 2025 Sep 8;15(10):2078–95. doi: 10.1158/2159-8290.CD-24-1555 (PMC12498097; doi:10.1158/2159-8290.CD-24-1555)
Supplement: Supplementary Figures — 1 to 34, detailing additional analyses. [file cd-24-1555_supplementary_figures_suppsf.pdf]

Supplementary Figures for "Extrachromosomal DNA driven oncogene spatial heterogeneity and evolution in glioblastoma"

Imran Noorani*, Magnus Haughey*, Jens Luebeck, Andrew Rowan, Eva Grönroos, Francesco Terenzi, Ivy Tsz-Lo Wong, Davide Pradella, Marta Lisi, Jeanette Kittel, Natasha Sharma, Chris Bailey, Clare E Weeden, Donald M Bell, Eric Joo, Vittorio Barbè, Matthew G Jones, King L Hung, Emma L Nye, Mary Green, Lucy Meader, Emma J Norton, Mark Fabian, Nnennaya Kanu, Mariam Jamal-Hanjani, Thomas Santarius, Andrea Ventura, James AR Nicoll, Delphine Boche, Howard Y Chang, Vineet Bafna, Weini Huang, Paul S Mischel+, Charles Swanton+, Benjamin Werner+.

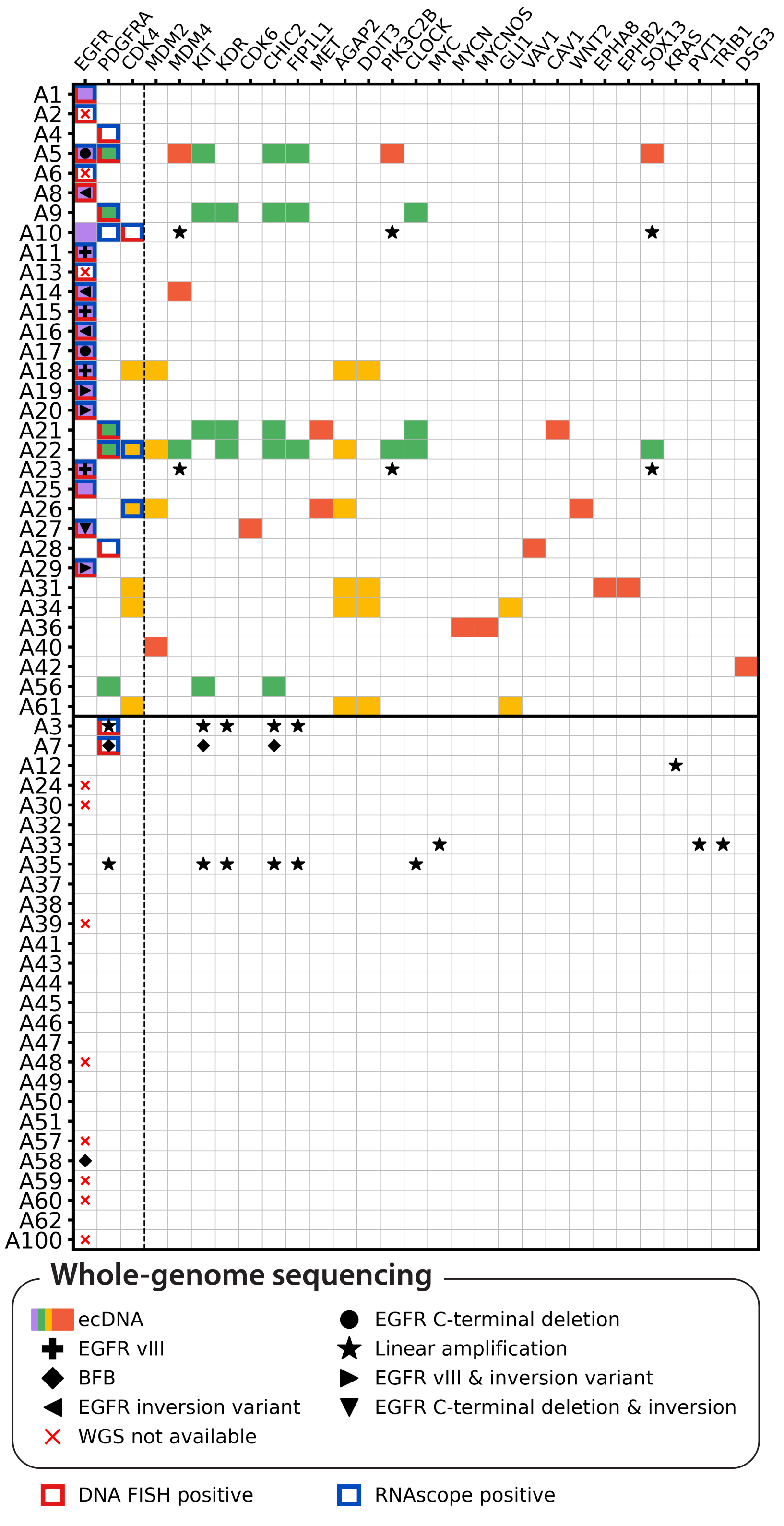

Supplementary Figure 1: Waterfall plot showing focal copy number amplifications across the GB-UK cohort ($n = 59$ patients), categorized by amplicon type (ecDNA, breakage-fusion-bridge (BFB) cycle, linear). Horizontal line between patients A61 & A3 separate patient samples with (above) or without (below) ecDNA, detected either by whole-genome sequencing, DNA FISH or nascent RNAscope. Oncogenes tested with DNA FISH and / or nascent RNAscope, *EGFR*, *PDGFRA* and *CDK4* are separated to the left of the vertical dashed line.

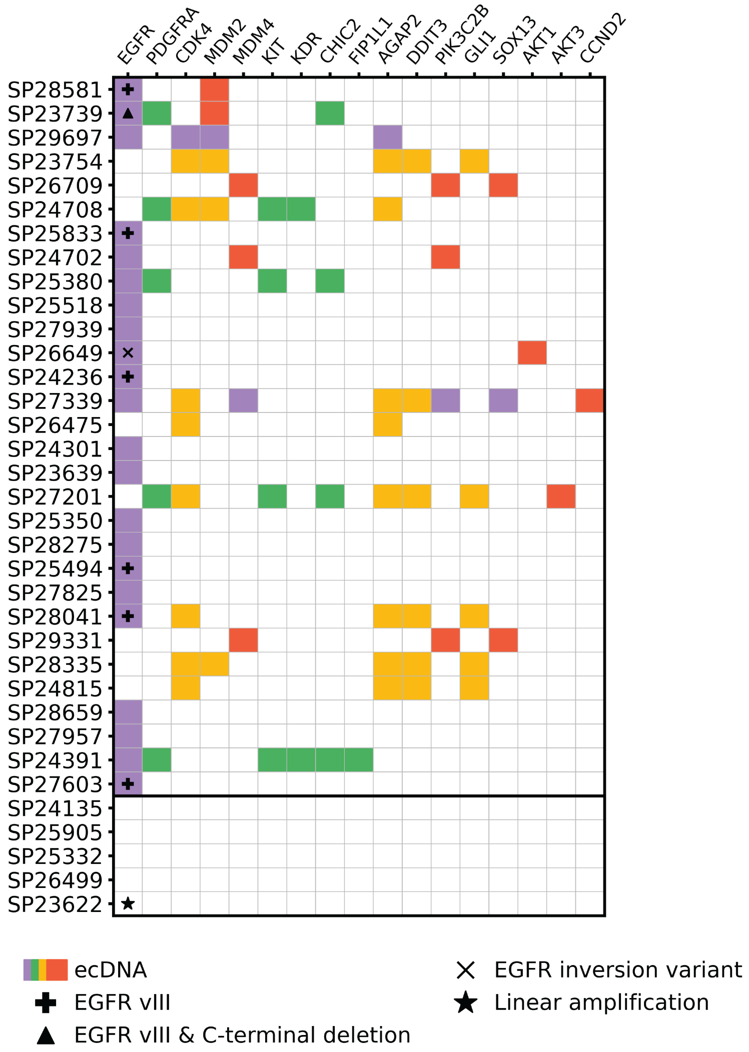

Supplementary Figure 2: Waterfall plot showing focal copy number amplifications across the PCAWG cohort ($n = 35$ patients), categorized by amplicon type (ecDNA, breakage-fusion-bridge (BFB) cycle, linear) Horizontal line between patients SP27603 & SP24135 separate patient samples with (above) or without (below) ecDNA, detected by whole-genome sequencing.

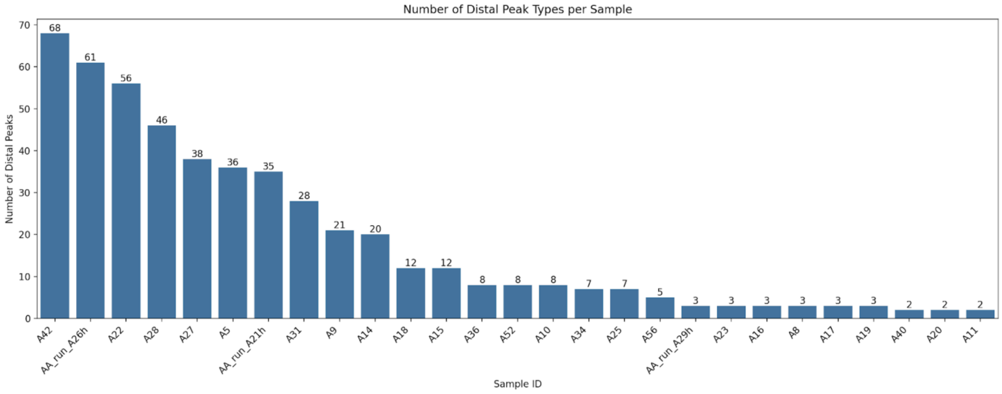

Supplementary Figure 3: Number of enhancer sequences amplified on ecDNA in GB-UK samples.

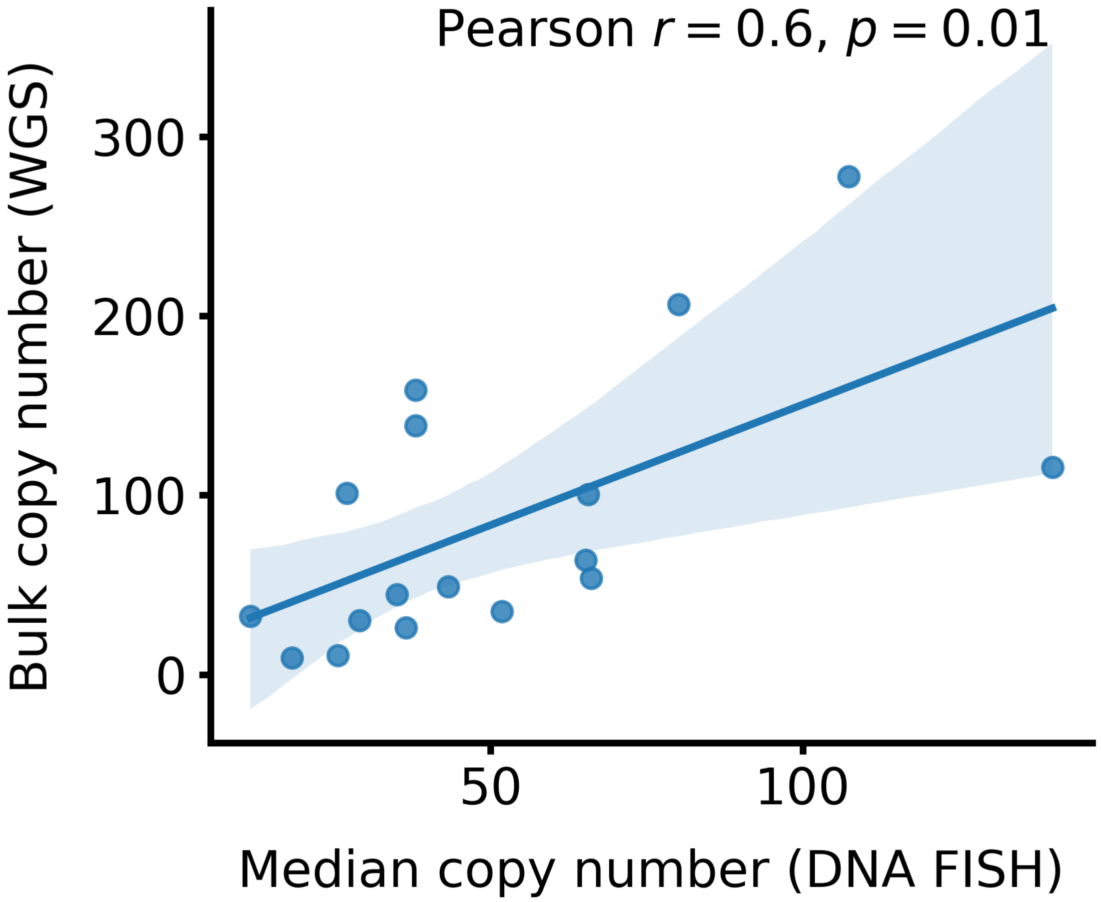

Supplementary Figure 4: Correlation between measured ecDNA oncogene copy numbers, measured using DNA FISH and whole-genome sequencing.

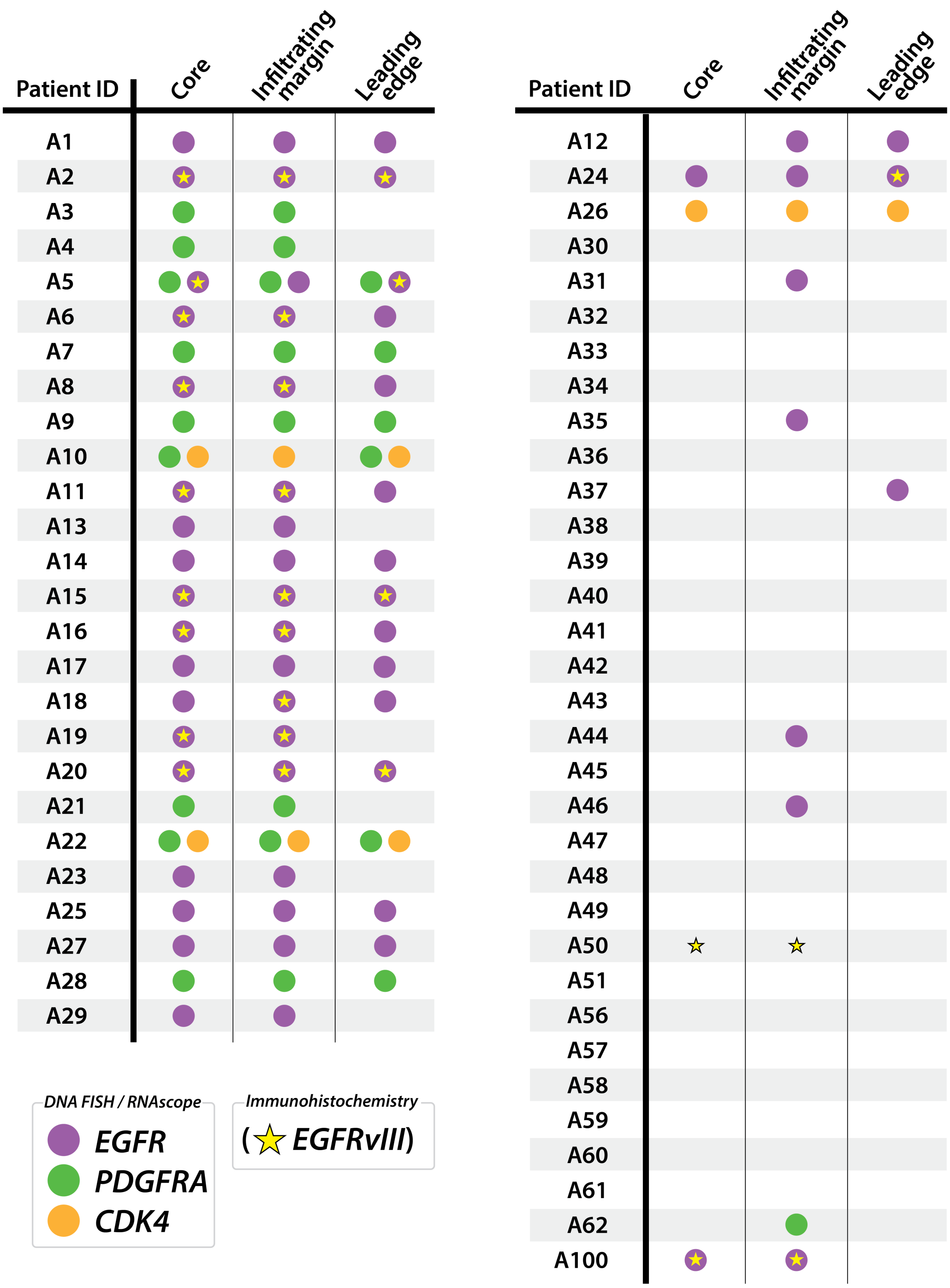

Supplementary Figure 5: Oncogenic ecDNA observations in tumor core, infiltrating margin and leading edge locations across entire patient cohort. Data represent combined DNA FISH, RNAscope and immunohistochemistry observations

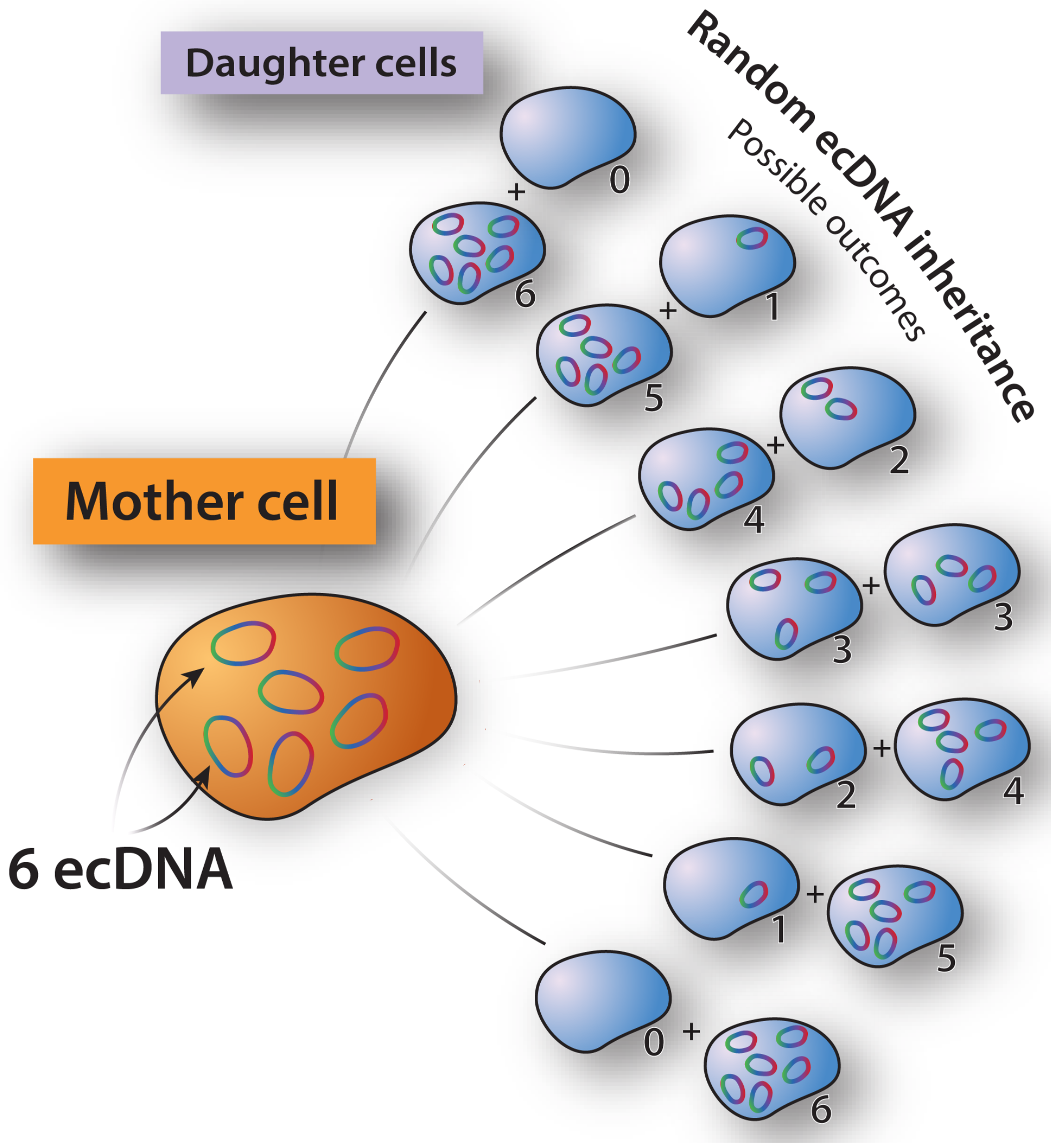

Supplementary Figure 6: During cell division, following replication of ecDNA, these molecules are divided binomially among daughter cells.

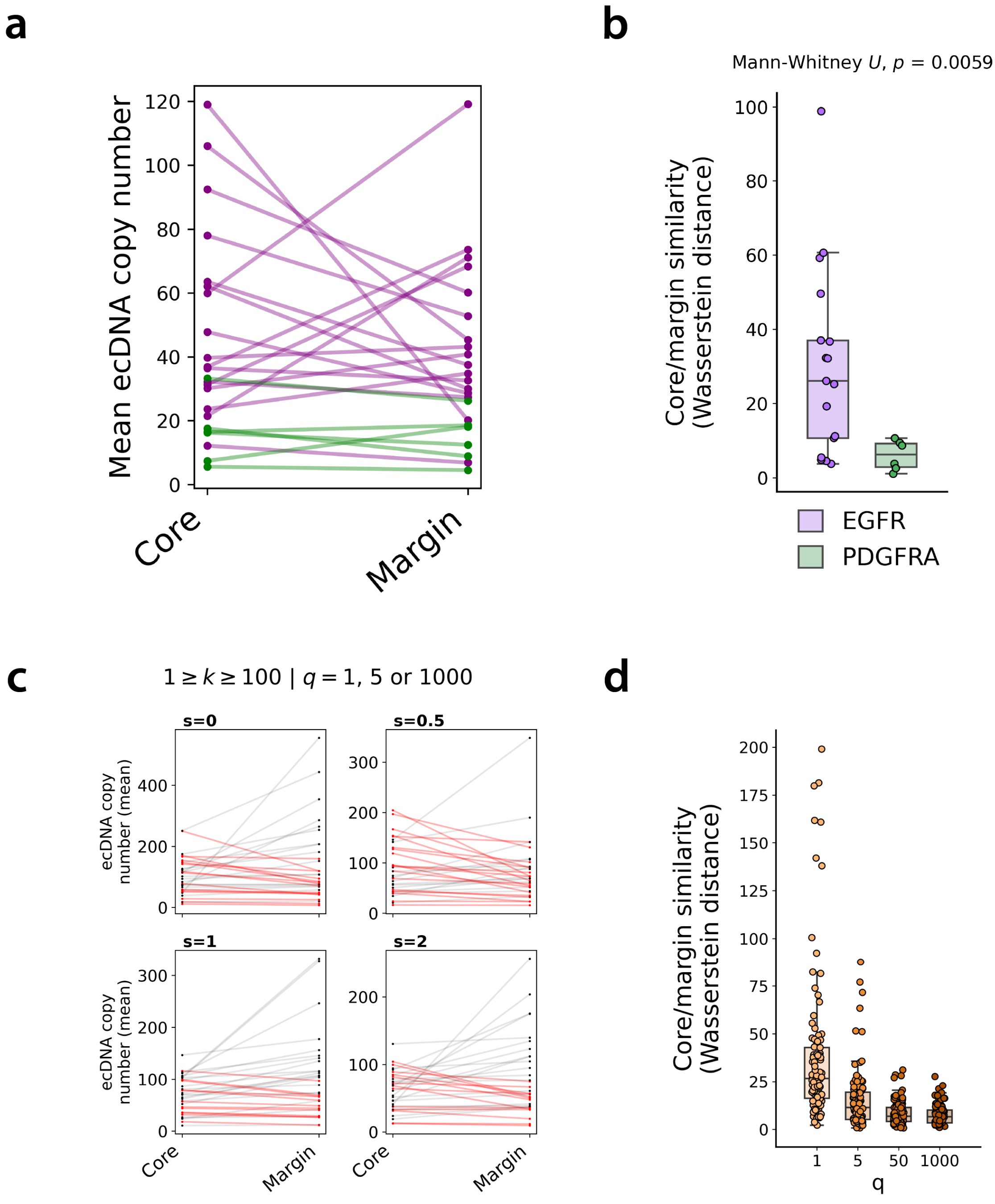

Supplementary Figure 7: **(a)** Mean ecDNA copy number across core & margin regions of GB-UK patients. Line color corresponds to ecDNA-amplified oncogene (Purple = *EGFR*, Green = *PDGFRA*). **(b)** Core and margin ecDNA copy number distribution similarity, measured using Wasserstein distance for GB-UK patients. **(c)** Mean and variance ecDNA copy number across core and randomly located margin regions of simulated tumors over a range of ecDNA initial ecDNA copy numbers, k, and selection strengths, s and cell pushing strengths, q. Sampled core and margin regions comprised 1,000 cells. Colored lines (red) represent cases where the mean ecDNA copy number was smaller in the margin sample compared to the core. **(d)** Core and margin ecDNA copy number distribution similarity, measured using Wasserstein distance for simulated tumors over a range of q values ($s = 1$, $k = 50$).

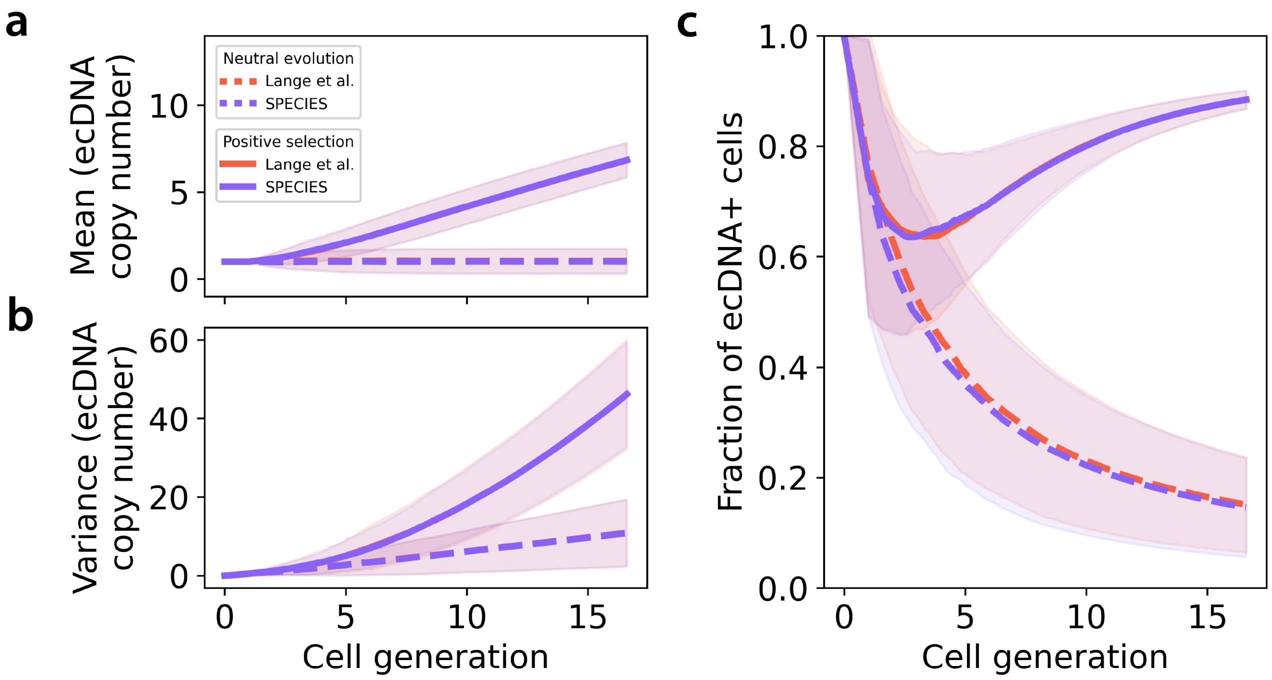

Supplementary Figure 8: Benchmarking predictions of the **(a)** mean and **(b)** variance of the ecDNA copy number distribution, and **(c)** the fraction of ecDNA+ cells over time by SPECIES model ($k = 1$ & q =1,000) against previous non-spatial ecDNA evolutionary model of Lange *et al.* (Lange *et al. Nat Genet* **54**, 1527–1533 (2022) (https://doi.org/10.1038/s41588-022-01177-x)) both for neutral ($s = 0$) and positive ($s = 3$) ecDNA-conferred selection.

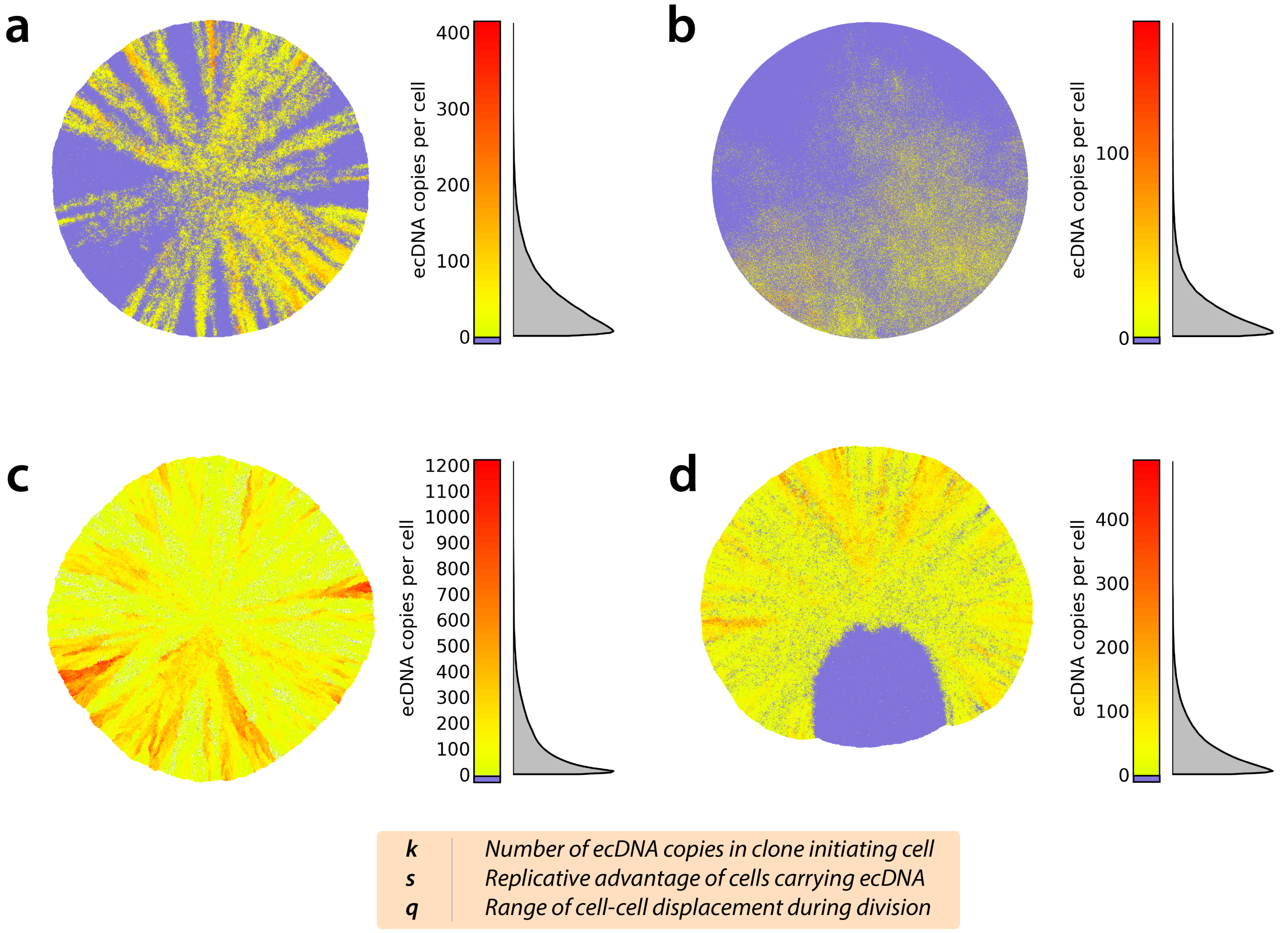

Supplementary Figure 9: Example ecDNA-driven tumors simulated with SPECIES for **(a)** k = 16, s = 0, q = 10; **(b)** k = 15, s = 0, q =1,000; **(c)** k = 105, s = 4.9, q = 2 and **(d)** k = 1, s = 0.2, q = 10, showing typical range of simulated tumor ecDNA patterns attainable with SPECIES.

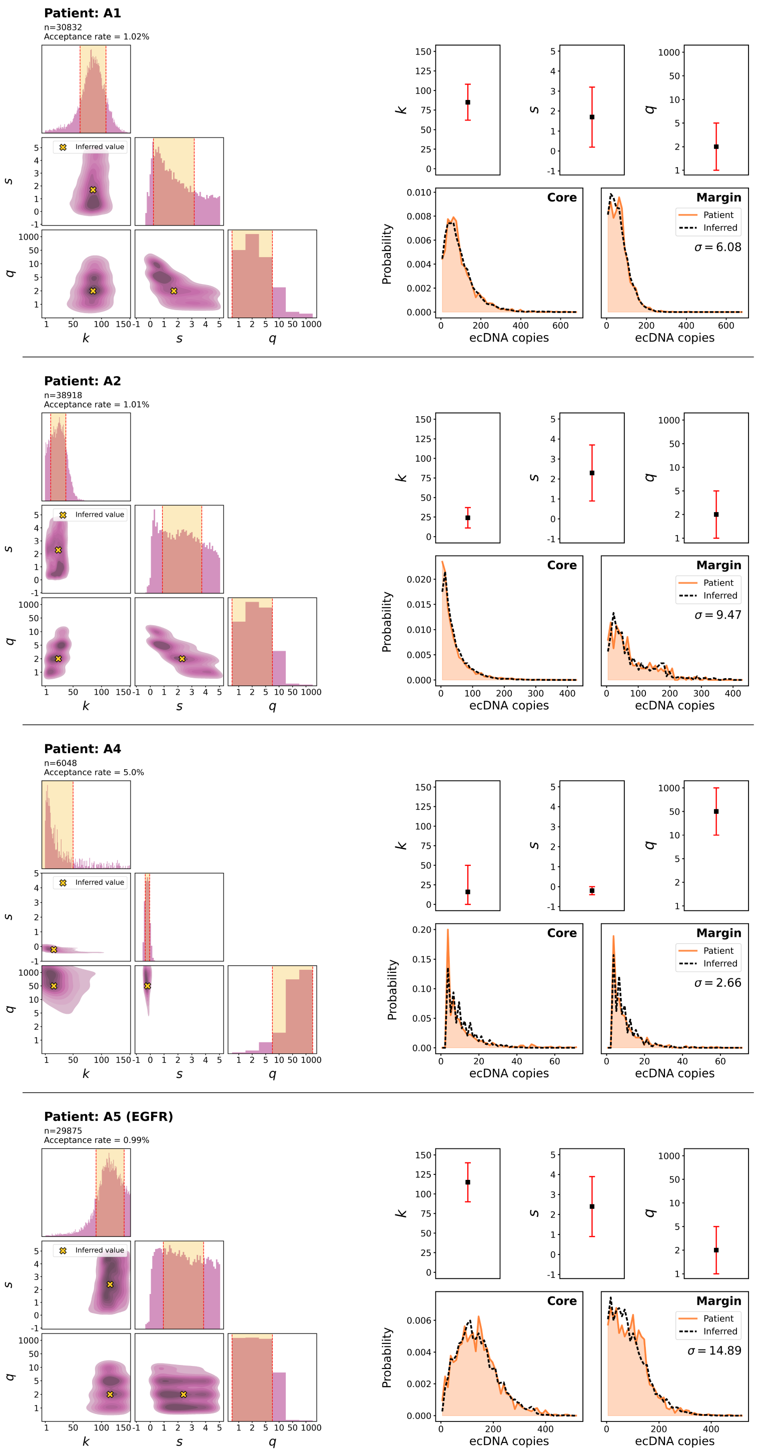

Supplementary Figure 10: *(Left)* Posterior parameter distributions for patients A1 to A5 (*EGFR*). Diagonal panels show 1D marginal distributions for k, s and q. Off-diagonal show 2D marginal distributions for each combination of parameters. *(Right)* Summary of inferred k, s & q (top row) and patient-derived single-cell ecDNA copy number distributions, determined using DNA FISH, with corresponding best-fit distributions from simulated tumors (bottom row). Sum of Wasserstein distance between patient and simulated distributions for tumor core and infiltrating margin, representing closeness of fit, is denoted by σ.

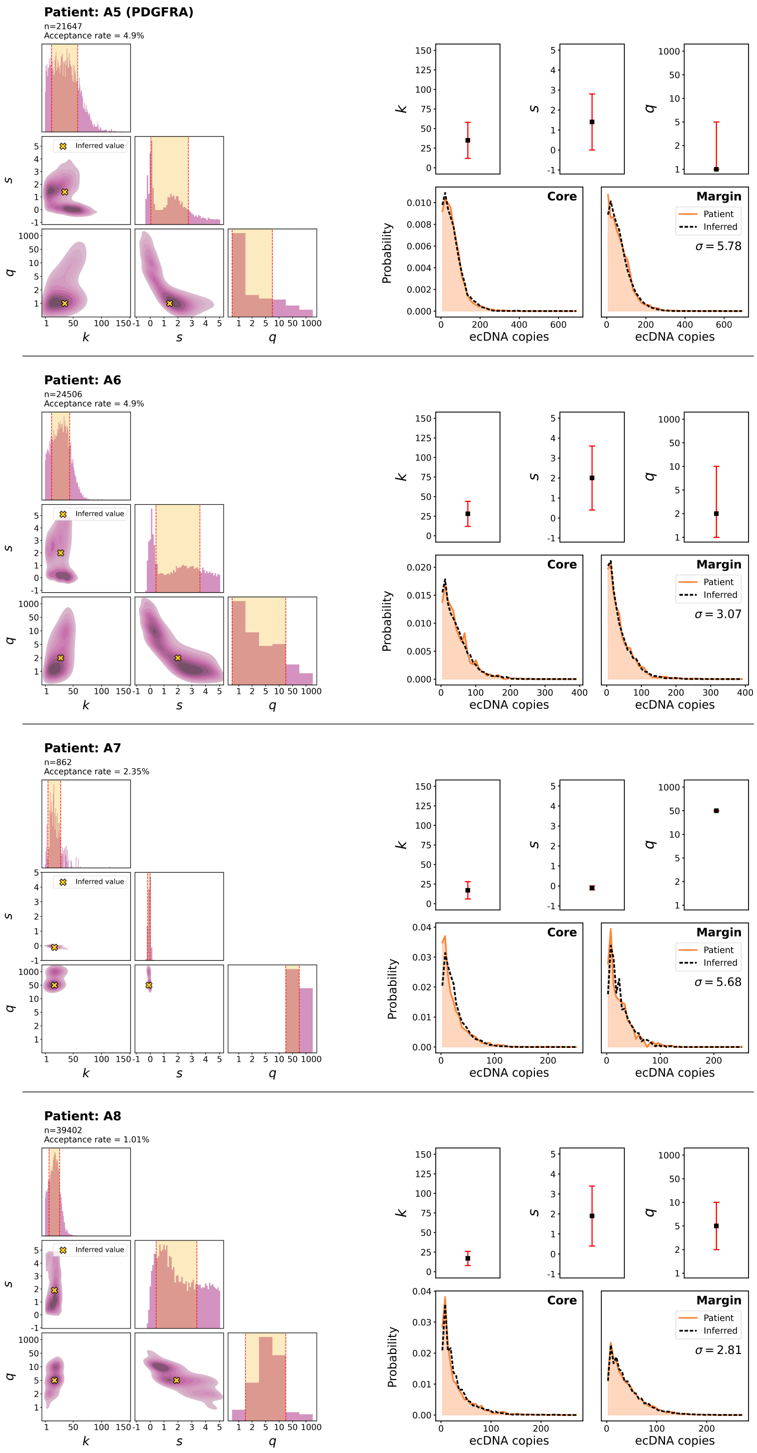

Supplementary Figure 11: *(Left)* Posterior parameter distributions for patients A5 (*PDGFRA*) to A8. Diagonal panels show 1D marginal distributions for k, s and q. Off-diagonal show 2D marginal distributions for each combination of parameters. n and acceptance rate denote the absolute and percentage number of simulations accepted into the posterior parameter set, respectively. *(Right)* Summary of inferred k, s & q (top row) and patient-derived single-cell ecDNA copy number distributions, determined using DNA FISH, with corresponding best-fit distributions from simulated tumors (bottom row). Sum of Wasserstein distance between patient and simulated distributions for tumor core and infiltrating margin, representing closeness of fit, is denoted by σ.

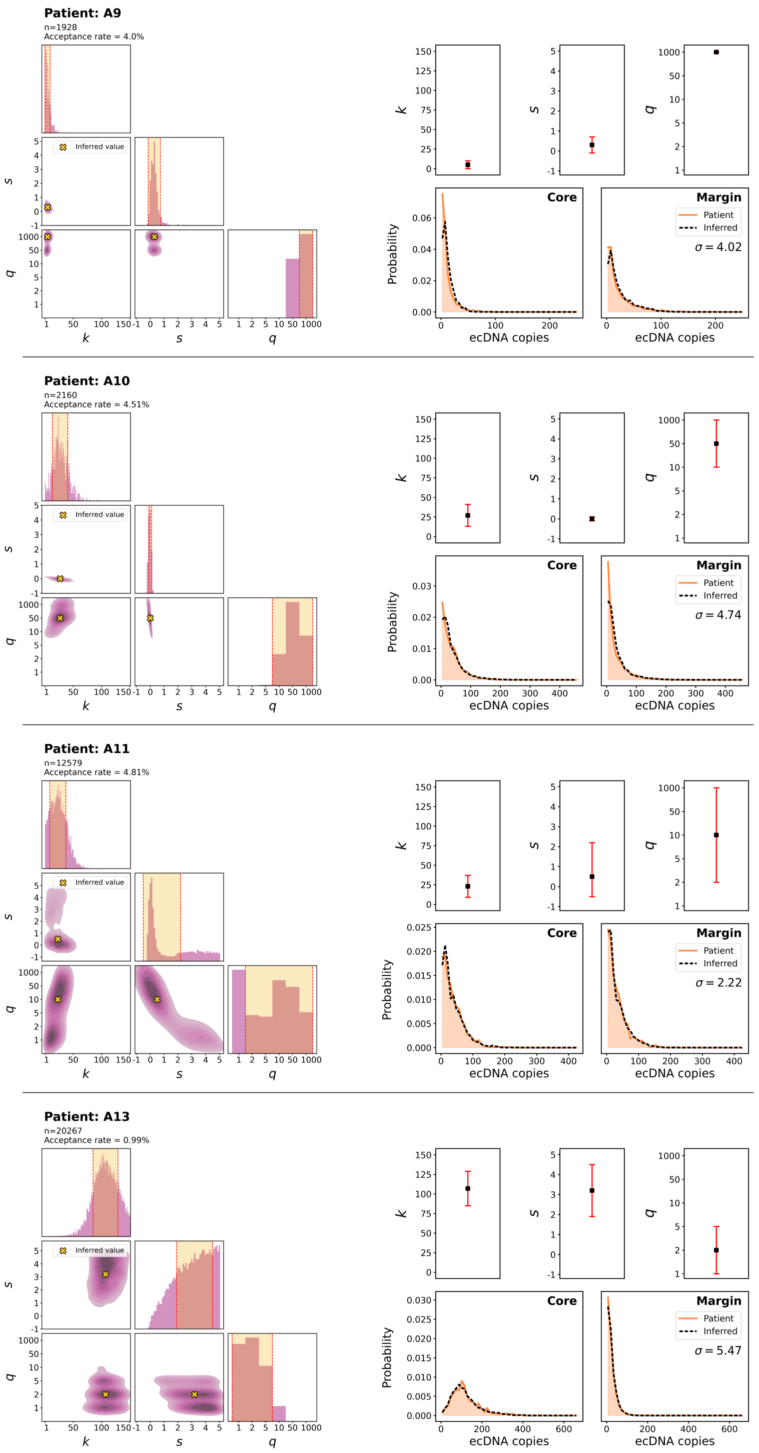

Supplementary Figure 12: *(Left)* Posterior parameter distributions for patients A9 to A13. Diagonal panels show 1D marginal distributions for k, s and q. Off-diagonal show 2D marginal distributions for each combination of parameters. n and acceptance rate denote the absolute and percentage number of simulations accepted into the posterior parameter set, respectively. *(Right)* Summary of inferred k, s & q (top row) and patient-derived single-cell ecDNA copy number distributions, determined using DNA FISH, with corresponding best-fit distributions from simulated tumors (bottom row). Sum of Wasserstein distance between patient and simulated distributions for tumor core and infiltrating margin, representing closeness of fit, is denoted by σ.

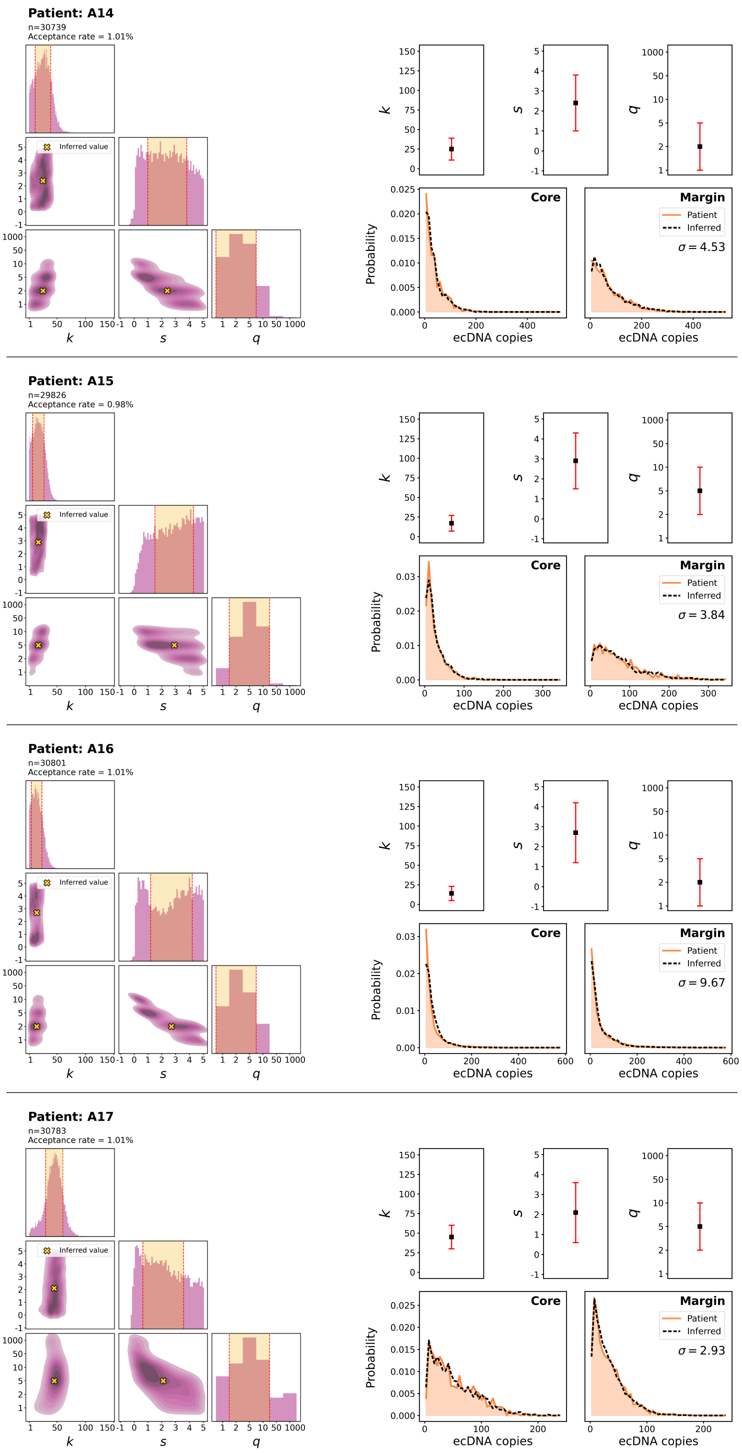

Supplementary Figure 13: *(Left)* Posterior parameter distributions for patients A14 to A17. Diagonal panels show 1D marginal distributions for k, s and q. Off-diagonal show 2D marginal distributions for each combination of parameters. n and acceptance rate denote the absolute and percentage number of simulations accepted into the posterior parameter set, respectively. *(Right)* Summary of inferred k, s & q (top row) and patient-derived single-cell ecDNA copy number distributions, determined using DNA FISH, with corresponding best-fit distributions from simulated tumors (bottom row). Sum of Wasserstein distance between patient and simulated distributions for tumor core and infiltrating margin, representing closeness of fit, is denoted by σ.

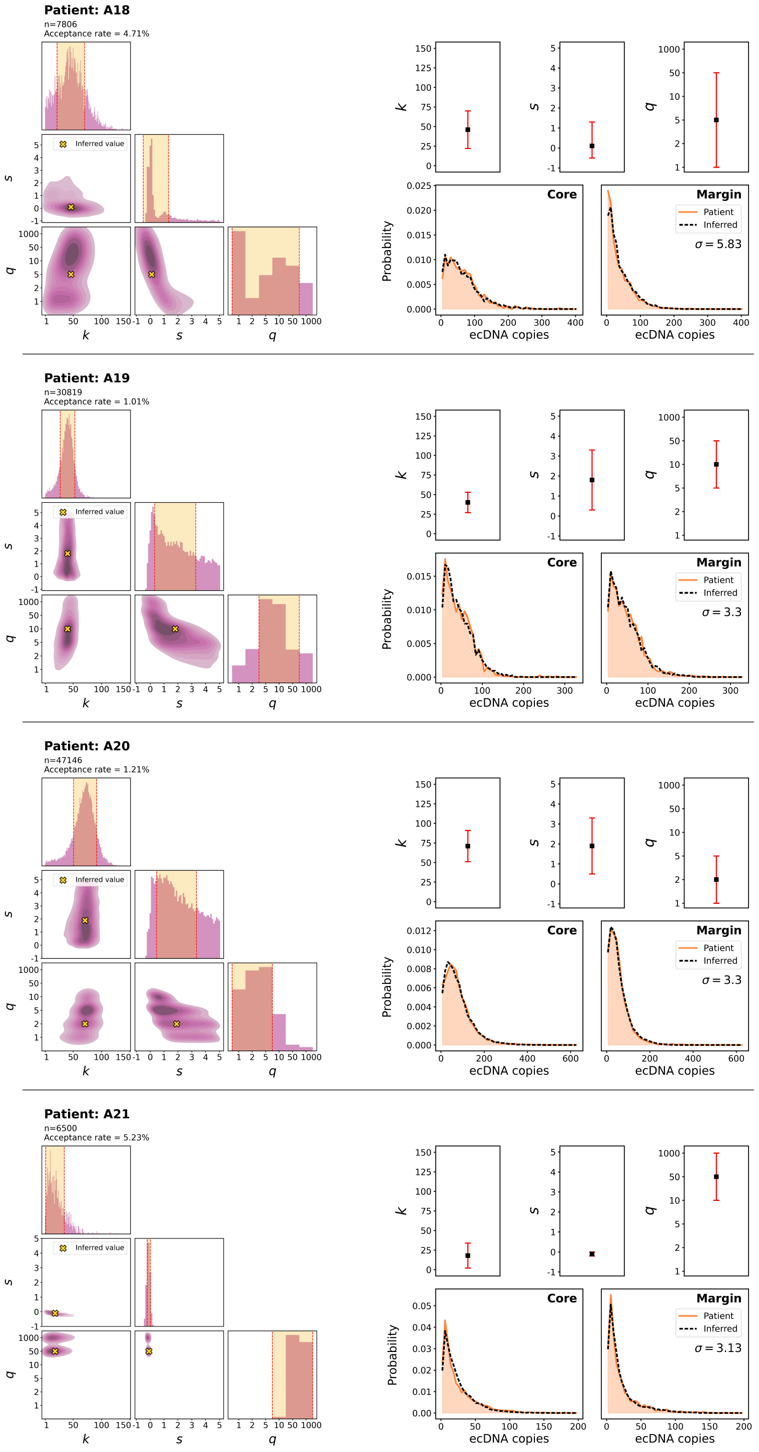

Supplementary Figure 14: *(Left)* Posterior parameter distributions for patients A18 to A21. Diagonal panels show 1D marginal distributions for k, s and q. Off-diagonal show 2D marginal distributions for each combination of parameters. n and acceptance rate denote the absolute and percentage number of simulations accepted into the posterior parameter set, respectively. *(Right)* Summary of inferred k, s & q (top row) and patient-derived single-cell ecDNA copy number distributions, determined using DNA FISH, with corresponding best-fit distributions from simulated tumors (bottom row). Sum of Wasserstein distance between patient and simulated distributions for tumor core and infiltrating margin, representing closeness of fit, is denoted by σ.

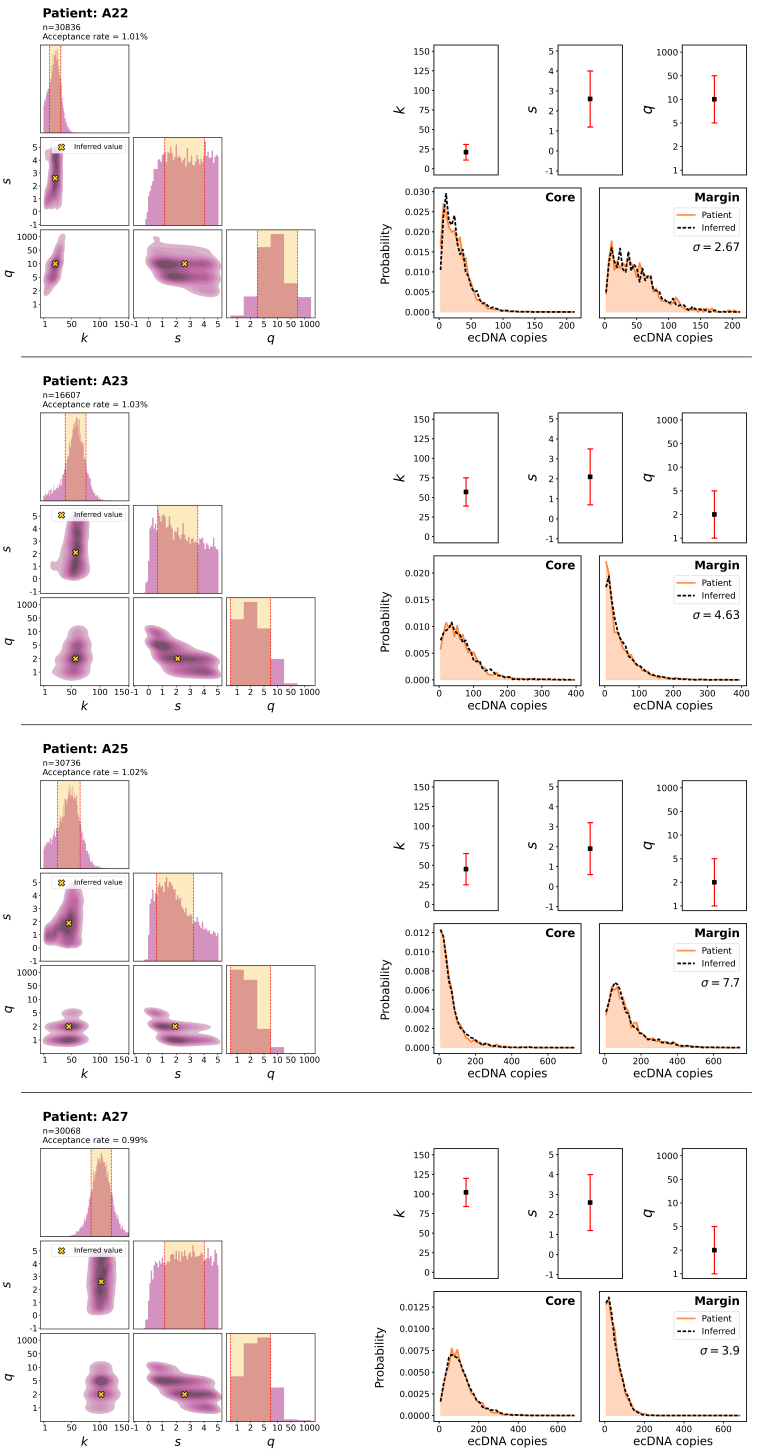

Supplementary Figure 15: *(Left)* Posterior parameter distributions for patients A22 to A27. Diagonal panels show 1D marginal distributions for k, s and q. Off-diagonal show 2D marginal distributions for each combination of parameters. n and acceptance rate denote the absolute and percentage number of simulations accepted into the posterior parameter set, respectively. *(Right)* Summary of inferred k, s & q (top row) and patient-derived single-cell ecDNA copy number distributions, determined using DNA FISH, with corresponding best-fit distributions from simulated tumors (bottom row). Sum of Wasserstein distance between patient and simulated distributions for tumor core and infiltrating margin, representing closeness of fit, is denoted by σ.

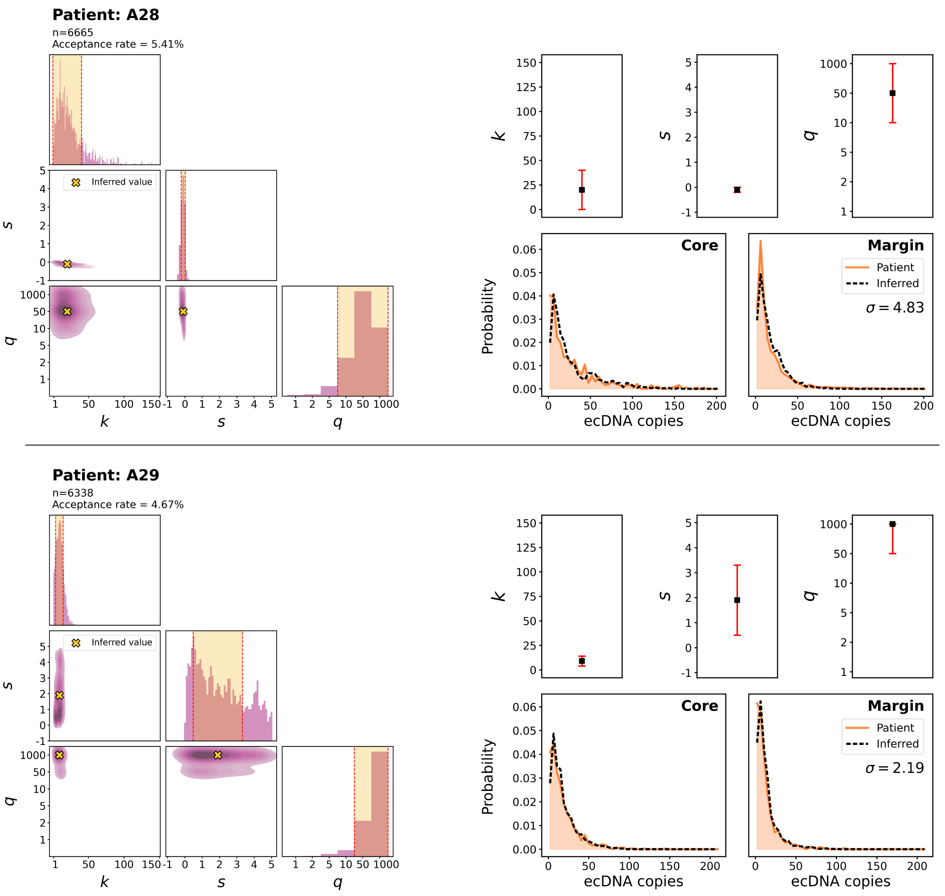

Supplementary Figure 16: *(Left)* Posterior parameter distributions for patients A28 and A29. Diagonal panels show 1D marginal distributions for k, s and q. Off-diagonal show 2D marginal distributions for each combination of parameters. n and acceptance rate denote the absolute and percentage number of simulations accepted into the posterior parameter set, respectively. *(Right)* Summary of inferred k, s & q (top row) and patient-derived single-cell ecDNA copy number distributions, determined using DNA FISH, with corresponding best-fit distributions from simulated tumors (bottom row). Sum of Wasserstein distance between patient and simulated distributions for tumor core and infiltrating margin, representing closeness of fit, is denoted by σ.

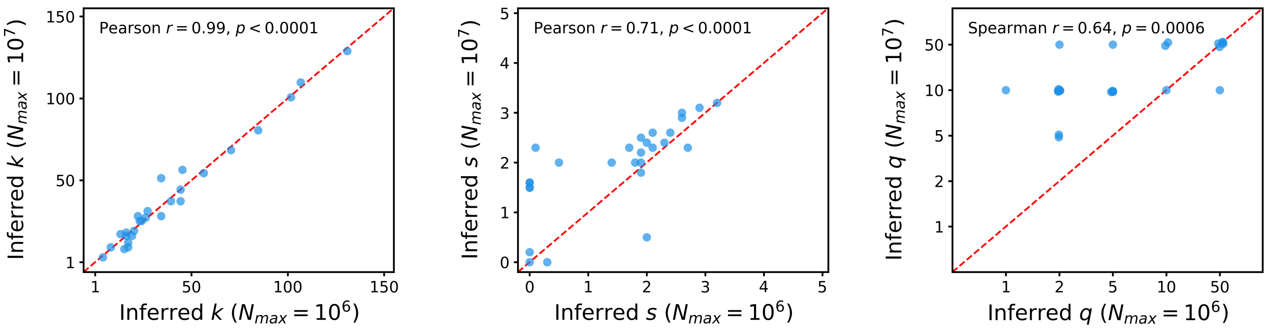

Supplementary Figure 17: Comparison of inferred values of k, s and q for GB-UK patients using the simulated maximum tumor size of $N_{max} = 10^6$ versus $N_{max} = 10^7$.

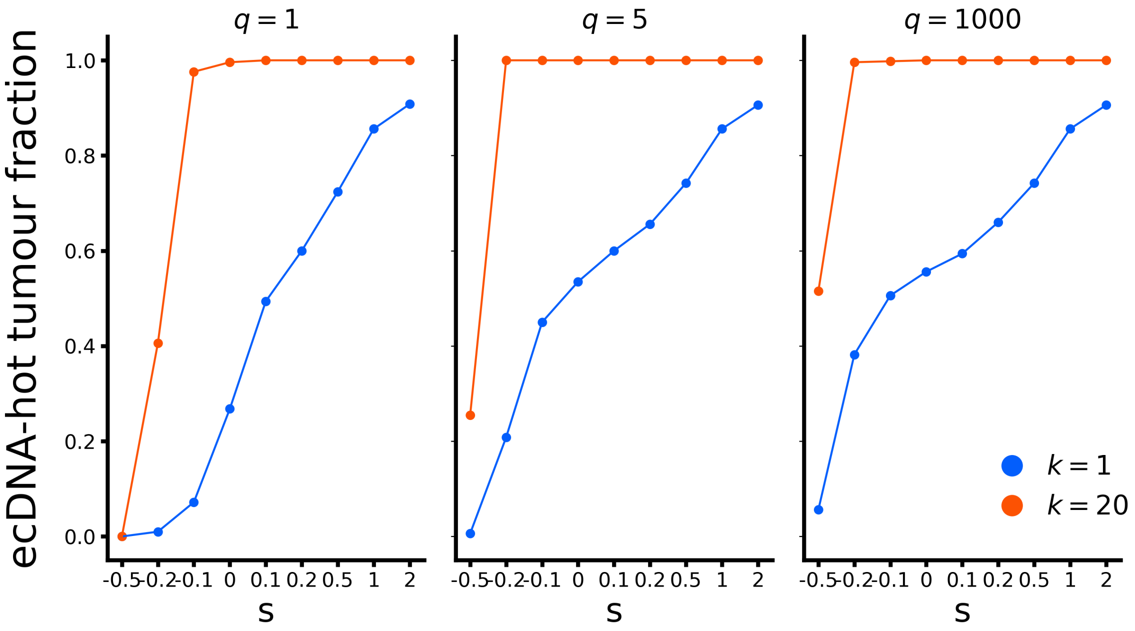

Supplementary Figure 18: Fraction of simulated tumors which maintain ecDNA within at least one tumor cell (termed ecDNA-hot tumors) measured at a final tumor size of $N_{max} = 10^5$ cells. Data represent mean values for 500 independent realizations of the stochastic simulation.

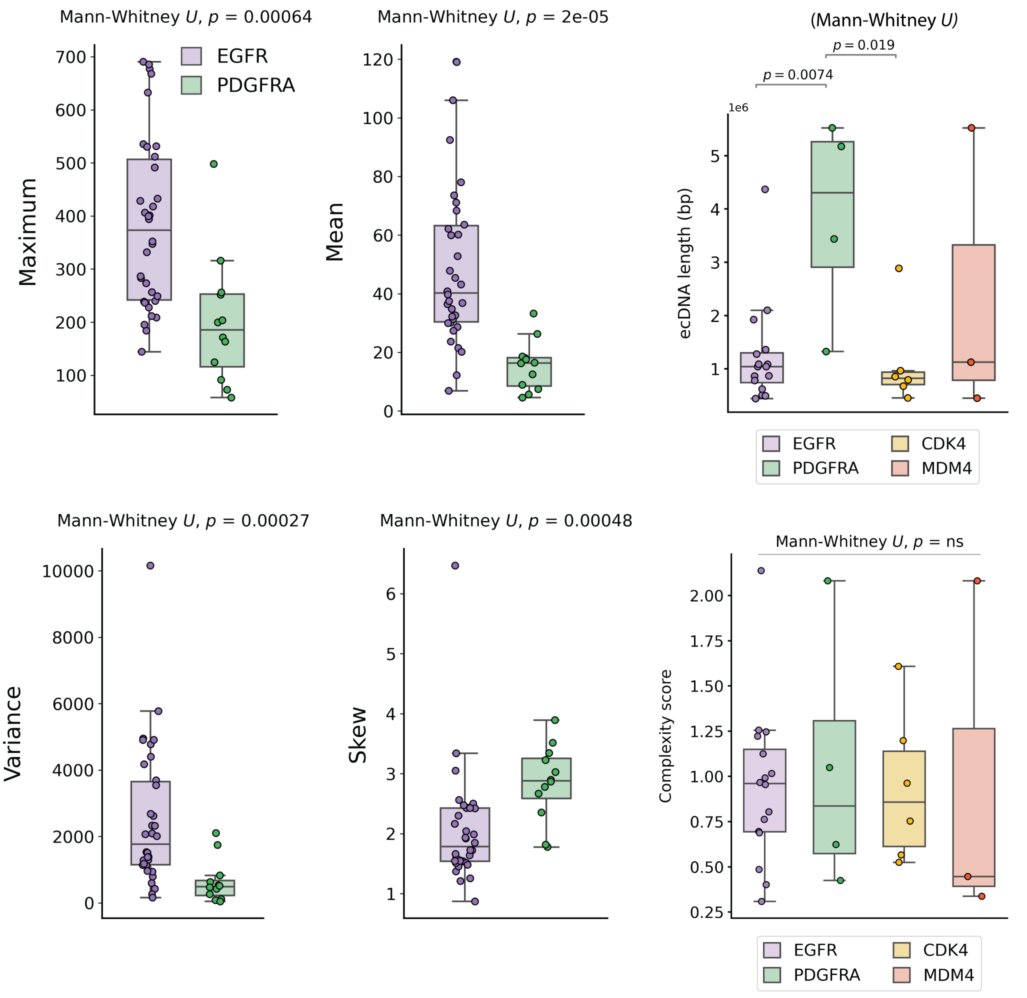

Supplementary Figure 19: Oncogene-level differences in observed ecDNA copy number distributions in GB-UK patient tumors.

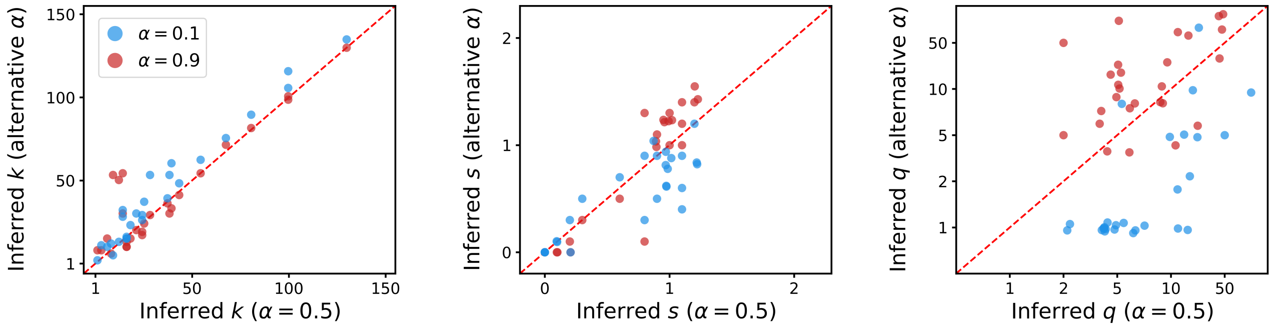

Supplementary Figure 20: Comparison of predicted model parameters when using a cell death rate of $\alpha = 0.5$ to alternative models using low ($\alpha = 0.1$) and high ($\alpha = 0.9$) death rates. Cell death rate, r_d, is computed as $r_d = r_b(x, 0) \cdot \alpha$, where $r_b(x, s = 0)$ denotes the neutral birth rate of a cell with x copies of ecDNA.

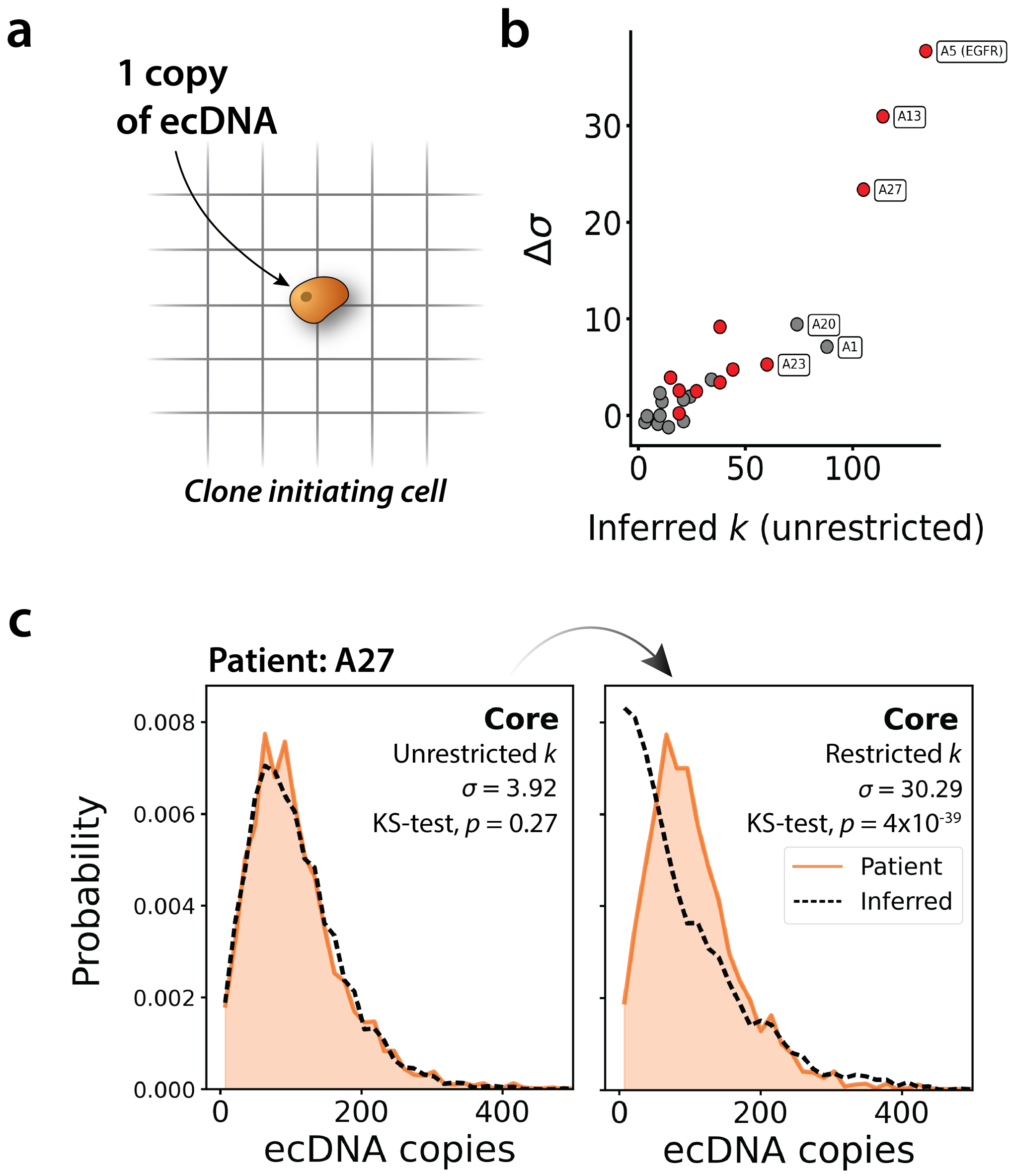

Supplementary Figure 21: **(a)** Parameter inference was repeated with a modified computational model, in which the initial tumor cell ecDNA copy number was restricted to $k = 1$. **(b)** ABC fit quality difference, $\Delta\sigma$, which compares inference with unrestricted k value (Bayesian prior $P(k) = \mathrm{U}(1{,}150)$) to restricted case of $k = 1$. Larger values of $\Delta\sigma$ indicate a worse fit when k is restricted. Red colored points denote patients for which the best-fit model data was an exact fit to the patient data in the unrestricted k case (core sample, Kolmogorov-Smirnov (KS) test, $p > 0.05$), but not in the restricted k case (core sample, KS test, $p \leq 0.05$). **(c)** Example of poor model best-fit for patient A27 when k is restricted to $k = 1$.

	Sample	chromosome	position 1	position 2	sv_type	read support	amplicon type	spanning exons	vIII equivalent	c-terminal deletion
GB-UK	A5	chr7	55200682	55205057	deletion-like	58	ecDNA	25-27		Y
	A8	chr7	54920809	55154753	inversion	28	ecDNA	1-7	Y	
	A11	chr7	55132062	55155691	deletion-like	88	ecDNA	2-7	Y	
	A14	chr7	55119613	55154385	inversion	80	ecDNA	2-7	Y	
	A15	chr7	55118911	55155705	deletion-like	66	ecDNA	2-7	Y	
	A16	chr7	55021945	55179866	inversion	19	ecDNA	1-19		
		chr7	55009553	55154336	inversion	23	ecDNA	1-7	Y	
	A17	chr7	55200774	55204714	deletion-like	38	ecDNA	25-27		Y
	A19	chr7	55142134	55154633	deletion-like	36	ecDNA	2-7	Y	
		chr7	54843745	55128844	inversion	20	ecDNA	1		
		chr7	54880847	55154597	inversion	21	ecDNA	1-7	Y	
		chr7	54884387	55141732	inversion	17	ecDNA	1		
	A20	chr7	55025248	55155160	deletion-like	4	ecDNA	2-7	Y	
		chr7	55122733	55154336	deletion-like	4	ecDNA	2-7	Y	
		chr7	55123695	55155612	deletion-like	6	ecDNA	2-7	Y	
	A23	chr7	55026802	55155149	deletion-like	5	ecDNA	2-7	Y	
		chr7	55132447	55153405	foldback	2	ecDNA	2-6		
	A27	chr7	55201858	56259403	inversion	7	ecDNA	27,28+		Y
		chr7	55202026	55742391	deletion-like	10	ecDNA	27,28+		Y
	A29	chr7	55093609	55155808	deletion-like	17	ecDNA	2-7	Y	
PCAWG	DO12952-SP27603	chr7	55097880	55223306	deletion-like	54	ecDNA	2-7	Y	
	DO11238-SP24301	chr7	55199311	55222950	deletion-like	45	ecDNA	2-7	Y	
		chr7	55199544	55222330	deletion-like	309	ecDNA	2-7	Y	
	DO10960-SP23739	chr7	55122639	55223386	deletion-like	24	ecDNA	2-7	Y	
		chr7	55130764	55223089	deletion-like	24	ecDNA	2-7	Y	
		chr7	55268961	55274292	deletion-like	15	ecDNA	25-28		Y
	DO12034-SP25833	chr7	55105714	55221814	deletion-like	410	ecDNA	2-7	Y	
	DO13192-SP28041	chr7	55191873	55223395	deletion-like	86	ecDNA	2-7	Y	
		chr7	55201724	55222811	deletion-like	131	ecDNA	2-7	Y	
	DO12454-SP26649	chr7	54958584	55223150	inversion	106	ecDNA	2-7		
	DO11202-SP24236	chr7	55150598	55221850	deletion-like	53	ecDNA	2-7	Y	
	DO13474-SP28581	chr7	55198927	55222711	deletion-like	4	ecDNA	2-7	Y	
	DO11854-SP25494	chr7	55146310	55222238	deletion-like	57	ecDNA	2-7	Y	

Supplementary Figure 22: Structural variant analysis of *EGFR*-ecDNA in GB-UK and PCAWG GBM samples.

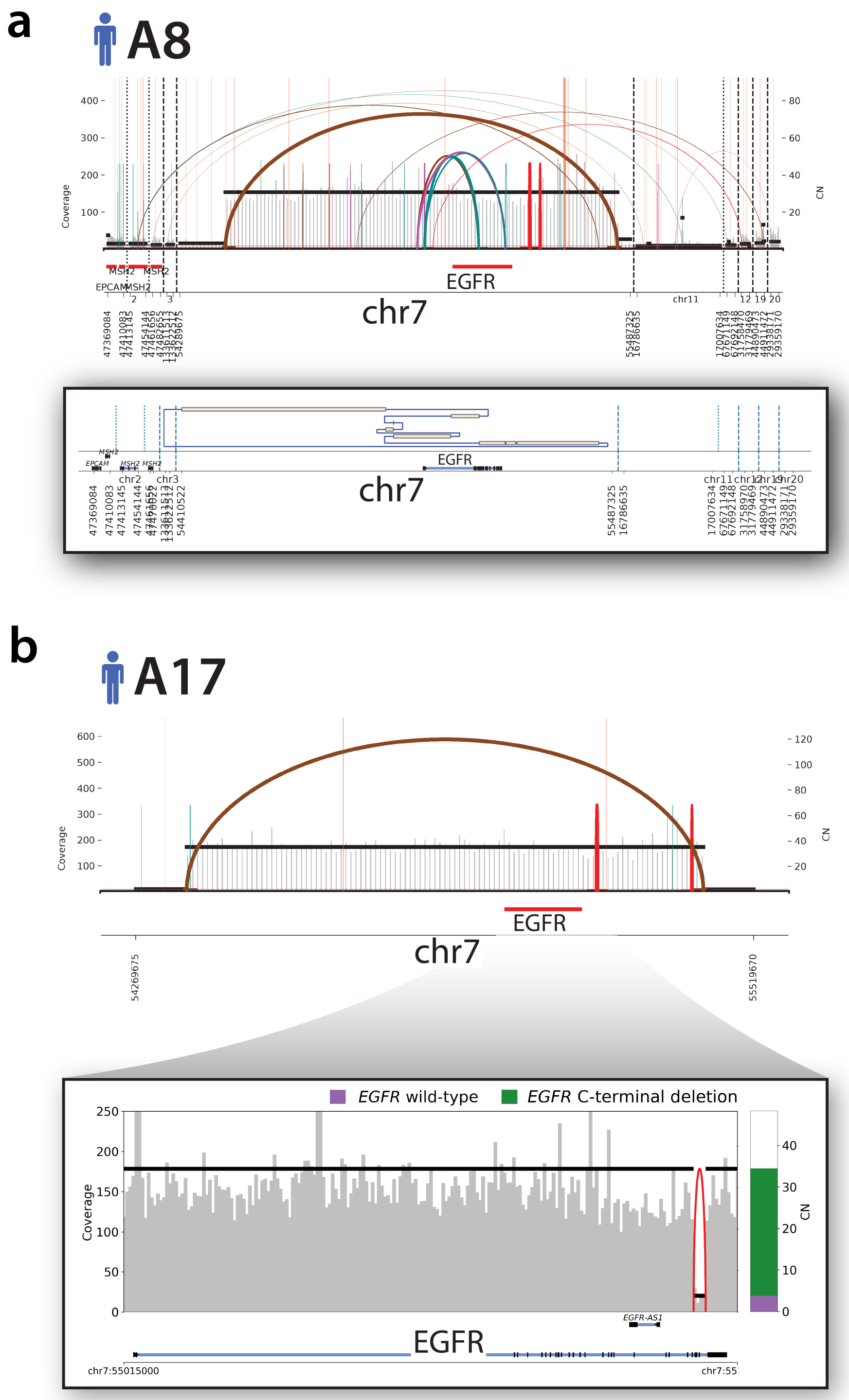

Supplementary Figure 23: Representative structural variants in *EGFR*-ecDNA. **(a)** Upper panel is a Sashimi plot highlighting the structural variants and relative copy number of *EGFR*-ecDNA in patient A8. Lower panel is a joining plot showing a translocation of EGFR exons 1 – 7 to upstream of *EGFR* in an inverted orientation. **(b)** Upper panel is a Sashimi plot highlighting a simple circular *EGFR*-ecDNA in patient A17. Lower panel is a zoomed in view of *EGFR* showing a deep c-terminal deletion within the ecDNA.

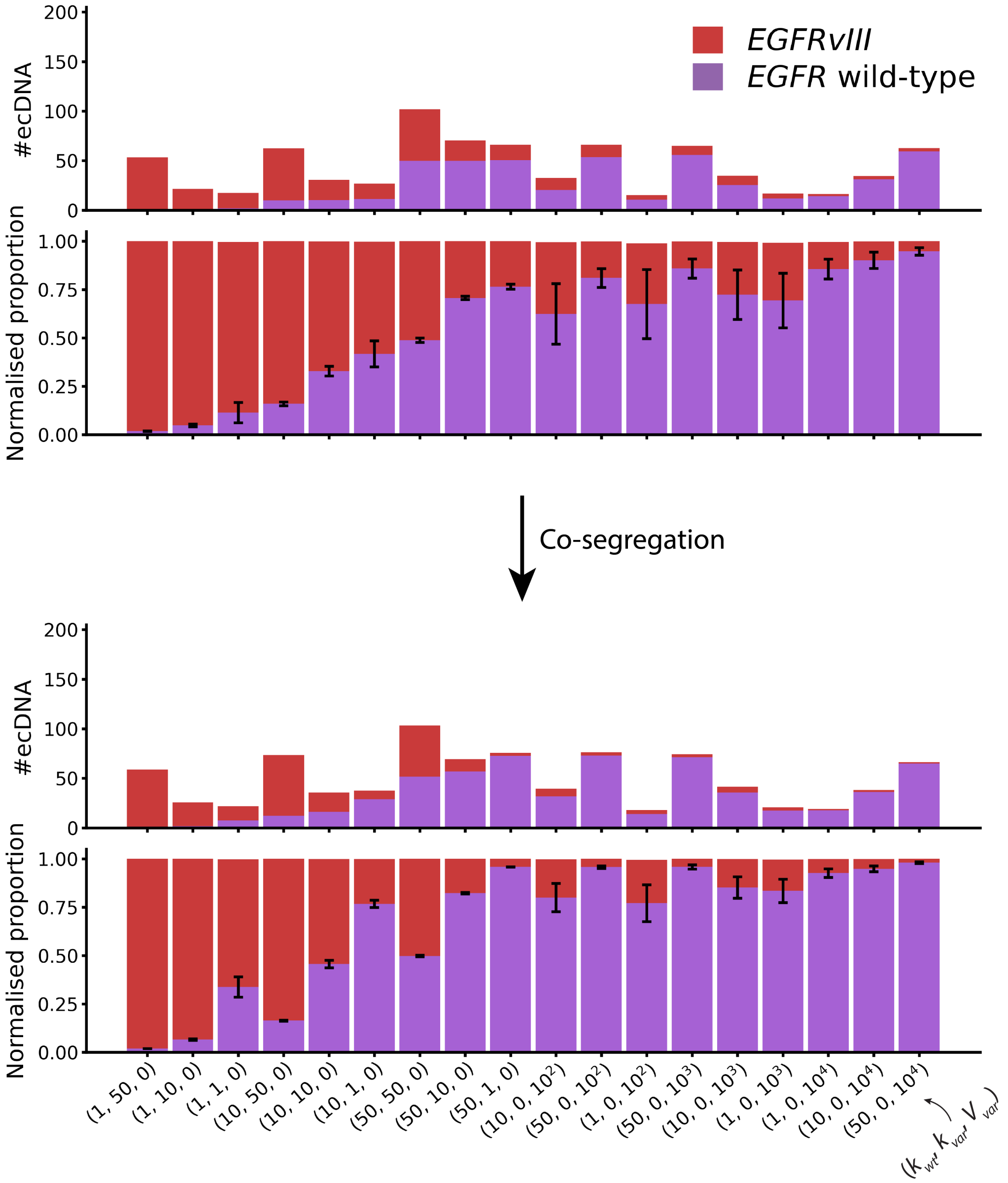

Supplementary Figure 24: Model predictions for resulting variant heteroplasmy with and without co-segregating ecDNAs in tumor for a range of wild-type and variant bearing ecDNA copy numbers in the clone-initiating tumor cell (for pre-expansion *EGFR* mutation) and mutation times (for post-expansion *EGFR* mutation). Simulations were performed with a fixed value of $q = 2$, a co-segregation parameter of 0.6 and selection coefficients for wild-type and variant ecDNA of $s_{wt} = 0.2$ and $s_{var} = 2.0$ respectively. Mean ± variance derived from 1,000 simulated tumors.

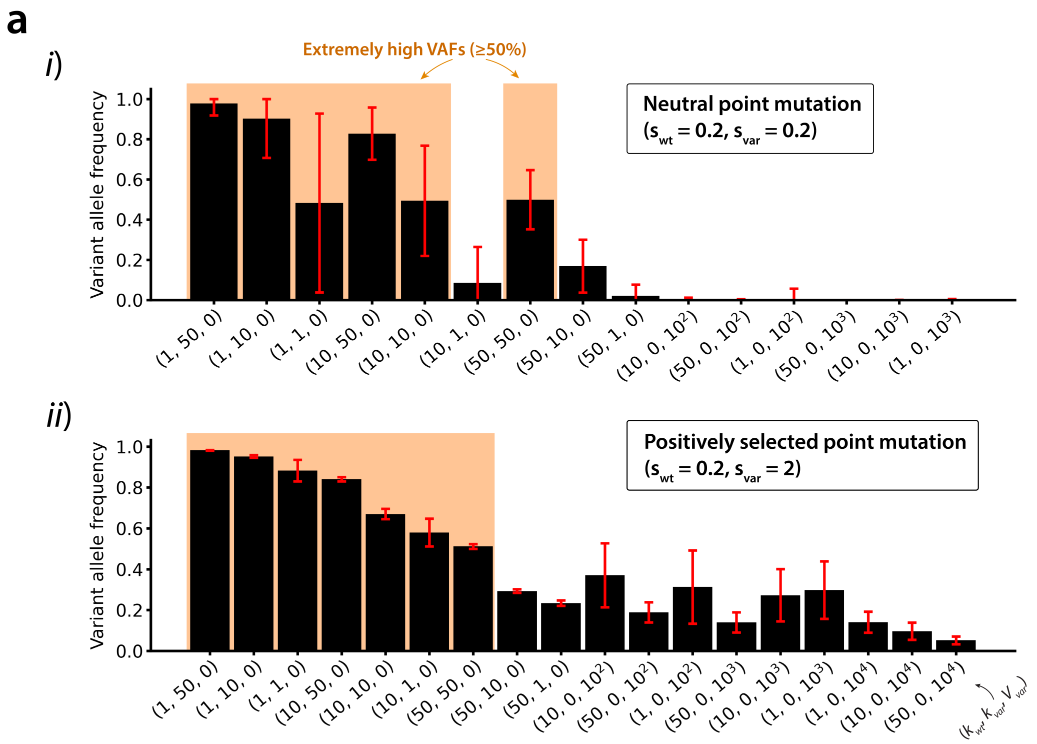

Supplementary Figure 25: SPECIES simulations of expansion of point mutations on ecDNA. **(a)** Simulated frequencies (i) neutral and (ii) positively selected point mutations for a range of mutation event times and initial frequencies in the tumor-initiating cell. Simulation parameters across horizontal axis represent *EGFR* wild-type and variant ecDNA copy number (k_{wt} and k_{var} respectively) in the tumor-initiating cell. The mutation event occurs after V_{var} cell divisions, thus $V_{var} = 0$ corresponds to a pre-existing mutant ecDNA and $V_{var} > 0$ corresponds to a mutation event after the onset of clonal expansion.

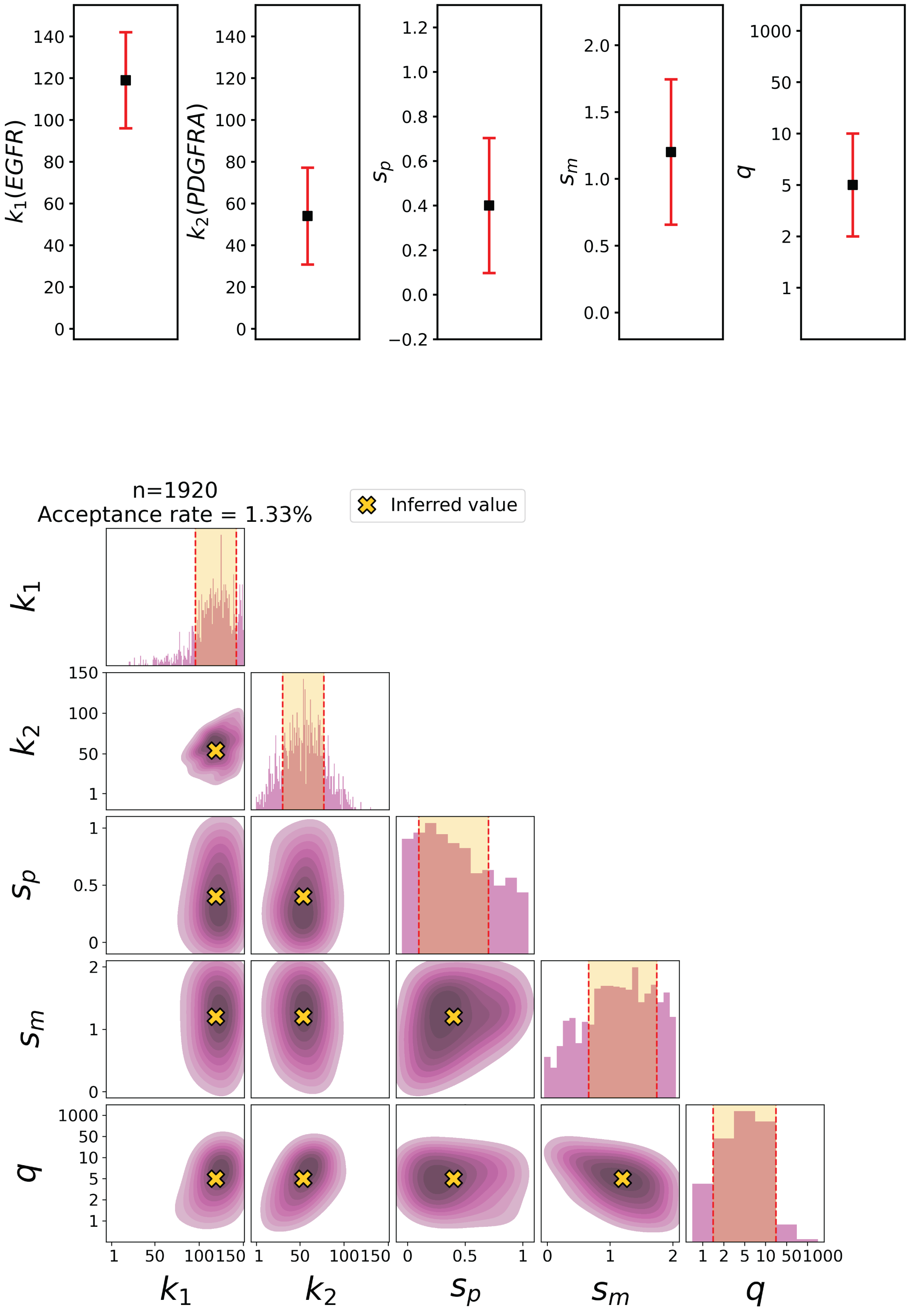

Supplementary Figure 26: Model inference summary for patient A5 using the co-amplified ecDNA simulation model. Model was fit to ecDNA copy number distributions for *EGFR*-amplifying and *PDGFRA*-amplifying ecDNA species. Parameters k_1 and k_2 describe the initial number of each ecDNA species, whilst s_p and s_m represent selection coefficients for tumor cells with 1 (pure) or 2 (mixed) ecDNA species respectively.

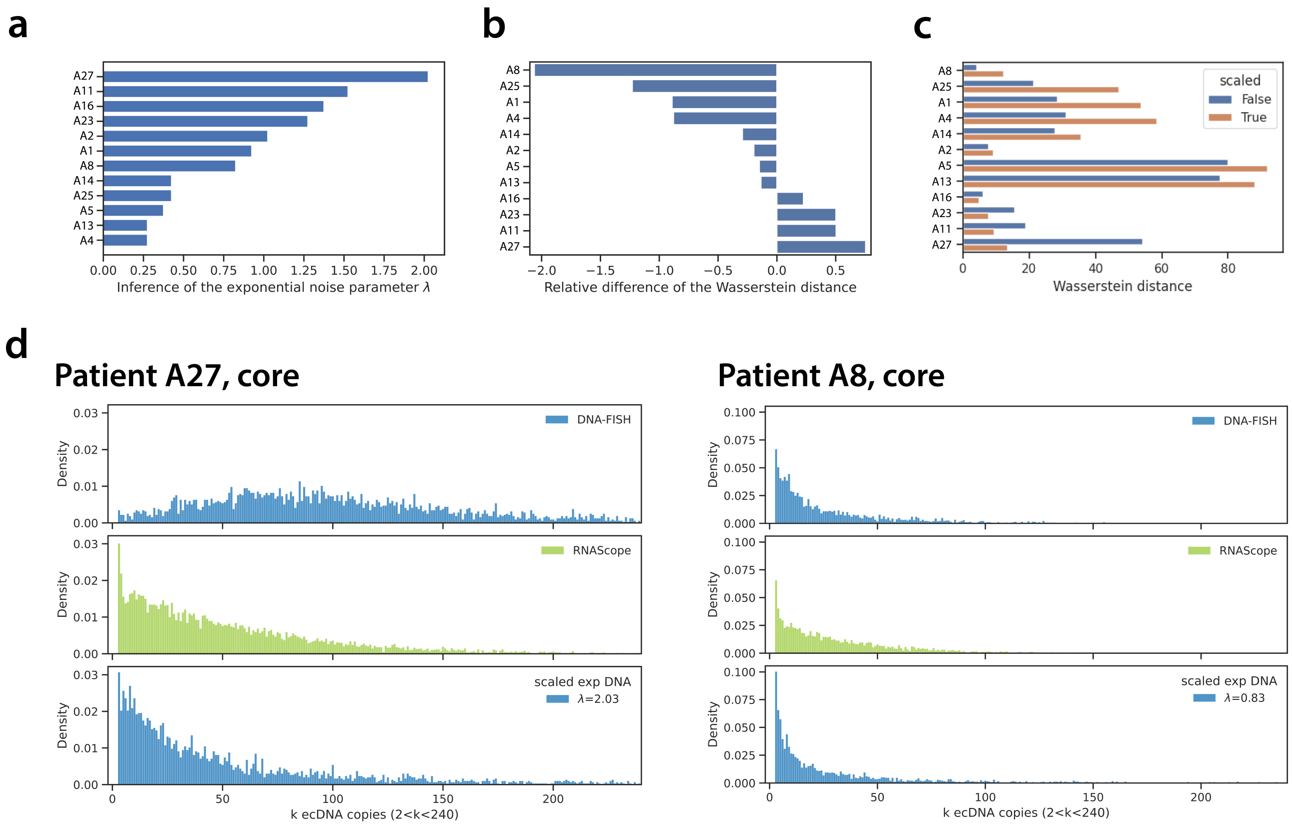

Supplementary Figure 27: Concordance of per-cell ecDNA distributions, derived with DNA FISH, and nascent RNAscope distributions for a range of patient samples. **(a)** Parameter of exponential noise kernel, λ, which provided best fitting transformation of DNA to RNA distributions. Best-fitting values were inferred using approximate Bayesian computation. **(b)** Difference between re-scaled DNA and RNA distributions, relative to distance between original DNA and RNA distributions. **(c)** Wasserstein distance between DNA and RNA distributions, before and after scaling of the DNA distribution. **(d)** Examples for two patients, A27 and A8, of the experimentally measured DNA and RNA distributions (top and middle row respectively) and the re-scaled DNA distribution (bottom).

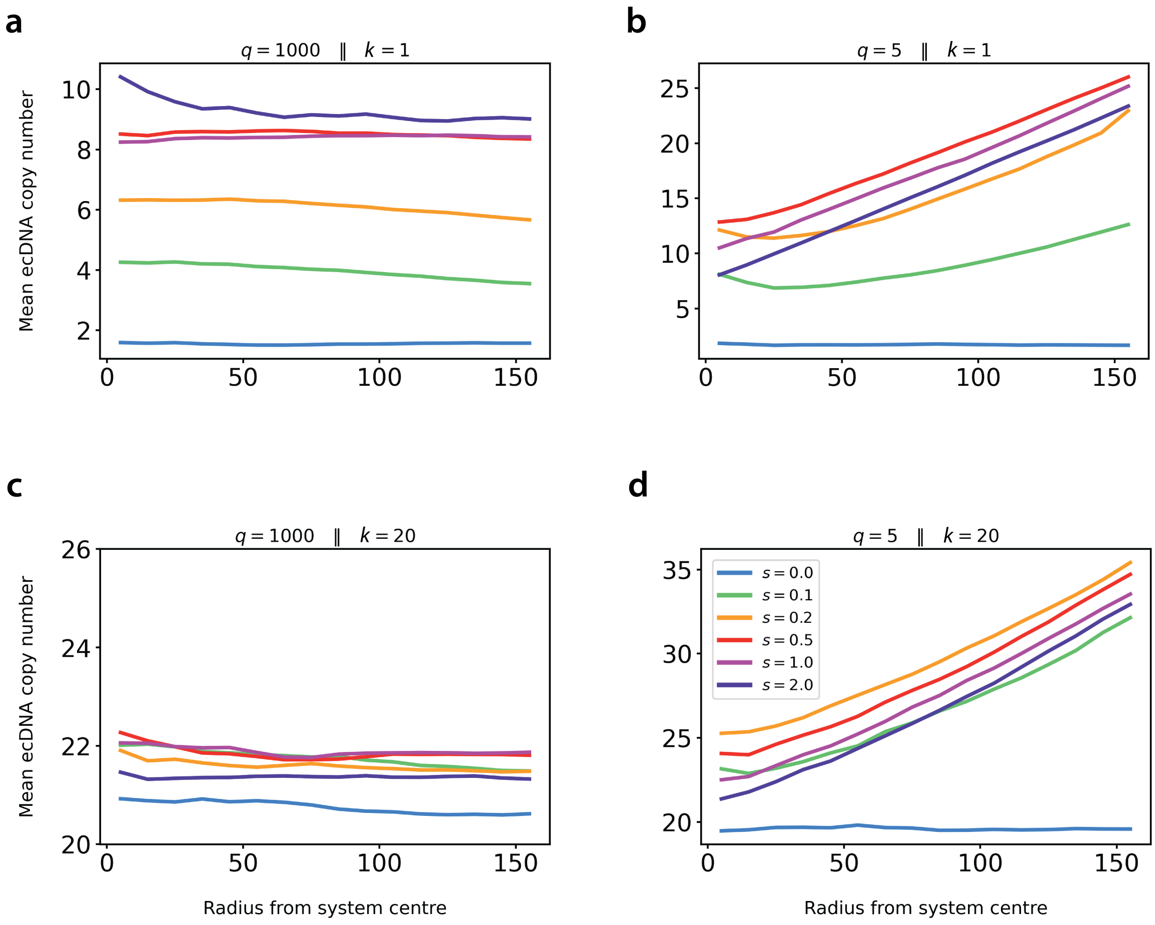

Supplementary Figure 28: Relationship between mean single-cell ecDNA copy number with distance from tumor center over a range of ecDNA fitness advantages, s for tumors with **(a)** $q = 1000$ & $k = 1$; **(b)** $q = 5$ & $k = 1$; **(c)** $q = 1000$ & $k = 20$; **(d)** $q = 5$ & $k = 20$. Data represent averaged values from 500 simulated tumors, final tumor size $N_{max} = 10^5$ cells.

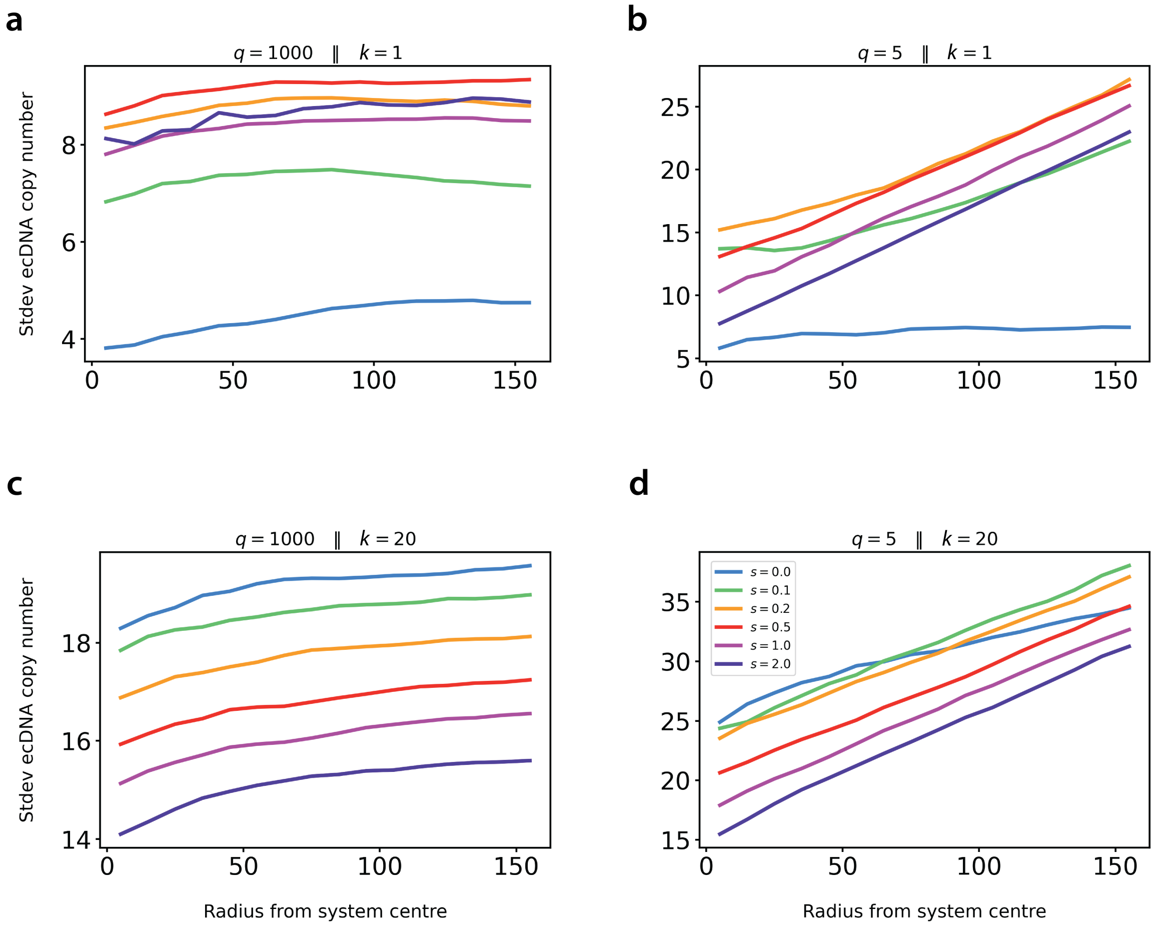

Supplementary Figure 29: Relationship between the standard deviation in single-cell ecDNA copy number with distance from tumor center over a range of ecDNA fitness advantages, s for tumors with **(a)** $q = 1000$ & $k = 1$; **(b)** $q = 5$ & $k = 1$; **(c)** $q = 1000$ & $k = 20$; **(d)** $q = 5$ & $k = 20$. Data represent averaged values from 500 simulated tumors, final tumor size $N_{max} = 10^5$ cells.

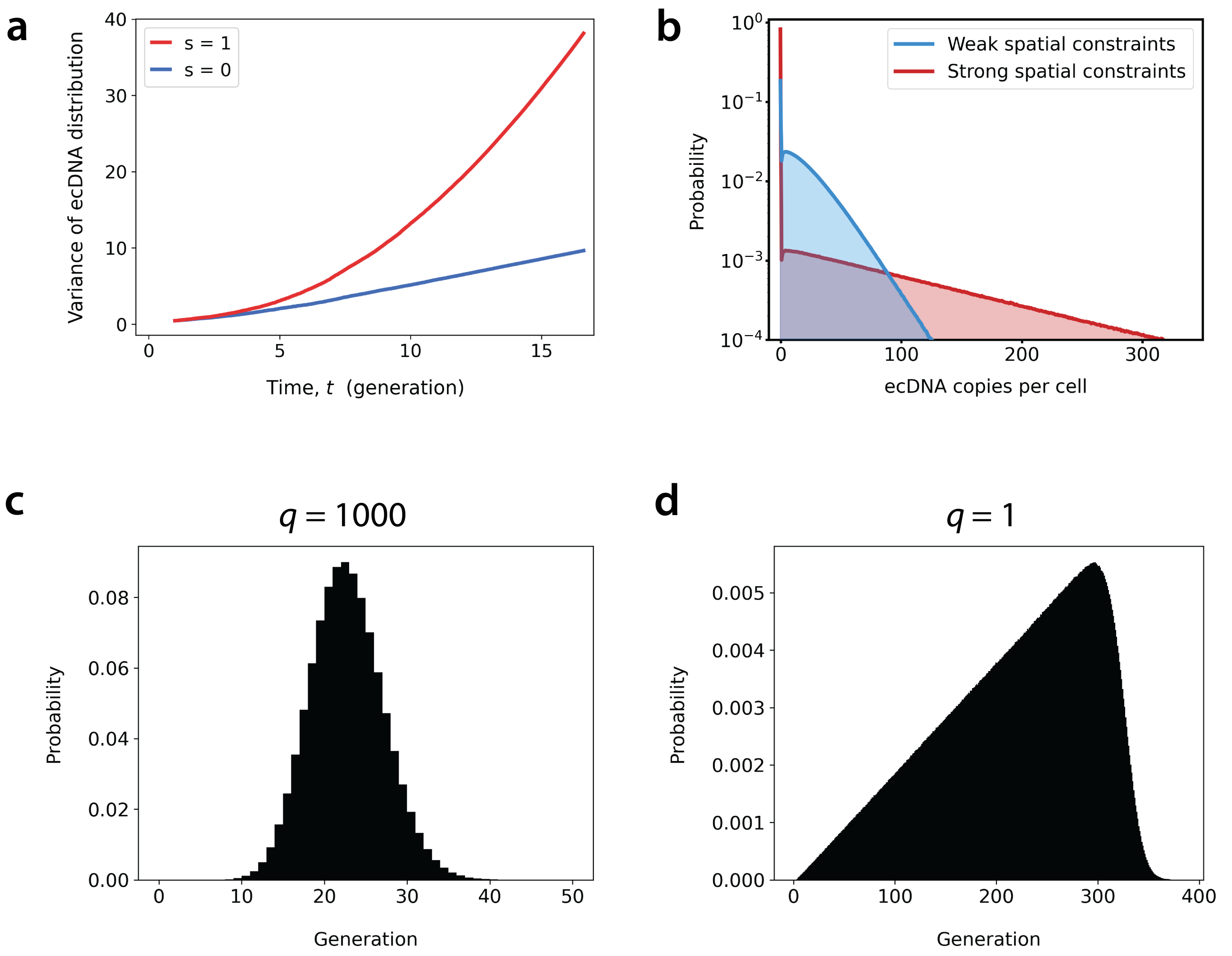

Supplementary Figure 30: **(a)** Variance in single-cell ecDNA copy number as a function of time for non-spatially constrained tumors (q =1,000) with initial ecDNA copy number $k = 1$. Variance scales linearly with time when ecDNA presence confers no fitness advantage ($s = 0$) and quadratically when ecDNA give rise to increased cell fitness ($s = 1$). **(b)** ecDNA copy number distribution for tumors with weak (q =1,000) versus strong ($q = 5$) spatial constraints ($k = 20$ & $s = 0$). Distributions represent combined data from 500 simulated tumors, final tumor size $N_{max} = 10^5$ cells. **(c-d)** Cell generation (number of previous cell divisions in cell lineage) distributions for tumors with **(c)** q =1,000 and **(d)** $q = 1$, simulated up to final a size $N_{max} = 10^5$ cells. Distributions represent combined data from 500 simulated tumors.

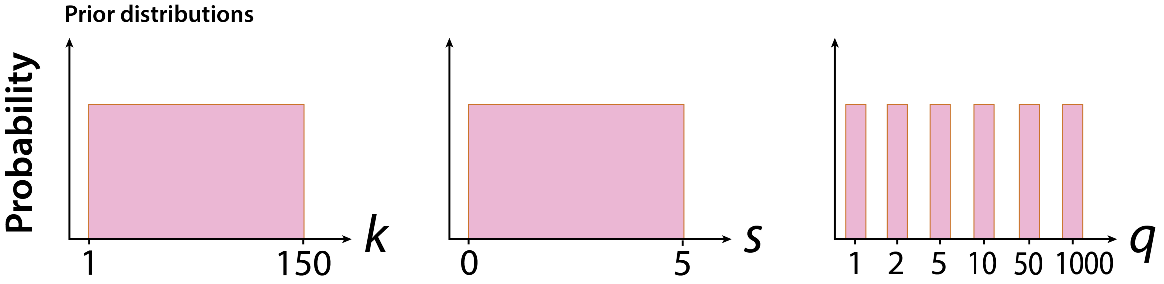

Supplementary Figure 31: Prior distributions for model parameters k, s and q, used for approximate Bayesian computation.

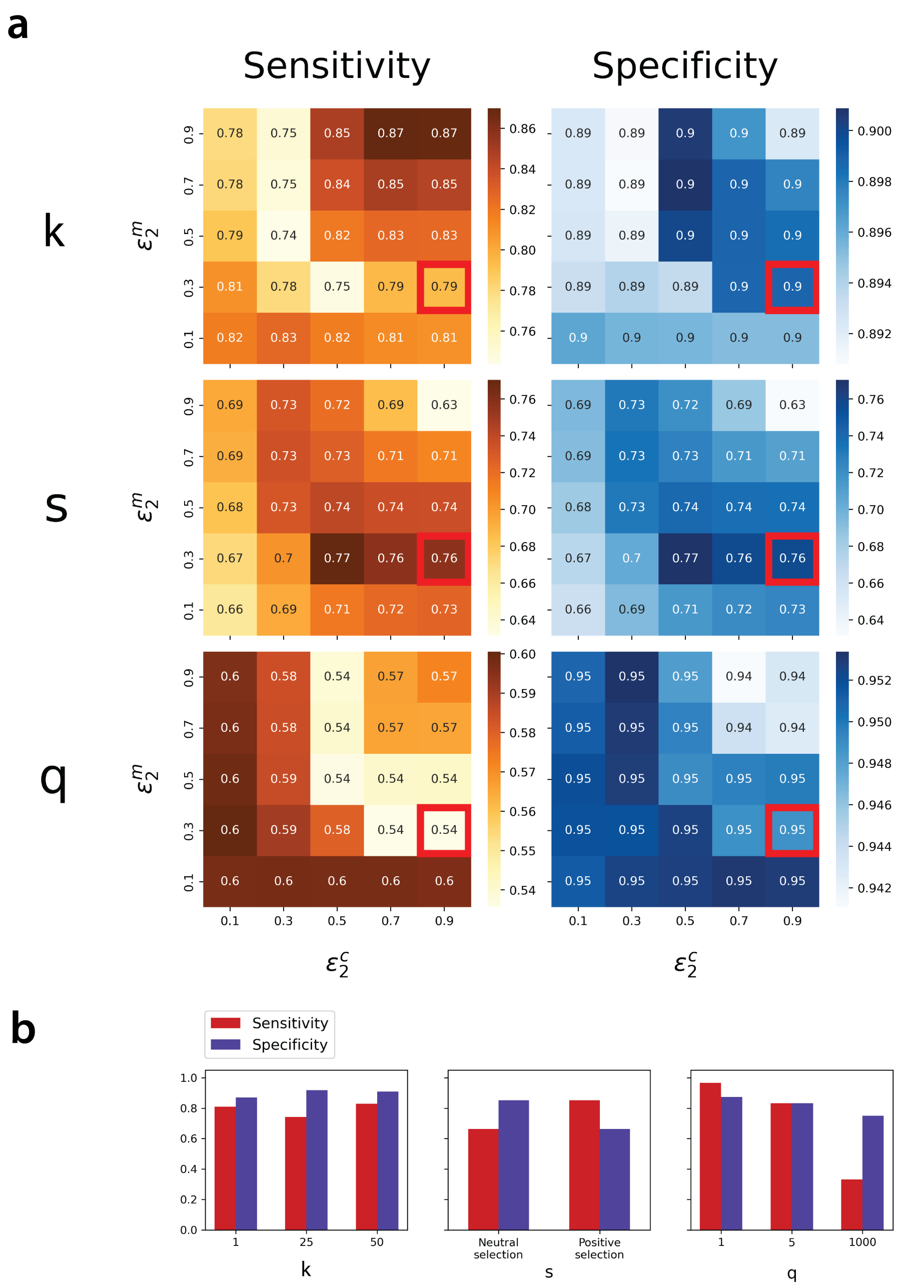

Supplementary Figure 32: **(a)** Averaged sensitivity and specificity of model inference algorithm using a range of threshold values on low-ecDNA cell fraction similarity, ϵ_2^c and ϵ_2^m, for the tumor core and margin respectively. Red boxes highlight combination of threshold values which maximized algorithm sensitivity and specificity. **(b)** Sensitivity (true positive rate) and specificity (true negative rate) of ABC inference algorithm tested across a range of model k, s and q values. The algorithm was considered to have predicted a given value if that value was contained within the interval of the point estimator $\pm$ error. Data were generated by running the ABC algorithm on artificial patient core and margin ecDNA data, generated themselves using the spatial model. Artificial patient dataset represented all possible combinations of $k \in \{1, 25, 50, 100\}$, $s \in \{0, 0.5, 1, 2\}$ and $q \in \{1, 5, 50, 1000\}$, with 100 patients per parameter combination.

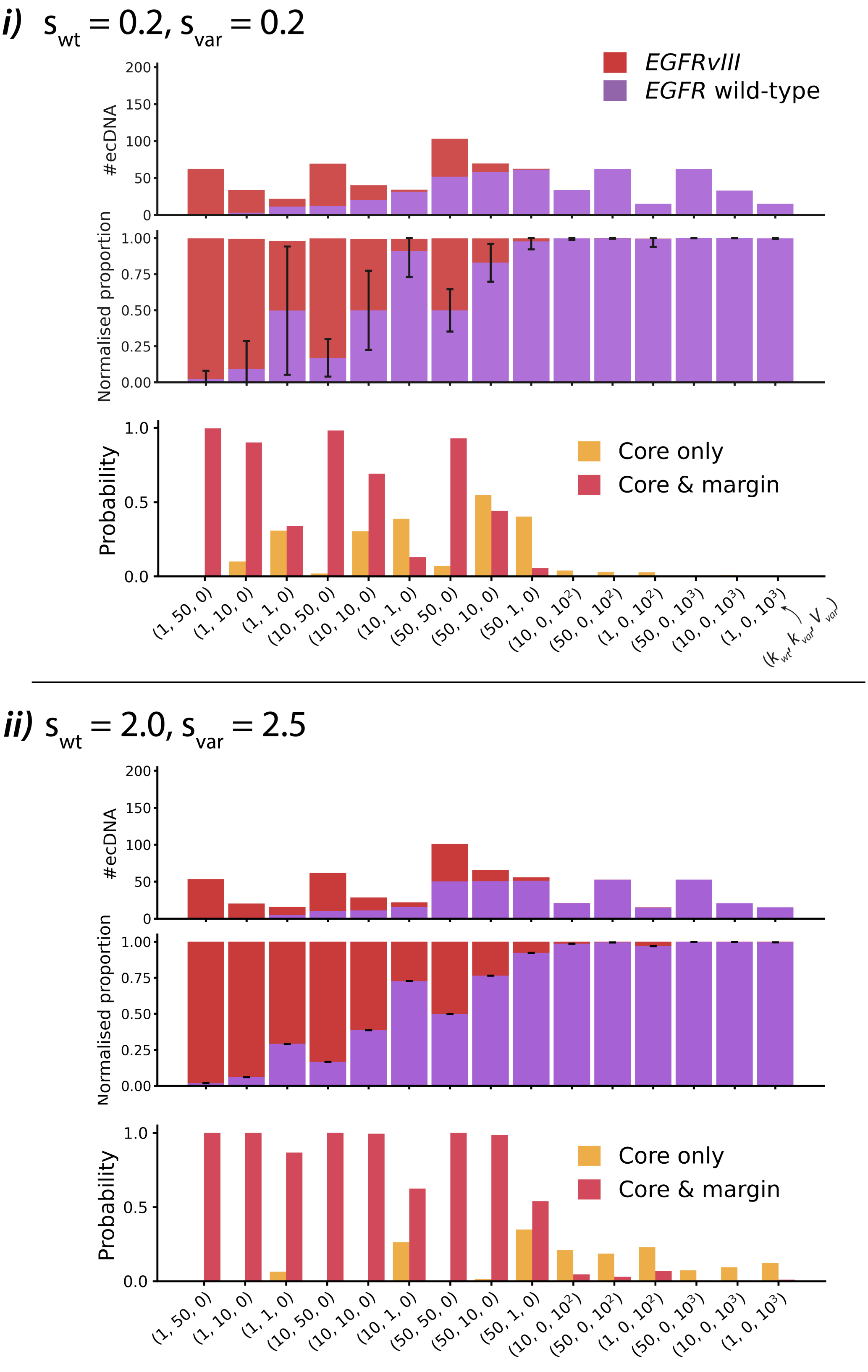

Supplementary Figure 33: ecDNA heteroplasmy in final tumor of $N_{max} = 10^5$ cells, simulated for a range of $(k_{wt},\ k_{var},\ V_{var})$ values with q = 2, and (i) $(s_{wt},\ s_{var})$ = (0.2, 0.2) and (ii) $(s_{wt},\ s_{var})$ = (2.0, 2.5). In each panel, top and middle rows contain absolute and percentage numbers, respectively, of ecDNA carrying wild-type *EGFR* or *EGFRvIII* (mean ± standard deviation). Bottom rows represent model predictions for the probability of observing *EGFRvIII* ecDNA across tumor core and margin, for a pair of core and random margin regions. Mean values derived from 1,000 simulated tumors.

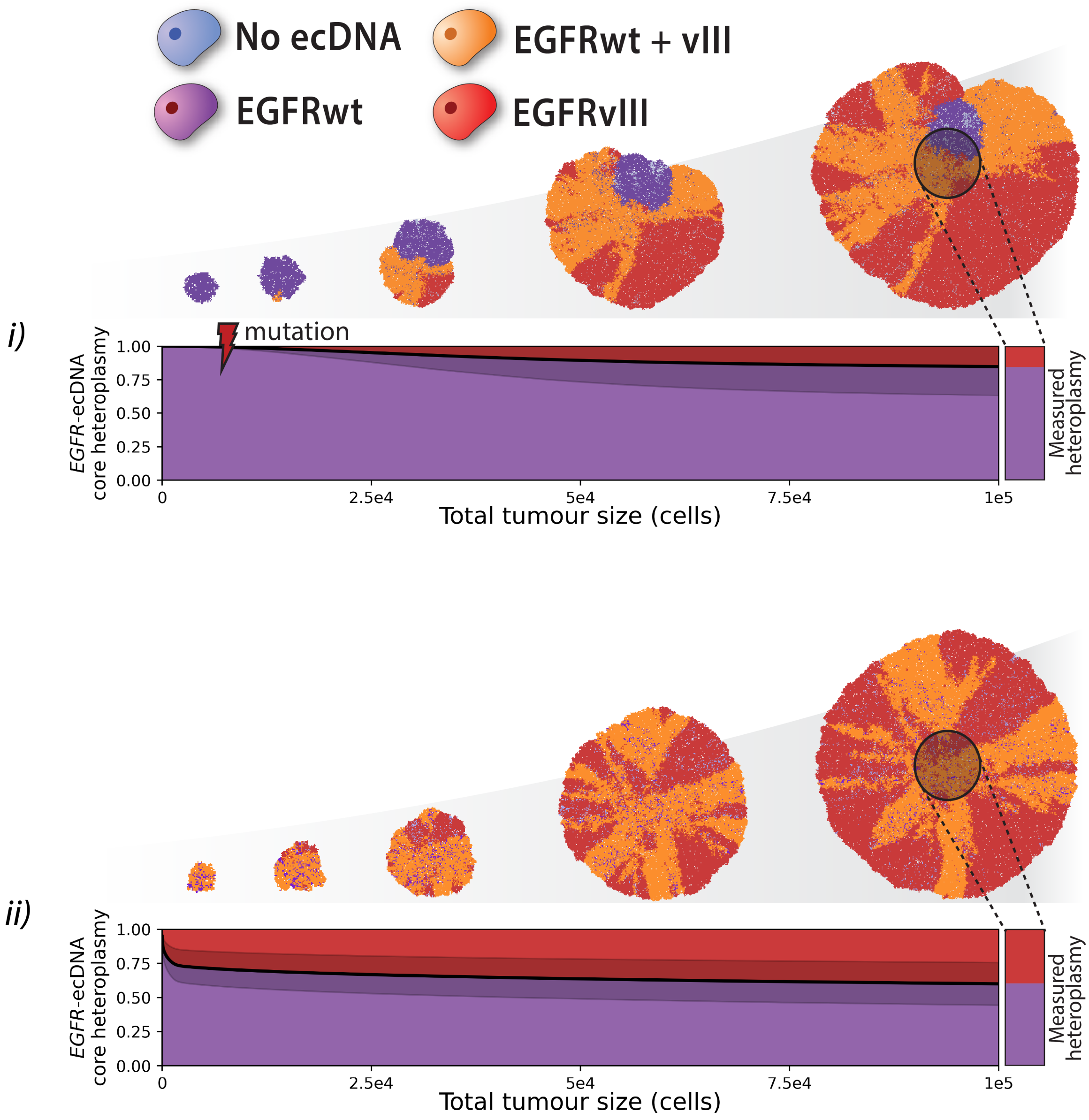

Supplementary Figure 34: Time-evolution of ecDNA heteroplasmy in tumor core for (i) $(k_{wt} = 50, k_{var} = 0, V_{var} = 1{,}000)$ and (ii) $(k_{wt} = 20, k_{var} = 1, V_{var} = 0)$. Final tumor size of $N_{max} = 10^5$ cells, and $s_{wt} = 0.2$, $s_{var} = 2$, $q = 2$. Upper row depicts time-evolution of single representative tumor; lower row represent mean $\pm$ standard deviation (shaded region) from 1,000 simulated tumors.
